# Two novel sites determine genetic relationships between CPV-2 and FPV: an epidemiological survey of canine and feline parvoviruses in Changchun, China (2020)

**DOI:** 10.3389/fvets.2024.1444984

**Published:** 2024-11-04

**Authors:** Zishu Li, Jiaxi Cai, Chuchu Feng, Yu Wang, Shuren Fang, Xianghong Xue

**Affiliations:** ^1^Department of Viral Infectious Diseases of Special Animals, Institute of Special Animal and Plant Sciences, Chinese Academy of Agricultural Sciences, Changchun, Jilin, China; ^2^Jilin Provincial Key Laboratory of Special Economic Animal Molecular Biology, Institute of Special Animal and Plant Sciences, Chinese Academy of Agricultural Sciences, Changchun, Jilin, China

**Keywords:** canine parvovirus, feline parvovirus, epidemiological surveys, VP2 gene, NS1 gene

## Abstract

Canine parvovirus (CPV-2) and feline parvovirus (FPV) cause severe hemorrhagic diarrhea disease in dogs, cats, and fur-bearing and wildlife carnivores worldwide, continuing to pose significant threats. In this study, 140 rectal swabs were collected from 70 domestic dogs and 70 cats with clinical diarrhea in veterinary clinics in Changchun during 2020. A total of 64.3% (45/70) of dogs and 55.7% (39/70) of cats tested positive for CPV-2 or FPV using colloidal gold strips. Amino acid (aa) sequence alignment of the VP2 protein from 39 CPV-2 and 36 FPV samples revealed that 79.5% (31/39) were CPV-2c, 17.9% (7/39) were a new CPV-2a, and 2.6% (1/39) were mink enteritis virus (MEV). and 8.3% (3/36) FPV from the cats was infected by CPV-2, which suggested that CPV-2c was the dominant variant in dogs and FPV was the major pathogen in cats in Changchun city. Phylogenetic relationships of VP2 genes showed that 26 parvoviruses were closely related to domestic strains previously published in China; however, 8 FPVs and CPV-JL-15/China/2020 were clustered in the lineage of South Asiatic and European countries, and 7 out of 8 FPVs were close to Italy. In addition to Q247H, I248Y, F544Y, and E/V545V/K, two novel site mutations of N23D or L630P in NS1 protein, associated with viral cross-species transmissions, were first found as a reminder of genetic relationships of CPV-2 variants and FPVs in the same branch. Thus, regular and massive virus surveillance of parvovirus is necessary to cope with its ongoing infection, circulation, mutations, and evolutions to new subtypes with strong survival abilities.

## 1 Introduction

CPV-2, FPV, and MEV belonged to the *Parvovirus* genus, *Parvoviridae* family, and were non-enveloped DNA viruses with a linear, single-stranded DNA genome that contained two major open reading frames (ORFs), encoding two non-structural proteins (NS1 and NS2) and two structural proteins (VP1 and VP2) through alternative mRNA splicing (1, 2). VP2 protein accounted for 90% of capsid and determined viral host range, antigenicity, and hemagglutination properties (3). The NS1 gene initiated viral genomic replication by attaching to the viral nicked DNA genomic 5′ end (4) and was included recently in the CPV-2 phylogenies analysis (1). The NS2 protein was the conjunction of left-hand 260-nt and right-hand 238-nt genetic fragments of the NS1 open reading frame but no studies on NS2 amino-acid divergences within its viral species (5).

CPV-2 and FPV caused hemorrhagic enteritis or anorexia infections in pets, fur-bearing, and wildlife animals and were spread around the world (6, 7). FPV was first identified in 1928, and its antigenic and biological characteristics were relatively stable (8). In contrast, CPV-2 has evolved through positive selections with high nucleotide mutation rates, like RNA viruses (9). In the late 1970s, CPV-2 emerged as a new virus in domestic dogs (10). Following its initial detection, CPV-2 swiftly reached pandemic proportions and was soon replaced by a novel antigenic and genetic variant, designated as CPV-2a (11, 12). CPV-2b was discovered in the United States in 1984 (13); in 1990, the S297A mutation appeared in VP2 of CPV-2a and CPV-2b, which were designated as new CPV-2a and new CPV-2b accordingly (14); and CPV-2c was identified in Italy in 2000 (15). Meanwhile, they caused cross-species infections in different host species (16). FPV is the main causative agent for feline panleukopenia (FPL), which can also be caused by CPV-2a, 2b, and 2c variants (17, 18). A 2022 report from Henan Province in China demonstrated that 98 out of 111 dogs were CPV-2 positive, and the CPV-2c was predominant, accounting for 91.84% of infection events, which was significantly higher than the new CPV-2a variant at 8.16% (3). Similarly, 60 out of 77 dogs were found infected with CPV-2, and of the 22 successfully isolated viruses, 17 were CPV-2c (77.3%), 4 were CPV-2a, and 1 was CPV-2b in Hebei Province in China in the same year (19). Among 346 dogs tested in Italy in 2022, 4% were CPV-2, 9% were CPV-2a, 18% were CPV-2b, and 69% were CPV-2c (20). In some African countries, CPV-2a and CPV-2c were cocirculating, with CPV-2a being the dominant strain. In 2019, eight vaccinated dogs in Gabon died from CPV-2 infections: Two were CPV-2c and six were CPV-2a (21). Regarding felines, FPV affected all aged cats, and kittens were particularly vulnerable with a 90% mortality rate (22, 23). In addition, an investigation in 2017 in Australia reported that 24 domestic cats, despite being vaccinated with the attenuated FPV vaccine, were re-infected with FPV (24). MEV is considered to be closely related to, or almost identical to, FPV (25, 26). Genomic analysis suggests that MEV and CPV share a close relationship, with both viruses known to cause acute enteritis. In 2010, evidence of recombination between MEV and CPV was identified in a strain isolated from a farm in Liaoning Province, China (27).

The frequency and complexity of mutations of parvovirus have increased its spillover infections from domestic animals to wildlife species (22). CPV-2 was detected in China’s giant pandas in 2010 (28), new CPV-2a also infected giant pandas in 2015 (29), and subsequently, two juvenile pandas died of FPV infection in 2020 in China (30). Two pangolins with severe diarrhea died of CPV-2c infection within a week in Taiwan in 2022 (31). CPV-2a was the main variant in the Serbian (32) and Iranian dogs (33), and we observed cross-host transmission between dogs and newfoundland coyotes in Canada (34). In Turkey, two strains of CPV-2b were also identified in a random sample of 630 red foxes (35). The main prevalence and cross-host transmission of CPV-2c variants existed in Namibia between jackals and dogs (36). This growing evidence emphasized the importance of surveillance for parvoviruses to control circulation, epidemics, and transmission. In this study, we demonstrated an epidemiological survey of CPV-2 and FPVs in Changchun City, China, in 2020 by characterizing the nucleotide sequences and key aa sites of VP2 and NS1 proteins of CPV-2 and FPV, which aimed to provide some new information on the prevalence of CPV-2 variants and FPV.

## 2 Materials and methods

### 2.1 Sample collection

The sample population included 70 domestic dogs and 70 cats, mostly aged 2 months, and non-vaccinations with gastroenteritis signs (diarrhea, vomiting, and/or apocleisis) from three veterinary clinics in Changchun, Jilin Province, China, in 2020. A total of 140 rectal swab samples were collected using the Test Card for Canine/Feline Parvovirus Antigen Detections (Sinorp, China). The dog and cat sample collections were consented to by the pet owners and were carried out strictly in accordance with the guidelines of the Animal Care and Use Committee of the Institute of Special Animal and Plant Sciences. The rectal swabs were immersed in sterile phosphate-buffered saline (PBS) with 10% antibiotic solution. The supernatant was collected after centrifuging 8,000 × g for 10 min at 4°C and kept at-80°C for later investigations.

### 2.2 Polymerase amplification of VP2 and NS1 genes

Viral DNAs from antigen-positive rectal swabs were extracted using the EasyPure Viral DNA/RNA Kit (TransGen, Beijing, China) according to the manufacturer’s instructions. The specific primers were designed with Soft Primer 7.0 to amplify VP2 and NS1 genes of FPVs and CPV-2 s by PCR assay (Table 1). The obtained PCR products of VP2 and NS1 genes were identified by 1% agarose gel electrophoresis (data not shown) and subsequently purified with the EasyPure Quick Gel Extraction Kit (TransGen, Beijing, China) and ligated to the pEasy-Blunt vector (TransGen, Beijing, China). The ligated products were transformed into Trans1-T1 competent cells (TransGen, Beijing, China) and inoculated onto AGAR plates supplemented with ampicillin (100 mg/mL). Finally, the positive plasmids were extracted and identified by double restriction enzyme digestion of *Kpn* I and *Not* I. Furthermore, we used PCR assays to detect canine coronavirus infection, which was associated with diarrhea (1).

**Table 1:** Primers used for amplification of VP2 and NS1 genes.

Gene category	Primers sequences (5′–3′)	Fragment size (bp)	Annealing temperatures
VP2- For	CGCTGATCCATGAGTGATCGAGCAGTTC	1755	49°C
VP2- Rev	CCCGAGCTTTTAATATAATTTTCTAAGTG		
NS1-For	GACCGTTACTGACATTCGC	2007	51°C
NS1- Rev	CGGCGTCAGAAGGGTT		

For indicated forward primer, and Rev meant reverse primer.

### 2.3 Referenced sequence data collection

To analyze the global distribution of VP2 genes in parvoviruses, 2,181 VP2 sequences (1,755 bp), published before 5 April 2023, were retrieved and downloaded from the NCBI GenBank database. These sequences included information such as the country or region and the date of sample collection and were further classified by location (Supplementary Table S2). In addition, 129 NS1 genes (25 FPV and 104 CPV-2, 2007 bp) published before the same date were also retrieved.

### 2.4 Sequencing, phylogenetic analysis, and recombination analysis

Positive plasmids containing the obtained VP2 and NS1 genes in this study were sequenced at least three times for each sample to eliminate possible errors during the sequencing process using the Sanger method of DNA sequencing (Sangon Biotech, Shanghai, China). The final sequences of 35 VP2 genes (19 from CPV-2 and 16 from FPVs) and 28 NS1 genes (11 from CPV-2 s and 17 from FPVs) were all deposited in the NCBI GenBank database (Tables 2, 3). Phylogenetic trees based on the VP2 genes (1755 bp in length) and NS1 genes (2007 bp in length) were established, respectively, using the neighbor-joining (NJ) method using MEGA 11, and the bootstrap validation method with 1,000 replications, and the final figure was edited with Figtree (FigTree v1.4.3, n.d.).

**Table 2:** Comparisons of the key amino acid residues in VP2 proteins.

Virus Name	GenBank Accession No.	Mutations and positions sites: amino acid residue								Genotype
		80	93	103	297	323	426	564	568	
FPV/Raccoon/TX/Rac3/1978	KM624023	K	K	V	S	N	N	N	A	FPV
HRB-F8	KT156836	R	N	A	A	N	E	S	G	New CPV-2a
12B	PP049248	R	N	A	A	N	N	S	G	CPV-2c
Shangdong3	GU392247	K	K	V	S	D	N	N	A	MEV
CPV-JL-01/China/2020	OQ718428	R	N	A	S	N	E	S	G	CPV-2c
CPV-JL-02/China/2020	PP049252	R	N	A	A	N	E	S	G	CPV-2c
CPV-JL-03/China/2020	PP049253	R	N	A	A	N	E	S	G	CPV-2c
CPV-JL-04/China/2020	PP049254	R	N	A	A	N	E	S	G	CPV-2c
CPV-JL-05/China/2020	PP049256	R	N	A	A	N	E	S	G	CPV-2c
CPV-JL-06/China/2020	PP049257	R	N	A	A	I	D	N	M	CPV-2c
CPV-JL-07/China/2020	PP049258	R	N	A	L	N	E	S	G	CPV-2c
CPV-JL-08/China/2020	PP049259	R	N	A	A	N	E	S	G	CPV-2c
CPV-JL-09/China/2020	PP049260	R	N	A	A	N	E	S	G	CPV-2c
CPV-JL-10/China/2020	PP049261	R	N	A	L	N	E	S	G	CPV-2c
CPV-JL-11/China/2020	PP049262	R	N	A	A	N	E	S	G	CPV-2c
CPV-JL-12/China/2020	PP049263	R	N	A	L	N	N	S	G	New CPV-2a
CPV-JL-13/China/2020	PP049264	R	N	A	A	N	E	S	G	CPV-2c
CPV-JL-14/China/2020	PP049265	R	N	A	A	N	N	S	G	New CPV-2a
CPV-JL-15/China/2020	PP049266	R	N	A	A	N	N	S	G	New CPV-2a
CPV-JL-16/China/2020	PP049267	R	N	A	A	N	E	S	G	CPV-2c
CPV-JL-17/China/2020	PP049268	R	N	A	A	N	E	S	G	CPV-2c
CPV-JL-18/China/2020	PP049269	R	N	A	A	N	E	S	G	CPV-2c
CPV-JL-19/China/2020	PP049255	R	N	A	S	N	N	N	G	MEV
FPV-JL-01/China/2020	PP049270	K	K	V	S	D	N	N	A	FPV
FPV-JL-02/China/2020	PP049271	K	K	V	S	D	N	N	A	FPV
FPV-JL-03/China/2020	PP049272	K	N	V	A	N	N	S	G	New CPV-2a
FPV-JL-04/China/2020	PP049273	R	K	V	S	D	N	N	A	FPV
FPV-JL-05/China/2020	PP049274	K	K	V	S	D	N	N	A	FPV
FPV-JL-06/China/2020	PP049275	K	K	V	S	D	N	N	A	FPV
FPV-JL-07/China/2020	PP049276	R	N	A	A	N	E	S	G	CPV-2c
FPV-JL-08/China/2020	PP049277	K	K	V	S	D	N	N	A	FPV
FPV-JL-09/China/2020	PP049278	K	K	V	S	D	N	N	A	FPV
FPV-JL-10/China/2020	PP049279	K	K	V	S	D	N	N	A	FPV
FPV-JL-11/China/2020	PP049280	K	K	V	S	D	N	N	A	FPV
FPV-JL-12/China/2020	PP049281	K	K	V	S	D	N	N	A	FPV
FPV-JL-13/China/2020	PP049282	K	K	V	S	D	N	N	A	FPV
FPV-JL-14/China/2020	PP049283	K	K	V	S	D	N	N	A	FPV
FPV-JL-15/China/2020	PP049284	R	N	A	A	N	N	S	G	New CPV-2a
FPV-JL-16/China/2020	PP049285	K	K	V	S	D	N	N	A	FPV

The strains of FPV/Raccoon/TX/Rac3/1978, HRB-F8, 12B and Shangdong3 were reference strains.

**Table 3:** Comparison of all the key amino acid residues in NS1 protein.

Virus Name	Genbank accession No.	23	85	178	247	248	358	544	545	589	630
FPV-SX	MT892651.1	D	A	I	H	T	D	Y	E	N	L
FPV_175_A_AUS_Syd_04/2017	OM867680.1	N	A	I	H	T	D	Y	E	N	L
JN-FPV-12	MZ836453.1	N	A	I	Q	T	D	Y	E	N	L
JN-FPV-87	MZ836452.1	N	A	I	Q	T	D	Y	E	N	L
JN-FPV-90	MZ836451.1	N	A	I	Q	T	D	Y	E	N	L
JN-FPV-91	MZ836450.1	N	A	I	Q	T	D	Y	K	N	L
TZ-FPV-99	MZ836448.1	N	A	I	Q	I	D	Y	E	N	L
TZ-FPV-104	MZ836447.1	N	A	I	Q	T	D	Y	K	N	L
TZ-FPV-108	MZ836446.1	N	A	I	Q	I	D	Y	E	N	L
TZ-FPV-112	MZ836445.1	N	A	I	Q	T	D	Y	E	N	L
TZ-FPV-119	MZ836444.1	D	A	I	H	T	D	Y	E	N	L
TZ-FPV-122	MZ836443.1	N	A	I	Q	T	D	Y	E	N	L
TZ-FPV-124	MZ836442.1	N	A	I	Q	T	D	Y	E	N	L
TZ-FPV-135	MZ836441.1	D	A	I	H	T	D	Y	E	N	L
TZ-FPV-143	MZ836440.1	N	A	I	Q	I	D	F	E	N	L
TZ-FPV-148	MZ836439.1	N	A	I	H	I	D	Y	E	N	L
TZ-FPV-185	MZ836438.1	D	A	I	H	T	D	Y	E	N	L
TZ-FPV-193	MZ836437.1	N	A	I	Q	I	D	Y	E	N	L
TZ-FPV-195	MZ836436.1	N	A	I	Q	T	D	Y	E	N	L
TZ-FPV-235	MZ836435.1	D	A	I	H	T	D	Y	E	N	L
TZ-FPV-237	MZ836434.1	D	A	I	H	T	D	Y	E	N	L
TZ-FPV-242	MZ836433.1	N	A	I	Q	T	D	Y	E	N	L
TZ-FPV-243	MZ836432.1	D	A	I	H	T	D	Y	E	N	L
JN-FPV-96	MZ836429.1	D	A	I	H	T	D	Y	E	N	L
JN-FPV-11	MZ836428.1	D	A	I	H	T	D	Y	E	N	L
FPV-JL-1	PP049297	D	A	I	H	T	D	Y	E	N	L
FPV-JL-05/China/2020	PP049298	N	A	I	H	T	D	Y	E	N	L
FPV-JL-06/China/2020	PP049299	D	A	I	H	T	D	Y	E	N	L
FPV-JL-07/China/2020	PP049300	N	A	I	Q	I	D	F	V	N	P
FPV-JL-2	PP049301	D	A	I	H	T	D	Y	E	N	L
FPV-JL-3	PP049302	D	A	I	H	T	D	Y	E	N	L
FPV-JL-4	PP049303	D	A	I	H	T	D	Y	E	N	L
FPV-JL-08/China/2020	PP049304	N	A	I	H	T	D	Y	E	N	L
FPV-JL-09/China/2020	PP049305	D	A	I	H	T	D	Y	E	N	L
FPV-JL-5	PP049306	D	A	T	H	T	D	Y	E	N	L
FPV-JL-6	PP049307	D	A	I	H	T	D	Y	E	N	L
FPV-JL-7	PP049308	D	A	I	H	T	D	Y	E	N	L
FPV-JL-14/China/2020	PP049309	D	A	I	H	T	D	Y	E	N	L
FPV-JL-8	PP049310	D	A	I	H	T	D	Y	E	N	L
FPV-JL-9	PP049311	D	A	I	H	T	D	Y	E	N	L
FPV-JL-16/China/2020	PP049312	D	A	I	H	T	N	Y	E	N	L
FPV-JL-10	PP049313	D	T	I	H	T	D	Y	E	N	L
India strain	GQ421597.1	N	A	I	Q	I	D	F	E	N	L
TZ-CPV2c-131	MZ836379.1	N	A	I	Q	I	D	F	V	N	P
TZ-CPV2c-126	MZ836380.1	N	A	I	Q	I	D	F	V	N	P
JN-CPV2c-9	MZ836381.1	N	A	I	Q	I	D	F	V	N	P
HD-CPV2c-23	MZ836383.1	N	A	I	Q	I	D	F	V	N	P
XA-CPV2c-0-14	MZ836384.1	N	A	I	Q	I	D	F	V	N	P
XA-CPV2c-0-13	MZ836385.1	N	A	I	Q	I	D	F	V	N	P
XA-CPV2c-0-12	MZ836386.1	N	A	I	Q	I	D	F	V	N	P
XA-CPV2c-0-11	MZ836387.1	N	A	I	Q	I	D	F	V	N	P
XA-CPV2c-0-10	MZ836388.1	N	A	I	Q	I	D	F	V	N	P
XA-CPV2c-0-8	MZ836389.1	N	A	I	Q	I	D	Y	E	N	L
XA-CPV2c-0-7	MZ836390.1	N	A	I	Q	I	D	F	V	N	P
XA-CPV2c-0-6	MZ836391.1	N	A	I	Q	I	D	F	V	N	P
XA-CPV2c-0-3	MZ836392.1	N	A	I	Q	I	D	F	V	N	P
XA-CPV2c-0-1	MZ836393.1	N	A	I	Q	I	D	F	V	N	P
TZ-newCPV2b-150	MZ836394.1	N	A	I	Q	I	D	F	V	N	L
TZ-newCPV2a-149	MZ836395.1	N	A	I	Q	I	D	Y	E	N	L
TZ-newCPV2a-142	MZ836396.1	N	A	I	Q	I	D	F	V	N	P
TZ-newCPV2a-132	MZ836397.1	N	A	I	Q	I	D	Y	E	N	L
TZ-newCPV2a-127	MZ836398.1	N	A	I	Q	I	D	Y	E	N	L
TZ-CPV2c-174	MZ836400.1	N	A	I	Q	I	D	Y	E	N	L
TZ-CPV2c-173	MZ836401.1	N	A	I	Q	I	D	F	V	N	P
TZ-CPV2c-171	MZ836402.1	N	A	I	Q	I	D	Y	E	N	L
TZ-CPV2c-165	MZ836403.1	N	A	I	Q	I	D	Y	E	N	L
TZ-CPV2c-164	MZ836404.1	N	A	I	Q	I	D	F	V	N	P
TZ-CPV2c-152	MZ836405.1	N	A	I	Q	I	D	F	V	N	P
CPV-JL-03/China/2020	PP049286	N	A	I	Q	I	D	F	V	N	P
CPV-JL-08/China/2020	PP049287	N	A	I	Q	I	D	F	V	N	P
CPV-JL-1	PP049288	N	A	I	Q	I	D	F	V	E	P
CPV-JL-2	PP049289	N	A	I	Q	I	D	F	V	N	P
CPV-JL-3	PP049290	N	A	I	Q	I	D	F	V	N	P
CPV-JL-15/China/2020	PP049291	D	A	I	H	T	D	Y	E	N	L
CPV-JL-16/China/2020	PP049292	N	A	I	Q	I	D	Y	E	N	L
CPV-JL-4	PP049293	N	A	I	Q	I	D	F	V	N	P
CPV-JL-5	PP049294	N	A	I	Q	I	D	F	V	N	P
CPV-JL-6	PP049295	N	A	I	Q	I	D	F	V	N	P
CPV-JL-7	PP049296	N	A	I	Q	I	D	Y	E	N	P

Virus names beginning with CPV\FPV-JL represent the NS1 gene obtained in this study, and the remaining 50 NS1 genes were selected from 129 downloaded.

All the obtained VP2 and NS1 gene sequences in this study were aligned using MEGA 11 software (Supplementary Table S2) and screened to detect any possible recombination among sequences using Software RDP4 (containing various recombination detection methods: RDP, GENECONV, BootScan, MaxChi, Chimaera, SiScan, and 3Seq). The highest acceptable *p*-value was set at 0.05.

## 3 Results

### 3.1 The CPV-2c is the most prevalent strain with cross-infection between cats and dogs in Changchun city, China

A total of 45 out of 70 dogs with diarrhea tested positive for CPV-2 using the CPV-2 colloidal gold test strip, with a positive rate of 64.3%. Among the 70 suspected cat cases, 39 (55.7%) were FPV-positive by FPV colloidal gold test strip. We successfully isolated 39 VP2 genes from 45 CPV-2-positive dog samples and 36 VP2 genes from 39 FPV-positive cat samples by PCR assays (data not shown). However, due to the limited sample amount, amplification failures, and the presence of identical sequences, 34 VP2 gene sequences (19 CPV-2 and 16 FPVs) with 1755 bp in length were ultimately obtained. The CPV-2 sub-genotypes were determined based on the previously reported key aa residues in VP2 protein at 5, 87, 93, 103, 297, 323, 370, 426, 564, and 568 sites (37, 38). As shown in Table 2, new CPV-2a (297A, 426 N), CPV-2c (426E), and CPV-2a (297S, 426 N) strains were cocirculating in Changchun City, China. No original CPV-2, CPV-2b (297S, 426D), or new CPV-2b (297A, 426D) variants were detected. FPV was characterized by 80 K, 93 K, 103 V, 323D, 564 N, and 568A (39). On this basis, 13 FPV strains were identified, and 3 strains were identified as new CPV-2a and CPV-2c (FPV-JL-03/China/2020, FPV-JL-15/China/2020, and FPV-JL-07/China/2020) in this study; four out of five new CPV-2a showed a 440 T to A mutation in the VP2 protein, which was consistent with the previous report. However, the new CPV-2a FPV-JL-15/China/2020 strain did not have a 440 T to A mutation. It is noteworthy that the genotype of CPV-JL-19/China/2020 was initially considered to be CPV-2a based on 297S and 426 N. However, in recent years, all CPV-2a strains had been replaced by new CPV-2a variants, and CPV-JL-19/China/2020 had 87 M and 555I, indicating CPV-JL-19/China/2020 was neither CPV-2a nor CPV-2 vaccine shedding. Based on VP2 phylogenetic analysis, CPV-JL-19/China/2020 clustered with MEV with a 99.32% homologous rate to the Shandong3 MEV strain (GU392247) isolated from mink in Shandong Province, China, in 2007. In addition, CPV-JL-19/China/2020 differed from MEV strains with E75K, Q159H, D178H, and Q242H mutations, based on comparison with 41 VP2 gene sequences of MEV. We suspect that these mutations enabled MEV to infect dogs. Taken together, aa sequence alignment of VP2 protein from 39 CPV-2 and 36 FPV revealed that 79.5% (31/39) was CPV-2c, 17.9% (7/39) was new CPV-2a, 2.6% (1/39) was MEV, and 8.3% (3/36) FPV from the cats was infected by CPV-2. In addition, seven cases were co-infected with canine coronavirus (CcoV), with a positive rate of 17.9% (7/39).

### 3.2 Two novel sites in NS1 protein helped determine the genetic relationship between CPV-2 and FPV

**Figure 1:**
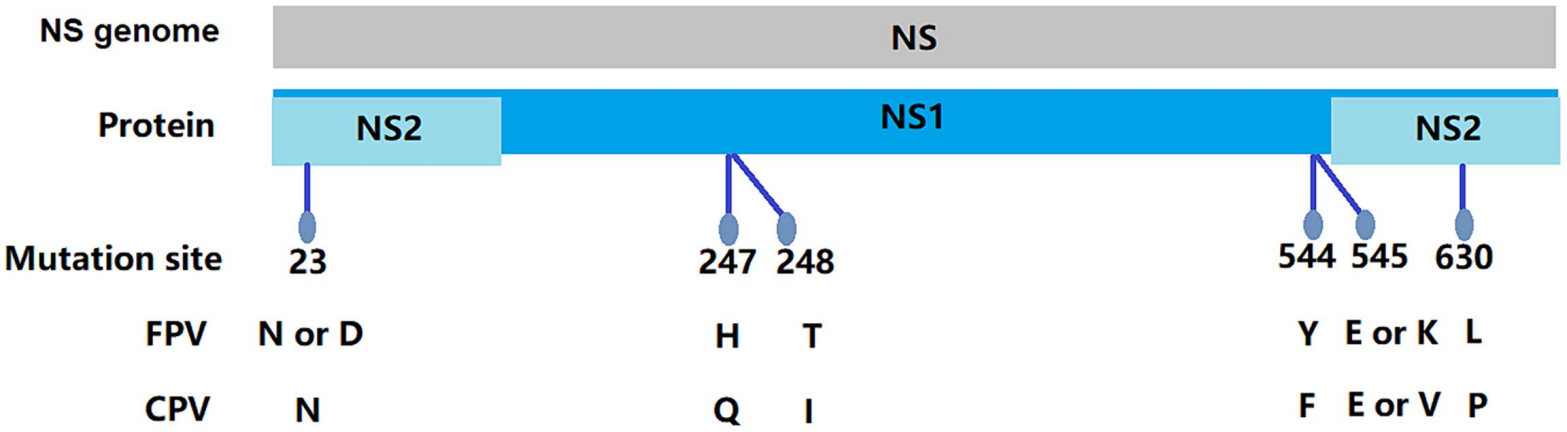
Molecular schematics of the NS gene of FPV/CPV.

**Figure 2:**
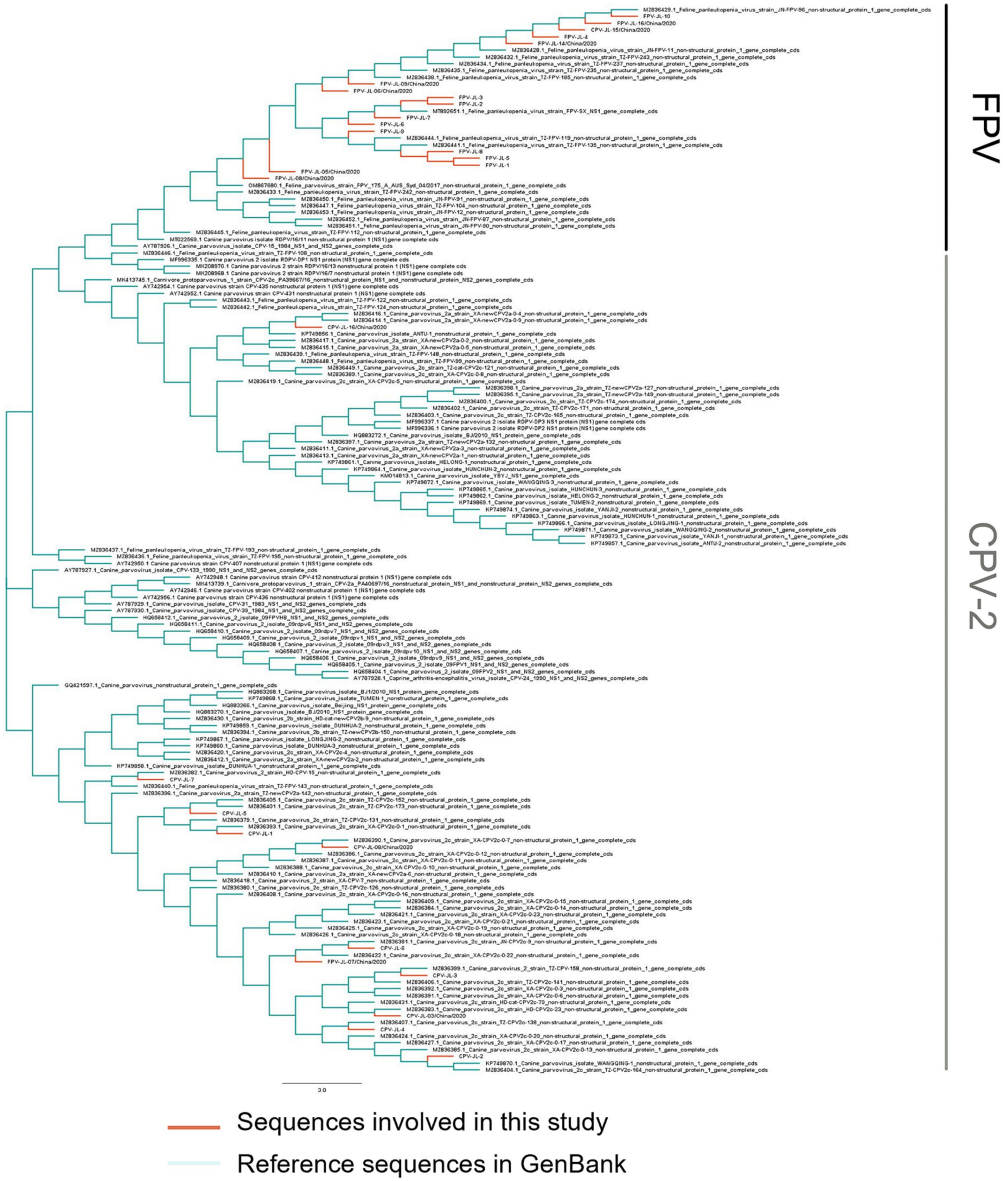
Phylogenetic tree based on the NS1 gene of CPV-2 variants and FPVs. Phylogenetic relationships were calculated using the neighbor-joining (NJ) method. The viruses obtained in this study are in red, and the reference CPV-2 variants and FPVs are in green.

**Figure 3:**
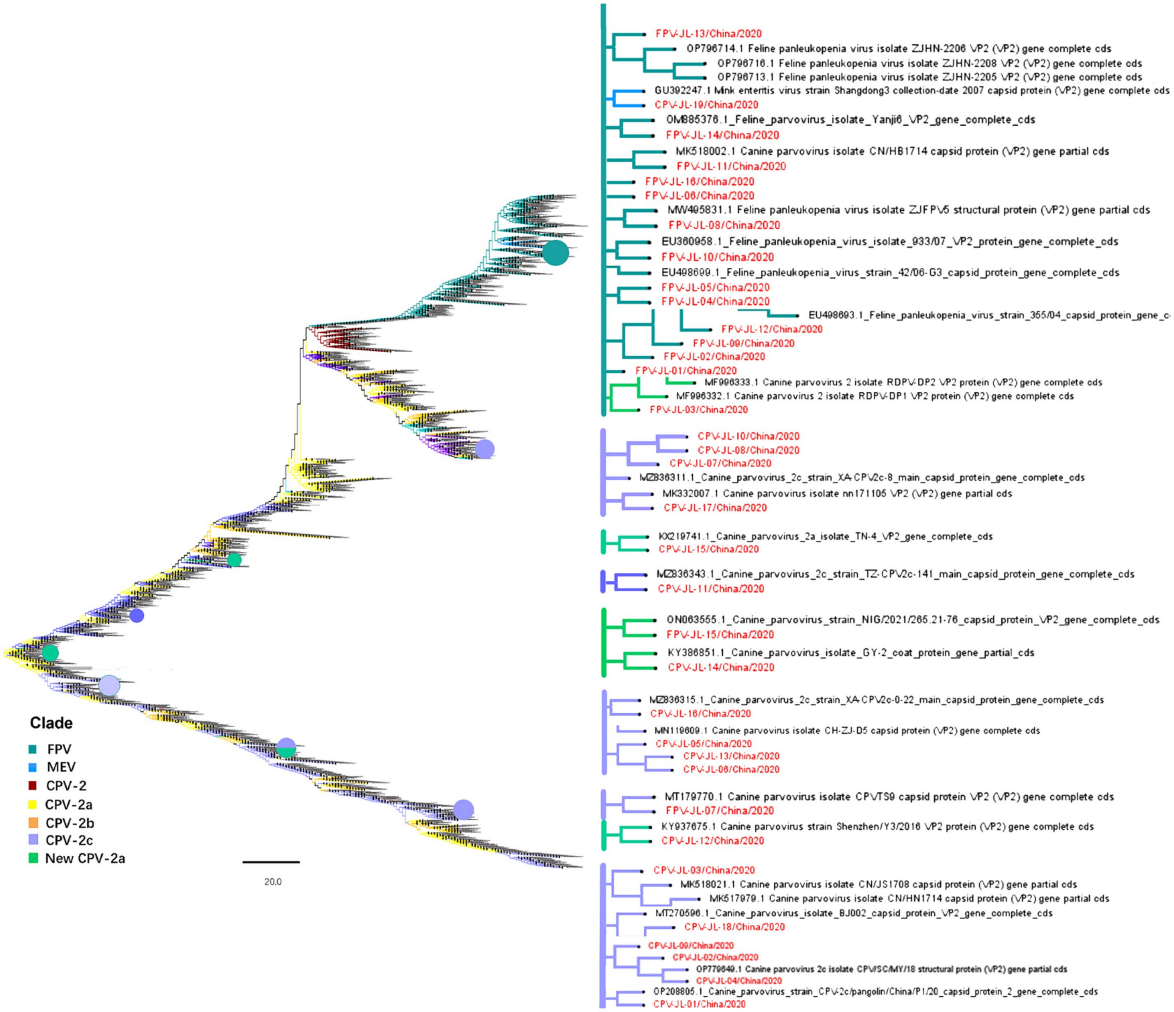
Neighbor-joining phylogenetic tree based on the VP2 genes. The VP2 gene (1755 bp) originated from this study samples and the other 2,181 referenced VP2 genes collected from the NCBI GenBank database were used to analyze genetic evolutionary relationships. The viral genes obtained in this study are in red.

In this study, 11 NS1 genes were obtained from CPV-2 variants and 17 NS1 genes from FPV. Sequences of the obtained NS1 genes were truncated to 2007 bp before alignment, which encoded NS1 protein with 669 aa residues. The aa alignment based on 129 published NS1 sequences (25 FPV and 104 CPV-2) and 28 isolated in this study revealed unique aa mutations of N23D, Q247H, I248T, Tyr544Phe, E545V, and L630P (Figure 1; Table 3). Sites Q247 H, 248 I to T, 544 Y to F, and 545 Q to V could distinguish CPV-2 from FPV. Considering the limited number of NS1 genes published in GenBank, the genotype based on NS1 genes remained unclear. All the NS1 gene sequences shared 99.51% identity. Phylogenetic analysis of NS1 genes showed that the CPV-JL-15/China/2020 clustered with FPV (Figure 2), and its VP2 genotype belonged to the new CPV-2a (Figure 3). Consistently, FPV-JL-07/China/2020 clustered to CPV-2 based on genetic relationships of NS1 genes (Figure 2), and its VP2 genotype belonged to the CPV-2c (Figure 3). CPV-JL-15/China/2020 had N23D mutation in NS1 protein, and FPV-JL-07/China/2020 had L630P in NS1 protein. Combined with the above phylogenetic results of NS1 genes, N23D or L630P mutation in NS1 protein could help as a reminder of genetic relationships of CPV-2 and FPV in the same branch.

### 3.3 Exaggerated variant, epidemic, and genetic recombination of CPV-2 and FPV

Phylogenetic analyses of 2,181 VP2 genes with 1755 bp in length from the GenBank database (in total with a final landing date of 5 April 2023) and 35 isolated VP2 genes (19 CPV-2 and 16 FPV) showed that: (i) the CPV-2c and CPV-2a subtypes were currently predominant circulating canine parvoviruses worldwide (Figure 3); (ii) the original CPV-2 type has been replaced by various CPV-2 subtypes around the world; (iii) CPV-2 lineage and FPV lineage formed two independent branches and MEV lineage mixed with those of FPV. In this study, FPV-JL-1/China/2020, FPV-JL-2/China/2020, FPV-JL-4/China/2020, FPV-JL-5/China/2020, FPV-JL-9/China/2020, FPV-JL-10/China/2020, FPV-JL-12/China/2020, FPV-JL-15/China/2020, and CPV-JL-15/China/2020 were highly related to strains in Italy, India, and Hungary, in which FPV-JL-1/China/2020, FPV-JL-2/China/2020, FPV-JL-4/China/2020, FPV-JL-5/China/2020, FPV-JL-9/China/2020, FPV-JL-12/China/2020, and FPV-JL-15/China/2020 were closely related to strains in Italy. The other 26 strains were highly related to domestic strains, in which 7 strains were close to strain in Henan (FPV-JL-06/China/2020, FPV-JL-11/China/2020, FPV-JL-16/China/2020, CPV-JL-03/China/2020, CPV-JL-05/China/2020, CPV-JL-06/China/2020, and CPV-JL-13/China/2020); 7 strains (CPV-JL-07/China/2020, CPV-JL-08/China/2020, CPV-JL-10/China/2020, CPV-JL-11/China/2020, CPV-JL-16/China/2020, CPV-JL-18/China/2020, and FPV-JL-07/China/2020) were close to Beijing strain, and 12 strain were close to strain in Jilin, Jiangsu, Shandong, Guizhou, Sichuan, and Guangxi, in China. These findings indicated parvoviruses with great ability to cross-region transmissions, which could be caused by cold chain transports or pet trades to China.

The VP2 gene of FPV-JL-10/China/2020 was closely related to a parvovirus that infected an Asian palm civet in 2009 (No. EU360958.1) with a 99.72% homology rate, indicating strong survival and transmission abilities. Another interesting finding was that FPV-JL-15/China/2020 from cats was 99.89% close to ON063555.1 detected in domestic dogs in Nigeria in 2021, which indicated the continuous transmission of CPV-2a strains across species and regions. In addition, the CPV-JL-19/China/2020 was closely related to the MEV isolated from mink in Jilin Province in 2009 with no gene recombination by RDP, GENECONV, BootScan, MaxChi, Chimaera, SiScan, and 3seq.

A recombination event in the VP2 gene of the CPV-JL-06/China/2020 strain was identified using RDP4 software. These findings were further validated through phylogenetic analysis. The major parent strain of CPV-JL-06/China/2020 is M19296 from the United States, with EU310373 from Jilin, China, as the minor parent strain (Supplementary Figure S1). For CPV-JL-7, the major parent strain is KP749870 from Beijing, China, while the minor parent strain is MZ836379 from Jilin, China (Supplementary Figure S2). The *p*-values obtained from the recombination analysis were all below 0.05.

## 4 Discussion

Currently, CPV-2 variants infect both canines and felines around the world, while FPV is still the dominant virus in felids (40, 41). FPV was first identified in 1928 and later isolated from tissue culture in 1964 (42). CPV-2 was originally found in 1978 and spread worldwide within months (43). FPV is determined by the aa residues in VP2 protein at sites of 80 K, 93 K, 103 V, 323D, 564 N, and 568A, whereas CPV-2 is identified by 297S, 300A, 305D, 323 N, and 426 N (39). Between 1979 and 1981, CPV-2a (297S, 426 N) was discovered as an antigenic variant. In 1984, a second variant, CPV-2b (297S, 426D), was identified (44, 45). In 1990, the S297A mutation appeared in VP2 of CPV-2a and CPV-2b, which were designated as new CPV-2a (297A, 426 N) and new CPV-2b (297A, 426D) accordingly (14). The CPV-2c (297A, 426E) antigenic variant was first reported in Italy in 2000 (15). Currently, accumulating epidemiological evidence has revealed that CPV-2c has become a new dominant variant in Asia, South America, North America, and Africa (21, 46–48). It was consistent with our findings of an epidemiological survey for canine and feline parvoviruses in Changchun City in Northeast China in 2020 that 31 out of 39 CPV-2 variants (79.5%) were identified as CPV-2c infection in dogs. In addition, CPV-2c and new CPV-2a strains were also found in cats, which was consistent with previous research that all current CPV-2 variants (CPV-2a-c) can infect cats to cause subclinical disease or feline panleukopenia (18).

The VP2 protein was the main component of the viral envelope, determining host range, antigenicity, and antigenic drift (48). The VP2 protein had five important domains: Loop1, Loop2, Loop3, Loop4, and Loop5, with representative aa residues of 50–100, 200–250, 300–350, 400–450, and 250–400, respectively (49). The 297 aa residue was located at Loop3, which not only formed the shoulder of the triple folding but also participated in the formation of the antigen site B (50). In our results, three strains (CPV-JL-07/China/2020, CPV-JL-10/China/2020, and CPV-JL-12/China/2020) in dogs showed A297L mutation, two strains (CPV-JL-01/China/2020 and CPV-JL-19/China/2020) showed A297S mutation, and FPV-JL-04/China/2020 in cats showed K80R mutation. The 80 aa of VP2 protein was situated in Loop 1, which formed antigen site A, and its mutations influenced the recognition of neutralizing antibodies against antigen site A. In previous studies, scholars argued that the Gln370Arg mutation was specific for all identified CPV-2c strains, and all new CPV-2a strains showed the Thr440A mutation (51). However, the new CPV-2a FPV-JL-03/China/2020 strain from cats was 440 T and did not mutate to A. In recent research, the A5G and Q370R mutations in VP2 protein were the most common changes in newly isolated CPV-2c strains (38). The CPV-2c strains found in the present study were mostly consistent with those conclusions, but CPV-JL-14/China/2020 and CPV-JL-15/China/2020 were 5A. A previous study showed that the 5 aa residue was on the viral surface as a core residue in the antigenic sites (52). Therefore, this mutation of A5G might change viral antigenicity and immunogenicity. The F267Y, Y324I, and Q370R mutations were located in the greatest variable GH loop (loops 3 and 4) comprising aa 267–498 in VP2 protein (47). The isolated FPV-JL-15/China/2020 from cats in 2020 had a close 99.89% with the published ON063555.1 in domestic dogs in Nigeria in 2021, which were only different at A809T and A1187G. Whether these two aa sites were key mutations in viral transmission from cats to dogs would be a new direction. Interestingly, the genotype of CPV-JL-19/China/2020 was initially considered to be CPV-2a. CPV-JL-19/China/2020 did not align with any of the CPV-2 reference typing sites. However, in the VP2 phylogenetic analysis, CPV-JL-19/China/2020 clustered with MEV, and subsequent comparison with MEV reference sequences confirmed that CPV-JL-19/China/2020 is indeed MEV. Later, we discovered that it is closely related to an MEV strain isolated from mink in Shandong Province, China, in 2007, with 99.32% homology to the Shangdong3 strain (GU392247). This was a case that MEV strain infected dogs, but it was unlike the MEV strain with E75K, Q159H, D178H, and Q242H mutations by comparing with 41 VP2 gene sequences of MEV. We suspect that these mutations enabled MEV to infect dogs.

Viral recombination could lead to the emergence of new virus strains that cause various infections and pathogenicity in different hosts, which evolve into new epidemics. A recombination event between MEV and CPV-2 occurred in 2012, in which the NS1 gene originated from CPV-2 while the VP1 gene came from MEV (27). No recombination events were detected based on the recombination analysis of the VP2 gene in CPV-JL-19/China/2020. Thus, it needs further whole-genome recombination analysis to confirm recombination in CPV-JL-19/China/2020. A recombination event was found in the CPV-JL-06/China/2020 strain with the breakpoint precisely located at 920 nucleotides in the VP2 gene by RDP software, applying seven programs: RDP, GENECONV, BootScan, MaxChi, Chimaera, SiScan, and 3Seq. Phylogenetic analyses further validated this finding that CPV-JL-06/China/2020 clustered with the minor parent CPV-2a strain (No. EU310373) discovered in China in 2008 in the phylogenetic tree of nucleotides 1–920, while the major parent CPV-2 strain (No. M19296) discovered in the United States in 1995 formed a separate branch. Conversely, CPV-JL-06/China/2020 clustered with the major parent M19296 strain, and EU310373 was located on a different branch in the phylogenetic tree of nucleotides 920–1755. These results again confirmed that CPV-2 variants were easy to evolve through genetic recombination.

Most studies of genetic evolution and pathogenic molecular mechanisms of carnivore protoparvovirus 1 had focused on its VP2 gene (53, 54), but the potential critical roles of the NS1 gene were not well characterized. In this study, the alignment of 129 published NS1 genes and 28 isolate NS1 genes led to the preliminary identification of six aa sites (N23D, Q247H, I248T, Y544F, E545V, and P630L) that can distinguish between CPV-2 and FPV. Four out of six sites had been reported in previous studies. It had been recognized that 247Gln and 595Gln in NS1 protein were specific features of CPV-2 (2). In a 2019 study, it was shown that the aa mutation at site 248 had been used as a biomarker to clearly and constantly distinguish FPV from CPV-2 and amino acids at specific residues (247, 545, and 595), previously potentially designated as discriminating the viral types (2). The 544F mutation had been observed in older sequences from the United States (1983–2010) and New Zealand (1994), as well as in more recent sequences from South America, Canada, and China (2), 630P was reported as a synonymous mutation in the NS1 protein in Asian CPV-2 strains (55). The phylogenetic analysis of NS1 genes reveals that the NS1 gene distinctly clusters into two main groups: FPV and CPV-2 (Figure 2). Based on the aa alignment results, we found that site 23 was unique in CPV-2 (CPV-JL-6), with a D/N ratio of 1/116, whereas in FPV, the D/N ratio is 42/19. Typically, 23 N in the NS1 gene is always CPV-2, but when the N23D mutation appeared, the CPV-2 was classified as FPV lineage based on phylogenetic analysis of the NS1 gene. In addition, site 630P is unique in FPV (FPV-JL-4), with a P/L ratio of 1/42, while the P/L ratio is 42/116 in CPV-2. Generally, site 630 L in the NS1 gene is FPV, but when this site is P, the NS1 gene is classified as CPV-2.

In previous studies, some scholars described the function of the NS1 protein (56, 57) and indicated the potential location of the functional domain, the origin of replication (ORI) binding (16-275aa), helicase (299-486aa), and transactivation (600–667aa) functional domains (4, 58). We hypothesized that the difference between 23D and 630P might affect viral replication and gene transcription. In addition, recent scholars have proposed changes in sites M11K, I60V, N351K, N361S, Y544F, E545V, and E572K (38). We also analyzed the sequences in this study and found that I60V, Y544F, and E545V mutations accounted for 32.1% (9/28), 35.7% (10/28), and 28.6% (8/28), respectively, and all Glu545Val mutations were found in CPV-2. No changes in M11K, N351K, and N361S were found. A recent NS1 study investigated the phosphorylation sites of 598 T and 601 T to the C terminus, and these two sites are essential for viral replication and pathogenicity (55), 630 on the transcriptional activation functional domain (59) The migration of CPV-2 is likely facilitated by the movement of infected animals or mechanical vectors among countries in close geographic proximity (60). This may explain why the strains in this study are closely related to those found in Italy, Hungary, and other countries.

## 5 Conclusion

An epidemiological survey of canine and feline parvoviruses in Changchun City in Northeast China in 2020 revealed that 64.3% of dogs and 55.7% of cats with gastrointestinal clinical signs were CPV-2 or FPV-positive by specific antigen colloidal gold strip tests. Sequencing analysis of VP2 genes demonstrated that 79.5% of CPV-2c and 17.9% of new CPV-2a were the dominant variants in CPV-2 antigen-positive dogs and 81.25% of FPV-infected cats in Changchun. In addition, a genetic recombination event was observed in CPV-JL-06/China/2020 and CPV-JL-7. Phylogenetic analysis of the NS1 gene, combined with aa alignment results, identified two novel sites, 23D and 630P, which for the first time may help distinguish whether the NS1 gene is classified as CPV-2 or FPV. These findings provided new insights into the evolution of CPV-2 variants and FPV, highlighting the great necessity of ongoing surveillance of parvovirus to block its spread.

## Data Availability

The datasets presented in this study can be found in online repositories. The names of the repository/repositories and accession number(s) can be found in the article/Supplementary material.

## Supplementary material

Supplementary Figure S1 Recombinant analysis of CPV-JL-06/China/2020 A: Results of RDP recombination analysis of CPV-JL-06/China/2020 isolate; B: Phylogenetic analysis of CPV-2 VP2 isolates based on the nt1-920; C: Phylogenetic analysis of CPV-2 VP2 isolates based on the nt920-1755.

Supplementary Figure S2 Results of CPV-JL-7 recombination analysis and *p*-value results.
