# Supplementary material for: Two novel sites determine genetic relationships between CPV-2 and FPV: an epidemiological survey of canine and feline parvoviruses in Changchun, China (2020)
Source: Front Vet Sci. 2024 Nov 4;11:1444984. doi: 10.3389/fvets.2024.1444984 (PMC11571754; doi:10.3389/fvets.2024.1444984)
Supplement: Supplementary file 1 [file Table_1.docx]

Table S1 Basic information on the sequences used for the phylogenetic analysis of the VP2 gene

| **ACCESSION** | **Country** | **Source** |
| --- | --- | --- |
| >MK332007.1 | Nanning. China | Canine parvovirus |
| >MK332006.1 | Nanning. China | Canine parvovirus |
| >MK332005.1 | Nanning. China | Canine parvovirus |
| >MK332004.1 | Nanning. China | Canine parvovirus |
| >MK332003.1 | Nanning. China | Canine parvovirus |
| >MK332002.1 | Nanning. China | Canine parvovirus |
| >MK332001.1 | Nanning. China | Canine parvovirus |
| >MK332000.1 | Nanning. China | Canine parvovirus |
| >MK331999.1 | Nanning. China | Canine parvovirus |
| >MK331998.1 | Nanning. China | Canine parvovirus |
| >MK331997.1 | Nanning. China | Canine parvovirus |
| >MK331996.1 | Nanning. China | Canine parvovirus |
| >KJ674820.1 | China | Canine parvovirus |
| >KJ674819.1 | China | Canine parvovirus |
| >KJ674818.1 | China | Canine parvovirus |
| >KJ674817.1 | China | Canine parvovirus |
| >KJ674816.1 | China | Canine parvovirus |
| >KJ674815.1 | China | Canine parvovirus |
| >KJ674814.1 | China | Canine parvovirus |
| >KJ674813.1 | China | Canine parvovirus |
| >KJ674812.1 | China | Canine parvovirus |
| >KJ674811.1 | China | Canine parvovirus |
| >KJ674810.1 | China | Canine parvovirus |
| >KJ674809.1 | China | Canine parvovirus |
| >KJ674808.1 | China | Canine parvovirus |
| >KJ674807.1 | China | Canine parvovirus |
| >KJ674806.1 | China | Canine parvovirus |
| >AB128923.1 | Japan | Canine parvovirus |
| >MW648369.1 | Brazil | Canine parvovirus |
| >MW648368.1 | Brazil | Canine parvovirus |
| >MW648367.1 | Brazil | Canine parvovirus |
| >MW648366.1 | Brazil | Canine parvovirus |
| >MW648365.1 | Brazil | Canine parvovirus |
| >MW648364.1 | Brazil | Canine parvovirus |
| >MW648363.1 | Brazil | Canine parvovirus |
| >MW648362.1 | Brazil | Canine parvovirus |
| >MW648361.1 | Brazil | Canine parvovirus |
| >MW648360.1 | Brazil | Canine parvovirus |
| >MW648359.1 | Brazil | Canine parvovirus |
| >MW648358.1 | Brazil | Canine parvovirus |
| >MW648357.1 | Brazil | Canine parvovirus |
| >MW648356.1 | Brazil | Canine parvovirus |
| >MW648355.1 | Brazil | Canine parvovirus |
| >MW648354.1 | Brazil | Canine parvovirus |
| >MW648353.1 | Brazil | Canine parvovirus |
| >MW648352.1 | Brazil | Canine parvovirus |
| >MW648351.1 | Brazil | Canine parvovirus |
| >MW648350.1 | Brazil | Canine parvovirus |
| >MW648349.1 | Brazil | Canine parvovirus |
| >MW648348.1 | Brazil | Canine parvovirus |
| >MN101726.1 | Beijing. China | Canine parvovirus |
| >MN101725.1 | Beijing. China | Canine parvovirus |
| >MN101724.1 | Beijing. China | Canine parvovirus |
| >KJ813892.1 | NY. USA | Canine parvovirus |
| >KJ813891.1 | NY. USA | Canine parvovirus |
| >KJ813890.1 | NY. USA | Canine parvovirus |
| >KJ813889.1 | NY. USA | Canine parvovirus |
| >KJ813888.1 | NY. USA | Canine parvovirus |
| >KJ813887.1 | NY. USA | Canine parvovirus |
| >KJ813886.1 | NY. USA | Canine parvovirus |
| >KJ813885.1 | NY. USA | Canine parvovirus |
| >KJ813884.1 | NY. USA | Canine parvovirus |
| >KJ813883.1 | NY. USA | Canine parvovirus |
| >KJ813882.1 | NY. USA | Canine parvovirus |
| >KJ813881.1 | NY. USA | Canine parvovirus |
| >KJ813880.1 | NY. USA | Canine parvovirus |
| >KJ813879.1 | NY. USA | Canine parvovirus |
| >KJ813878.1 | NY. USA | Canine parvovirus |
| >KJ813877.1 | NY. USA | Canine parvovirus |
| >KJ813876.1 | NY. USA | Canine parvovirus |
| >KJ813875.1 | NY. USA | Canine parvovirus |
| >KJ813874.1 | NY. USA | Canine parvovirus |
| >KJ813873.1 | NY. USA | Canine parvovirus |
| >KJ813872.1 | NY. USA | Canine parvovirus |
| >KJ813871.1 | NY. USA | Canine parvovirus |
| >KJ813870.1 | NY. USA | Canine parvovirus |
| >KJ813869.1 | NY. USA | Canine parvovirus |
| >KJ813868.1 | NY. USA | Canine parvovirus |
| >KJ813867.1 | NY. USA | Canine parvovirus |
| >KJ813866.1 | NY. USA | Canine parvovirus |
| >KJ813865.1 | NY. USA | Canine parvovirus |
| >KJ813864.1 | NY. USA | Canine parvovirus |
| >KJ813863.1 | NY. USA | Canine parvovirus |
| >KJ813862.1 | NY. USA | Canine parvovirus |
| >KJ813861.1 | NY. USA | Canine parvovirus |
| >KJ813860.1 | NY. USA | Canine parvovirus |
| >KJ813859.1 | NY. USA | Canine parvovirus |
| >KJ813858.1 | NY. USA | Canine parvovirus |
| >KJ813857.1 | NY. USA | Canine parvovirus |
| >KJ813856.1 | NY. USA | Canine parvovirus |
| >KJ813855.1 | NY. USA | Canine parvovirus |
| >KJ813854.1 | NY. USA | Canine parvovirus |
| >KJ813853.1 | NY. USA | Canine parvovirus |
| >KJ813852.1 | NY. USA | Canine parvovirus |
| >KJ813851.1 | NY. USA | Canine parvovirus |
| >KJ813850.1 | NY. USA | Canine parvovirus |
| >KJ813849.1 | NY. USA | Canine parvovirus |
| >KJ813848.1 | NY. USA | Canine parvovirus |
| >KJ813847.1 | NY. USA | Canine parvovirus |
| >KJ813846.1 | NY. USA | Canine parvovirus |
| >KJ813845.1 | NY. USA | Canine parvovirus |
| >KJ813844.1 | NY. USA | Canine parvovirus |
| >KJ813843.1 | NY. USA | Canine parvovirus |
| >KJ813842.1 | NY. USA | Canine parvovirus |
| >KJ813841.1 | NY. USA | Canine parvovirus |
| >KJ813840.1 | NY. USA | Canine parvovirus |
| >KJ813839.1 | NY. USA | Canine parvovirus |
| >KJ813838.1 | NY. USA | Canine parvovirus |
| >KJ813837.1 | NY. USA | Canine parvovirus |
| >KJ813836.1 | NY. USA | Canine parvovirus |
| >KJ813835.1 | NY. USA | Canine parvovirus |
| >KJ813834.1 | NY. USA | Canine parvovirus |
| >KJ813833.1 | NY. USA | Canine parvovirus |
| >KJ813832.1 | NY. USA | Canine parvovirus |
| >KJ813831.1 | NY. USA | Canine parvovirus |
| >KJ813830.1 | NY. USA | Canine parvovirus |
| >KJ813829.1 | NY. USA | Canine parvovirus |
| >KJ813828.1 | NY. USA | Canine parvovirus |
| >KJ813827.1 | NY. USA | Canine parvovirus |
| >KF149985.1 | Uruguay | Canine parvovirus |
| >KF149984.1 | Uruguay | Canine parvovirus |
| >KF149983.1 | Uruguay | Canine parvovirus |
| >KF149982.1 | Uruguay | Canine parvovirus |
| >KF149981.1 | Uruguay | Canine parvovirus |
| >KF149980.1 | Uruguay | Canine parvovirus |
| >KF149979.1 | Uruguay | Canine parvovirus |
| >KF149978.1 | Uruguay | Canine parvovirus |
| >KF149977.1 | Uruguay | Canine parvovirus |
| >KF149976.1 | Uruguay | Canine parvovirus |
| >KF149975.1 | Uruguay | Canine parvovirus |
| >KF149974.1 | Uruguay | Canine parvovirus |
| >KF149973.1 | Uruguay | Canine parvovirus |
| >KF149972.1 | Uruguay | Canine parvovirus |
| >KF149971.1 | Uruguay | Canine parvovirus |
| >KF149970.1 | Uruguay | Canine parvovirus |
| >KF149969.1 | Uruguay | Canine parvovirus |
| >KF149968.1 | Uruguay | Canine parvovirus |
| >KF149967.1 | Uruguay | Canine parvovirus |
| >KF149966.1 | Uruguay | Canine parvovirus |
| >KF149965.1 | Uruguay | Canine parvovirus |
| >KF149964.1 | Uruguay | Canine parvovirus |
| >KF149963.1 | Uruguay | Canine parvovirus |
| >KF149962.1 | Uruguay | Canine parvovirus |
| >FJ197847.1 | South Korea | Canine parvovirus 2 |
| >FJ197846.1 | South Korea | Canine parvovirus 2 |
| >FJ197845.1 | South Korea | Canine parvovirus 2 |
| >FJ197844.1 | South Korea | Canine parvovirus 2 |
| >FJ197843.1 | South Korea | Canine parvovirus 2 |
| >FJ197842.1 | South Korea | Canine parvovirus 2 |
| >FJ197841.1 | South Korea | Canine parvovirus 2 |
| >FJ197840.1 | South Korea | Canine parvovirus 2 |
| >FJ197839.1 | South Korea | Canine parvovirus 2 |
| >FJ197838.1 | South Korea | Canine parvovirus 2 |
| >FJ197837.1 | South Korea | Canine parvovirus 2 |
| >FJ197836.1 | South Korea | Canine parvovirus 2 |
| >FJ197835.1 | South Korea | Canine parvovirus 2 |
| >FJ197834.1 | South Korea | Canine parvovirus 2 |
| >FJ197833.1 | South Korea | Canine parvovirus 2 |
| >FJ197832.1 | South Korea | Canine parvovirus 2 |
| >FJ197831.1 | South Korea | Canine parvovirus 2 |
| >FJ197830.1 | South Korea | Canine parvovirus 2 |
| >FJ197829.1 | South Korea | Canine parvovirus 2 |
| >FJ197828.1 | South Korea | Canine parvovirus 2 |
| >FJ197827.1 | South Korea | Canine parvovirus 2 |
| >FJ197826.1 | South Korea | Canine parvovirus 2 |
| >FJ197825.1 | South Korea | Canine parvovirus 2 |
| >FJ197824.1 | South Korea | Canine parvovirus 2 |
| >FJ197823.1 | South Korea | Canine parvovirus 2 |
| >DQ026002.1 | France | Canine parvovirus |
| >DQ026001.1 | France | Canine parvovirus |
| >DQ026000.1 | France | Canine parvovirus |
| >DQ025999.1 | France | Canine parvovirus |
| >DQ025998.1 | France | Canine parvovirus |
| >DQ025997.1 | France | Canine parvovirus |
| >DQ025996.1 | France | Canine parvovirus |
| >DQ025995.1 | France | Canine parvovirus |
| >DQ025994.1 | France | Canine parvovirus |
| >DQ025993.1 | France | Canine parvovirus |
| >DQ025992.1 | France | Canine parvovirus |
| >DQ025991.1 | France | Canine parvovirus |
| >DQ025990.1 | France | Canine parvovirus |
| >DQ025989.1 | France | Canine parvovirus |
| >DQ025988.1 | France | Canine parvovirus |
| >DQ025987.1 | France | Canine parvovirus |
| >DQ025986.1 | France | Canine parvovirus |
| >DQ025985.1 | France | Canine parvovirus |
| >DQ025984.1 | France | Canine parvovirus |
| >DQ025983.1 | France | Canine parvovirus |
| >DQ025982.1 | France | Canine parvovirus |
| >DQ025981.1 | France | Canine parvovirus |
| >DQ025980.1 | France | Canine parvovirus |
| >DQ025979.1 | France | Canine parvovirus |
| >DQ025978.1 | France | Canine parvovirus |
| >DQ025977.1 | France | Canine parvovirus |
| >DQ025976.1 | France | Canine parvovirus |
| >DQ025975.1 | France | Canine parvovirus |
| >DQ025974.1 | France | Canine parvovirus |
| >DQ025973.1 | France | Canine parvovirus |
| >DQ025972.1 | France | Canine parvovirus |
| >DQ025971.1 | France | Canine parvovirus |
| >DQ025970.1 | France | Canine parvovirus |
| >DQ025969.1 | France | Canine parvovirus |
| >DQ025968.1 | France | Canine parvovirus |
| >DQ025967.1 | France | Canine parvovirus |
| >DQ025966.1 | France | Canine parvovirus |
| >DQ025965.1 | France | Canine parvovirus |
| >DQ025964.1 | France | Canine parvovirus |
| >DQ025963.1 | France | Canine parvovirus |
| >DQ025962.1 | France | Canine parvovirus |
| >DQ025961.1 | France | Canine parvovirus |
| >DQ025960.1 | France | Canine parvovirus |
| >DQ025959.1 | France | Canine parvovirus |
| >DQ025958.1 | France | Canine parvovirus |
| >DQ025957.1 | France | Canine parvovirus |
| >DQ025956.1 | France | Canine parvovirus |
| >DQ025955.1 | France | Canine parvovirus |
| >DQ025954.1 | France | Canine parvovirus |
| >DQ025953.1 | France | Canine parvovirus |
| >DQ025952.1 | France | Canine parvovirus |
| >DQ025951.1 | France | Canine parvovirus |
| >DQ025950.1 | France | Canine parvovirus |
| >DQ025949.1 | France | Canine parvovirus |
| >DQ025948.1 | France | Canine parvovirus |
| >DQ025947.1 | France | Canine parvovirus |
| >DQ025946.1 | France | Canine parvovirus |
| >DQ025945.1 | France | Canine parvovirus |
| >DQ025944.1 | France | Canine parvovirus |
| >DQ025943.1 | France | Canine parvovirus |
| >DQ025942.1 | France | Canine parvovirus |
| >OP796705.1 | Nanjing. Jiangsu. China | Canine parvovirus |
| >OP796704.1 | Nanjing. Jiangsu. China | Canine parvovirus |
| >OP796703.1 | Nanjing. Jiangsu. China | Canine parvovirus |
| >OP796702.1 | Nanjing. Jiangsu. China | Canine parvovirus |
| >OP796701.1 | Nanjing. Jiangsu. China | Canine parvovirus |
| >OP796700.1 | Nanjing. Jiangsu. China | Canine parvovirus |
| >OP796699.1 | Nanjing. Jiangsu. China | Canine parvovirus |
| >MW815498.1 | Beijing. China | Canine parvovirus |
| >MW815497.1 | Beijing. China | Canine parvovirus |
| >MW679576.1 | Brazil | Canine parvovirus |
| >MW679575.1 | Brazil | Canine parvovirus |
| >MW679574.1 | Brazil | Canine parvovirus |
| >MW679573.1 | Brazil | Canine parvovirus |
| >MW679572.1 | Brazil | Canine parvovirus |
| >MW679571.1 | Brazil | Canine parvovirus |
| >MW679570.1 | Brazil | Canine parvovirus |
| >MW679569.1 | Brazil | Canine parvovirus |
| >LC570804.1 | Japan | Canine parvovirus |
| >MH660525.1 | Nanjing. Jiangsu. China | Canine parvovirus |
| >MH660524.1 | Nanjing. Jiangsu. China | Canine parvovirus |
| >MH660523.1 | Nanjing. Jiangsu. China | Canine parvovirus |
| >AB120728.1 | Tokyo. Japan | Canine parvovirus |
| >AB120727.1 | Tokyo. Japan | Canine parvovirus |
| >AB120726.1 | Tokyo. Japan | Canine parvovirus |
| >AB120725.1 | Tokyo. Japan | Canine parvovirus |
| >AB120724.1 | Tokyo. Japan | Canine parvovirus |
| >AB120723.1 | Tokyo. Japan | Canine parvovirus |
| >AB120722.1 | Tokyo. Japan | Canine parvovirus |
| >AB120721.1 | Tokyo. Japan | Canine parvovirus |
| >AB120720.1 | Tokyo. Japan | Canine parvovirus |
| >AB115504.1 | Tokyo. Japan | Canine parvovirus |
| >AB054222.2 | Tokyo. Japan | Canine parvovirus |
| >AB054224.1 | Tokyo. Japan | Canine parvovirus |
| >AB054223.1 | Tokyo. Japan | Canine parvovirus |
| >AB054221.1 | Tokyo. Japan | Canine parvovirus |
| >AB054220.1 | Tokyo. Japan | Canine parvovirus |
| >AB054219.1 | Tokyo. Japan | Canine parvovirus |
| >AB054218.1 | Tokyo. Japan | Canine parvovirus |
| >AB054217.1 | Tokyo. Japan | Canine parvovirus |
| >AB054216.1 | Tokyo. Japan | Canine parvovirus |
| >AB054215.1 | Tokyo. Japan | Canine parvovirus |
| >AB054214.1 | Tokyo. Japan | Canine parvovirus |
| >AB054213.1 | Tokyo. Japan | Canine parvovirus |
| >KM624023.1 | NY.USA | Canine parvovirus |
| >KJ813895.1 | NY.USA | Feline panleukopenia virus |
| >KJ813894.1 | NY.USA | Feline panleukopenia virus |
| >KJ813893.1 | NY.USA | Feline panleukopenia virus |
| >OP796716.1 | Nanjing. Jiangsu. China | Feline panleukopenia virus |
| >OP796715.1 | Nanjing. Jiangsu. China | Feline panleukopenia virus |
| >OP796714.1 | Nanjing. Jiangsu. China | Feline panleukopenia virus |
| >OP796713.1 | Nanjing. Jiangsu. China | Feline panleukopenia virus |
| >OP796712.1 | Nanjing. Jiangsu. China | Feline panleukopenia virus |
| >OP796711.1 | Nanjing. Jiangsu. China | Feline panleukopenia virus |
| >OP796710.1 | Nanjing. Jiangsu. China | Feline panleukopenia virus |
| >OP796709.1 | Nanjing. Jiangsu. China | Feline panleukopenia virus |
| >OP796708.1 | Nanjing. Jiangsu. China | Feline panleukopenia virus |
| >OP796707.1 | Nanjing. Jiangsu. China | Feline panleukopenia virus |
| >OP796706.1 | Nanjing. Jiangsu. China | Feline panleukopenia virus |
| >OM892824.1 | Romagna. Italia | Protoparvovirus carnivoran1 |
| >OM892823.1 | Romagna. Italia | Protoparvovirus carnivoran1 |
| >MW091486.1 | Changchun. Jilin. China | Feline panleukopenia virus |
| >KY094119.1 | Shandong. China | Mink enteritis virus |
| >KY094118.1 | Shandong. China | Mink enteritis virus |
| >KY094117.1 | Shandong. China | Mink enteritis virus |
| >KY094116.1 | Shandong. China | Mink enteritis virus |
| >KY094115.1 | Shandong. China | Mink enteritis virus |
| >KY094114.1 | Shandong. China | Mink enteritis virus |
| >KY094113.1 | Shandong. China | Mink enteritis virus |
| >KY094112.1 | Shandong. China | Mink enteritis virus |
| >FJ405225.1 | Changchun. Jilin. China | Feline panleukopenia virus |
| >AB054227.1 | Tokyo. Japan | Feline panleukopenia virus |
| >AB054226.1 | Tokyo. Japan | Feline panleukopenia virus |
| >AB054225.1 | Tokyo. Japan | Feline panleukopenia virus |
| >KX421789.1 | Taipei. Taiwan | Canine parvovirus 2 |
| >KX421788.1 | Taipei. Taiwan | Canine parvovirus 2 |
| >KX421787.1 | Taipei. Taiwan | Canine parvovirus 2 |
| >KX421786.1 | Taipei. Taiwan | Canine parvovirus 2 |
| >MT880781.1 | Uttar Pradesh. India | Canine parvovirus |
| >MT585713.1 | Santiago. Chile | Canine parvovirus 2b |
| >MT585712.1 | Santiago. Chile | Canine parvovirus 2b |
| >MT585711.1 | Santiago. Chile | Canine parvovirus 2c |
| >MT585710.1 | Santiago. Chile | Canine parvovirus 2c |
| >MT585709.1 | Santiago. Chile | Canine parvovirus 2c |
| >MT585708.1 | Santiago. Chile | Canine parvovirus 2c |
| >MT585707.1 | Santiago. Chile | Canine parvovirus 2c |
| >MT585706.1 | Santiago. Chile | Canine parvovirus 2c |
| >MT585705.1 | Santiago. Chile | Canine parvovirus 2c |
| >MT585704.1 | Santiago. Chile | Canine parvovirus 2c |
| >MT585703.1 | Santiago. Chile | Canine parvovirus 2c |
| >MT585702.1 | Santiago. Chile | Canine parvovirus 2c |
| >MT585701.1 | Santiago. Chile | Canine parvovirus 2c |
| >MT585700.1 | Santiago. Chile | Canine parvovirus 2c |
| >MT585699.1 | Santiago. Chile | Canine parvovirus 2c |
| >MT585698.1 | Santiago. Chile | Canine parvovirus 2c |
| >MT585697.1 | Santiago. Chile | Canine parvovirus 2c |
| >MT585696.1 | Santiago. Chile | Canine parvovirus 2c |
| >MN473467.1 | Yinchuan. Ningxia. China | Canine parvovirus |
| >MN473466.1 | Yinchuan. Ningxia. China | Canine parvovirus |
| >MN473465.1 | Yinchuan. Ningxia. China | Canine parvovirus |
| >EU095252.1 | Changchun. Jilin. China | Canine parvovirus |
| >KX878988.1 | Uttar Pradesh. India | Canine parvovirus 2 |
| >KX469435.1 | Uttar Pradesh. India | Canine parvovirus 2a |
| >KX469434.1 | Uttar Pradesh. India | Canine parvovirus 2a |
| >KX469433.1 | Uttar Pradesh. India | Canine parvovirus 2a |
| >KX469432.1 | Uttar Pradesh. India | Canine parvovirus 2b |
| >KX469431.1 | Uttar Pradesh. India | Canine parvovirus 2a |
| >KX469430.1 | Uttar Pradesh. India | Canine parvovirus 2b |
| >KX425924.1 | Uttar Pradesh. India | Canine parvovirus 2a |
| >KX425923.1 | Uttar Pradesh. India | Canine parvovirus 2a |
| >KX425922.1 | Uttar Pradesh. India | Canine parvovirus 2a |
| >KX425921.1 | Uttar Pradesh. India | Canine parvovirus 2b |
| >KX425920.1 | Uttar Pradesh. India | Canine parvovirus 2c |
| >KX219742.1 | Uttar Pradesh. India | Canine parvovirus 2a |
| >KX219741.1 | Uttar Pradesh. India | Canine parvovirus 2a |
| >KX219740.1 | Uttar Pradesh. India | Canine parvovirus 2a |
| >KX219739.1 | Uttar Pradesh. India | Canine parvovirus 2a |
| >KX219738.1 | Uttar Pradesh. India | Canine parvovirus 2a |
| >KX219737.1 | Uttar Pradesh. India | Canine parvovirus 2a |
| >KX219736.1 | Uttar Pradesh. India | Canine parvovirus 2a |
| >KX219735.1 | Uttar Pradesh. India | Canine parvovirus 2a |
| >KX219734.1 | Uttar Pradesh. India | Canine parvovirus 2a |
| >KX219733.1 | Uttar Pradesh. India | Canine parvovirus 2a |
| >KX219732.1 | Uttar Pradesh. India | Canine parvovirus 2a |
| >KM386823.1 | Haibin. Heilongjiang. China | Canine parvovirus 2a |
| >KM386822.1 | Haibin. Heilongjiang. China | Canine parvovirus 2a |
| >KM386821.1 | Haibin. Heilongjiang. China | Canine parvovirus 2a |
| >KM083041.1 | Changchun. Jilin. China | Canine parvovirus |
| >KM083040.1 | Changchun. Jilin. China | Canine parvovirus |
| >KM083039.1 | Changchun. Jilin. China | Canine parvovirus |
| >KM083038.1 | Changchun. Jilin. China | Canine parvovirus |
| >KM083037.1 | Changchun. Jilin. China | Canine parvovirus |
| >KM083036.1 | Changchun. Jilin. China | Canine parvovirus |
| >JF414826.1 | Buenos Aires. Argentina | Canine parvovirus 2c |
| >JF414825.1 | Buenos Aires. Argentina | Canine parvovirus 2c |
| >JF414824.1 | Buenos Aires. Argentina | Canine parvovirus 2c |
| >JF414824.2 | Buenos Aires. Argentina | Canine parvovirus 2c |
| >JF414824.3 | Buenos Aires. Argentina | Canine parvovirus 2c |
| >JF414824.4 | Buenos Aires. Argentina | Canine parvovirus 2c |
| >JF414824.5 | Buenos Aires. Argentina | Canine parvovirus 2c |
| >JF414824.6 | Buenos Aires. Argentina | Canine parvovirus 2c |
| >JF414824.7 | Buenos Aires. Argentina | Canine parvovirus 2c |
| >JF414824.8 | Buenos Aires. Argentina | Canine parvovirus 2b |
| >JF414824.9 | Buenos Aires. Argentina | Canine parvovirus 2a |
| >JF414824.10 | Gujarat. India | Canine parvovirus |
| >JF414824.11 | Gujarat. India | Canine parvovirus |
| >JF414824.12 | Gujarat. India | Canine parvovirus |
| >JF414824.13 | Gujarat. India | Canine parvovirus |
| >JF414824.14 | Gujarat. India | Canine parvovirus |
| >JF414824.15 | Gujarat. India | Canine parvovirus |
| >JF414824.16 | Wuhan. Hubei. China | Canine parvovirus 2a |
| >EU009206.1 | Chunchon. South Korea | Canine parvovirus |
| >EU009205.1 | Chunchon. South Korea | Canine parvovirus |
| >EU009204.1 | Chunchon. South Korea | Canine parvovirus |
| >EU009203.1 | Chunchon. South Korea | Canine parvovirus |
| >EU009202.1 | Chunchon. South Korea | Canine parvovirus |
| >EU009201.1 | Chunchon. South Korea | Canine parvovirus |
| >EU009200.1 | Chunchon. South Korea | Canine parvovirus |
| >FJ432718.1 | Jilin. China | Canine parvovirus 2 |
| >FJ432717.1 | Jilin. China | Canine parvovirus 2a |
| >FJ432716.1 | Jilin. China | Canine parvovirus 2a |
| >GU452715.1 | Changchun. Jilin. China | Canine parvovirus 2a |
| >GU452714.1 | Changchun. Jilin. China | Canine parvovirus 2a |
| >GU452713.1 | Changchun. Jilin. China | Canine parvovirus 2a |
| >EU441280.1 | Jilin. China | Canine parvovirus 2a |
| >EU441279.1 | Jilin. China | Canine parvovirus 2a |
| >AY869724.1 | Taichung. Taiwan | Canine parvovirus |
| >MW715601.1 | Yanji. Jilin. China | Canine parvovirus |
| >MW715600.1 | Yanji. Jilin. China | Canine parvovirus |
| >MW715599.1 | Yanji. Jilin. China | Canine parvovirus |
| >MW715598.1 | Yanji. Jilin. China | Canine parvovirus |
| >MW715597.1 | Yanji. Jilin. China | Canine parvovirus |
| >MW715596.1 | Yanji. Jilin. China | Canine parvovirus |
| >MW715595.1 | Yanji. Jilin. China | Canine parvovirus |
| >MW715594.1 | Yanji. Jilin. China | Canine parvovirus |
| >MW715593.1 | Yanji. Jilin. China | Canine parvovirus |
| >MW691124.1 | Nanjing. Jiangsu. China | Canine parvovirus |
| >MW691123.1 | Nanjing. Jiangsu. China | Canine parvovirus |
| >MW691122.1 | Nanjing. Jiangsu. China | Canine parvovirus |
| >MW182720.1 | Nanjing. Jiangsu. China | Canine parvovirus |
| >MW182719.1 | Nanjing. Jiangsu. China | Canine parvovirus |
| >MW182718.1 | Nanjing. Jiangsu. China | Canine parvovirus |
| >MW182717.1 | Nanjing. Jiangsu. China | Canine parvovirus |
| >MW182716.1 | Nanjing. Jiangsu. China | Canine parvovirus |
| >MW182715.1 | Nanjing. Jiangsu. China | Canine parvovirus |
| >MW182714.1 | Nanjing. Jiangsu. China | Canine parvovirus |
| >MW182713.1 | Nanjing. Jiangsu. China | Canine parvovirus |
| >MW182712.1 | Nanjing. Jiangsu. China | Canine parvovirus |
| >MW182711.1 | Nanjing. Jiangsu. China | Canine parvovirus |
| >MW182710.1 | Nanjing. Jiangsu. China | Canine parvovirus |
| >MW182709.1 | Nanjing. Jiangsu. China | Canine parvovirus |
| >MW182708.1 | Nanjing. Jiangsu. China | Canine parvovirus |
| >MW182707.1 | Nanjing. Jiangsu. China | Canine parvovirus |
| >MW182706.1 | Nanjing. Jiangsu. China | Canine parvovirus |
| >MW182705.1 | Nanjing. Jiangsu. China | Canine parvovirus |
| >MW182704.1 | Nanjing. Jiangsu. China | Canine parvovirus |
| >MW182703.1 | Nanjing. Jiangsu. China | Canine parvovirus |
| >MW182702.1 | Nanjing. Jiangsu. China | Canine parvovirus |
| >MW182701.1 | Nanjing. Jiangsu. China | Canine parvovirus |
| >MW182700.1 | Nanjing. Jiangsu. China | Canine parvovirus |
| >MW182699.1 | Nanjing. Jiangsu. China | Canine parvovirus |
| >MW182698.1 | Nanjing. Jiangsu. China | Canine parvovirus |
| >MW182697.1 | Nanjing. Jiangsu. China | Canine parvovirus |
| >MW182696.1 | Nanjing. Jiangsu. China | Canine parvovirus |
| >MW182695.1 | Nanjing. Jiangsu. China | Canine parvovirus |
| >MW182694.1 | Nanjing. Jiangsu. China | Canine parvovirus |
| >MN810919.1 | Changchun. Jilin. China | Canine parvovirus |
| >MN810918.1 | Changchun. Jilin. China | Canine parvovirus |
| >MN810917.1 | Changchun. Jilin. China | Canine parvovirus |
| >MN810916.1 | Changchun. Jilin. China | Canine parvovirus |
| >MN810915.1 | Changchun. Jilin. China | Canine parvovirus |
| >MN810914.1 | Changchun. Jilin. China | Canine parvovirus |
| >MN810913.1 | Changchun. Jilin. China | Canine parvovirus |
| >MN810912.1 | Changchun. Jilin. China | Canine parvovirus |
| >MN810911.1 | Changchun. Jilin. China | Canine parvovirus |
| >MN810910.1 | Changchun. Jilin. China | Canine parvovirus |
| >MN810909.1 | Changchun. Jilin. China | Canine parvovirus |
| >MN810908.1 | Changchun. Jilin. China | Canine parvovirus |
| >MN810907.1 | Changchun. Jilin. China | Canine parvovirus |
| >MN810906.1 | Changchun. Jilin. China | Canine parvovirus |
| >MN810905.1 | Changchun. Jilin. China | Canine parvovirus |
| >MN810904.1 | Changchun. Jilin. China | Canine parvovirus |
| >MN810903.1 | Changchun. Jilin. China | Canine parvovirus |
| >MN810902.1 | Changchun. Jilin. China | Canine parvovirus |
| >MN810901.1 | Changchun. Jilin. China | Canine parvovirus |
| >MN810900.1 | Changchun. Jilin. China | Canine parvovirus |
| >MN810899.1 | Changchun. Jilin. China | Canine parvovirus |
| >MN810898.1 | Changchun. Jilin. China | Canine parvovirus |
| >MN810897.1 | Changchun. Jilin. China | Canine parvovirus |
| >MN810896.1 | Changchun. Jilin. China | Canine parvovirus |
| >MN810895.1 | Changchun. Jilin. China | Canine parvovirus |
| >MN810894.1 | Changchun. Jilin. China | Canine parvovirus |
| >MN810893.1 | Changchun. Jilin. China | Canine parvovirus |
| >MN810892.1 | Changchun. Jilin. China | Canine parvovirus |
| >MN810891.1 | Changchun. Jilin. China | Canine parvovirus |
| >MN810890.1 | Changchun. Jilin. China | Canine parvovirus |
| >MN810889.1 | Changchun. Jilin. China | Canine parvovirus |
| >MN810888.1 | Changchun. Jilin. China | Canine parvovirus |
| >MN810887.1 | Changchun. Jilin. China | Canine parvovirus |
| >MN810886.1 | Changchun. Jilin. China | Canine parvovirus |
| >MN810885.1 | Changchun. Jilin. China | Canine parvovirus |
| >MN810884.1 | Changchun. Jilin. China | Canine parvovirus |
| >MN810883.1 | Changchun. Jilin. China | Canine parvovirus |
| >MN810882.1 | Changchun. Jilin. China | Canine parvovirus |
| >MN810881.1 | Changchun. Jilin. China | Canine parvovirus |
| >MN810880.1 | Changchun. Jilin. China | Canine parvovirus |
| >MN810879.1 | Changchun. Jilin. China | Canine parvovirus |
| >MN810878.1 | Changchun. Jilin. China | Canine parvovirus |
| >MN810877.1 | Changchun. Jilin. China | Canine parvovirus |
| >MN810876.1 | Changchun. Jilin. China | Canine parvovirus |
| >MT488452.1 | Guangzhou. Guangdong. China | Canine parvovirus |
| >MH764263.1 | Gyeongsangbuk-do. Korea | Canine parvovirus 2c |
| >MH764262.1 | Gyeongsangbuk-do. Korea | Canine parvovirus 2a |
| >MH764261.1 | Gyeongsangbuk-do. Korea | Canine parvovirus 2a |
| >MK344470.1 | Rio Grande do Sul. Brasil | Canine parvovirus |
| >MK344469.1 | Rio Grande do Sul. Brasil | Canine parvovirus |
| >MK344468.1 | Rio Grande do Sul. Brasil | Canine parvovirus |
| >MK344467.1 | Rio Grande do Sul. Brasil | Canine parvovirus |
| >MK344466.1 | Rio Grande do Sul. Brasil | Canine parvovirus |
| >MK344465.1 | Rio Grande do Sul. Brasil | Canine parvovirus |
| >MK344464.1 | Rio Grande do Sul. Brasil | Canine parvovirus |
| >MK344463.1 | Rio Grande do Sul. Brasil | Canine parvovirus |
| >MK344462.1 | Rio Grande do Sul. Brasil | Canine parvovirus |
| >MK344461.1 | Rio Grande do Sul. Brasil | Canine parvovirus |
| >MK344460.1 | Rio Grande do Sul. Brasil | Canine parvovirus |
| >MK344459.1 | Rio Grande do Sul. Brasil | Canine parvovirus |
| >MK344458.1 | Rio Grande do Sul. Brasil | Canine parvovirus |
| >MK344457.1 | Rio Grande do Sul. Brasil | Canine parvovirus |
| >MK344456.1 | Rio Grande do Sul. Brasil | Canine parvovirus |
| >MK344455.1 | Rio Grande do Sul. Brasil | Canine parvovirus |
| >MK344454.1 | Rio Grande do Sul.Brasil | Canine parvovirus |
| >MK344453.1 | Rio Grande do Sul. Brasil | Canine parvovirus |
| >MK344452.1 | Rio Grande do Sul. Brasil | Canine parvovirus |
| >MK344451.1 | Rio Grande do Sul. Brasil | Canine parvovirus |
| >MK344449.1 | Rio Grande do Sul. Brasil | Canine parvovirus |
| >MK344448.1 | Rio Grande do Sul. Brasil | Canine parvovirus |
| >MK344447.1 | Rio Grande do Sul. Brasil | Canine parvovirus |
| >MK344446.1 | Rio Grande do Sul. Brasil | Canine parvovirus |
| >MK344445.1 | Rio Grande do Sul. Brasil | Canine parvovirus |
| >MK344444.1 | Rio Grande do Sul. Brasil | Canine parvovirus |
| >MK344443.1 | Rio Grande do Sul. Brasil | Canine parvovirus |
| >MK344442.1 | Rio Grande do Sul. Brasil | Canine parvovirus |
| >MK344441.1 | Rio Grande do Sul. Brasil | Canine parvovirus |
| >MK344440.1 | Rio Grande do Sul. Brasil | Canine parvovirus |
| >MK344439.1 | Rio Grande do Sul. Brasil | Canine parvovirus |
| >MK344438.1 | Rio Grande do Sul. Brasil | Canine parvovirus |
| >MK344437.1 | Rio Grande do Sul. Brasil | Canine parvovirus |
| >MK344436.1 | Rio Grande do Sul. Brasil | Canine parvovirus |
| >MK344435.1 | Rio Grande do Sul. Brasil | Canine parvovirus |
| >MK344433.1 | Rio Grande do Sul. Brasil | Canine parvovirus |
| >MF001439.1 | Yangzhou. Jiangsu. China | Canine parvovirus 2a |
| >MF001438.1 | Yangzhou. Jiangsu. China | Canine parvovirus 2a |
| >MF001437.1 | Yangzhou. Jiangsu. China | Canine parvovirus 2c |
| >MF001436.1 | Yangzhou. Jiangsu. China | Canine parvovirus 2c |
| >MF001435.1 | Yangzhou. Jiangsu. China | Canine parvovirus 2c |
| >KP893078.1 | Gyeonggi-do. Korea | Canine parvovirus |
| >KP893077.1 | Gyeonggi-do. Korea | Canine parvovirus |
| >KM924289.1 | Luoyan. Henan. China | Canine parvovirus 2a |
| >KM014812.1 | Yanji. Jilin. China | Canine parvovirus |
| >KJ186145.1 | Changchun. Jilin. China | Canine parvovirus |
| >KJ186144.1 | Changchun. Jilin. China | Canine parvovirus |
| >KJ186143.1 | Changchun. Jilin. China | Canine parvovirus |
| >KJ186142.1 | Changchun. Jilin. China | Canine parvovirus |
| >KJ186141.1 | Changchun. Jilin. China | Canine parvovirus |
| >KJ186140.1 | Changchun. Jilin. China | Canine parvovirus |
| >KJ186139.1 | Changchun. Jilin. China | Canine parvovirus |
| >KJ438805.1 | Luoyang. Henan. China | Canine parvovirus |
| >KJ438804.1 | Luoyang. Henan. China | Canine parvovirus |
| >KJ438803.1 | Luoyang. Henan. China | Canine parvovirus |
| >KJ438802.1 | Luoyang. Henan. China | Canine parvovirus |
| >KJ438801.1 | Luoyang. Henan. China | Canine parvovirus |
| >KJ438800.1 | Luoyang. Henan. China | Canine parvovirus |
| >KJ438799.1 | Luoyang. Henan. China | Canine parvovirus |
| >KJ438798.1 | Luoyang. Henan. China | Canine parvovirus |
| >KJ170681.1 | Changchun. Jilin. China | Canine parvovirus |
| >KJ170680.1 | Changchun. Jilin. China | Canine parvovirus |
| >KJ170679.1 | Changchun. Jilin. China | Canine parvovirus |
| >KJ170678.1 | Changchun. Jilin. China | Canine parvovirus |
| >KC262178.1 | Changchun. Jilin. China | Canine parvovirus |
| >KC473946.1 | Guangzhou. Guangdong. China | Feline parvovirus |
| >JQ743906.1 | Haibin. Heilongjiang. China | Canine parvovirus 2a |
| >JQ743905.1 | Haibin. Heilongjiang. China | Canine parvovirus 2a |
| >JQ743904.1 | Haibin. Heilongjiang. China | Canine parvovirus 2a |
| >JQ743903.1 | Haibin. Heilongjiang. China | Canine parvovirus 2a |
| >JQ743902.1 | Haibin. Heilongjiang. China | Canine parvovirus 2a |
| >JQ743901.1 | Haibin. Heilongjiang. China | Canine parvovirus 2a |
| >JQ743900.1 | Haibin. Heilongjiang. China | Canine parvovirus 2a |
| >JQ743899.1 | Haibin. Heilongjiang. China | Canine parvovirus 2a |
| >JQ743898.1 | Haibin. Heilongjiang. China | Canine parvovirus 2a |
| >JQ743897.1 | Haibin. Heilongjiang. China | Canine parvovirus |
| >JQ743896.1 | Haibin. Heilongjiang. China | Canine parvovirus 2a |
| >JQ743895.1 | Haibin. Heilongjiang. China | Canine parvovirus 2a |
| >JQ743894.1 | Haibin. Heilongjiang. China | Canine parvovirus 2b |
| >JQ743893.1 | Haibin. Heilongjiang. China | Canine parvovirus 2b |
| >JQ743892.1 | Haibin. Heilongjiang. China | Canine parvovirus 2b |
| >JQ743891.1 | Haibin. Heilongjiang. China | Canine parvovirus 2b |
| >JQ743890.1 | Haibin. Heilongjiang. China | Canine parvovirus 2b |
| >OM964633.1 | Changchun. Jilin. China | Canine parvovirus |
| >OM964632.1 | Changchun. Jilin. China | Canine parvovirus |
| >OM964631.1 | Changchun. Jilin. China | Canine parvovirus |
| >OM918785.1 | Yanji. Jilin. China | Feline parvovirus |
| >OM918784.1 | Yanji. Jilin. China | Feline parvovirus |
| >OM918783.1 | Yanji. Jilin. China | Feline parvovirus |
| >OM918782.1 | Yanji. Jilin. China | Feline parvovirus |
| >OM918781.1 | Yanji. Jilin. China | Feline parvovirus |
| >OM918780.1 | Yanji. Jilin. China | Feline parvovirus |
| >OM918779.1 | Yanji. Jilin. China | Feline parvovirus |
| >OM918778.1 | Yanji. Jilin. China | Feline parvovirus |
| >OM918777.1 | Yanji. Jilin. China | Feline parvovirus |
| >OM918776.1 | Yanji. Jilin. China | Feline parvovirus |
| >OM918775.1 | Yanji. Jilin. China | Feline parvovirus |
| >OM918774.1 | Yanji. Jilin. China | Feline parvovirus |
| >OM918773.1 | Yanji. Jilin. China | Feline parvovirus |
| >OM918772.1 | Yanji. Jilin. China | Feline parvovirus |
| >OM918771.1 | Yanji. Jilin. China | Feline parvovirus |
| >OM918770.1 | Yanji. Jilin. China | Feline parvovirus |
| >OM885384.1 | Yanji. Jilin. China | Feline parvovirus |
| >OM885383.1 | Yanji. Jilin. China | Feline parvovirus |
| >OM885382.1 | Yanji. Jilin. China | Feline parvovirus |
| >OM885381.1 | Yanji. Jilin. China | Feline parvovirus |
| >OM885380.1 | Yanji. Jilin. China | Feline parvovirus |
| >OM885379.1 | Yanji. Jilin. China | Feline parvovirus |
| >OM885378.1 | Yanji. Jilin. China | Feline parvovirus |
| >OM885377.1 | Yanji. Jilin. China | Feline parvovirus |
| >OM885376.1 | Yanji. Jilin. China | Feline parvovirus |
| >OM885375.1 | Yanji. Jilin. China | Feline parvovirus |
| >OM885374.1 | Yanji. Jilin. China | Feline parvovirus |
| >OM885373.1 | Yanji. Jilin. China | Feline parvovirus |
| >OM322821.1 | Yanji. Jilin. China | Feline parvovirus |
| >ON605652.1 | Australia | Feline parvovirus |
| >MZ442314.1 | Chengdu. Sichuan. China | Feline parvovirus |
| >MZ442313.1 | Chengdu. Sichuan. China | Feline parvovirus |
| >MZ442312.1 | Chengdu. Sichuan. China | Feline parvovirus |
| >MZ442311.1 | Chengdu. Sichuan. China | Feline parvovirus |
| >MZ442310.1 | Chengdu. Sichuan. China | Feline parvovirus |
| >MZ442309.1 | Chengdu. Sichuan. China | Feline parvovirus |
| >MZ442308.1 | Chengdu. Sichuan. China | Feline parvovirus |
| >MZ442307.1 | Chengdu. Sichuan. China | Feline parvovirus |
| >MZ442306.1 | Chengdu. Sichuan. China | Feline parvovirus |
| >MZ442305.1 | Chengdu. Sichuan. China | Feline parvovirus |
| >MZ442304.1 | Chengdu. Sichuan. China | Feline parvovirus |
| >MZ442303.1 | Chengdu. Sichuan. China | Feline parvovirus |
| >MZ442302.1 | Chengdu. Sichuan. China | Feline parvovirus |
| >MW495851.1 | Jingzhou. Hubei. China | Canine parvovirus |
| >OL330980.1 | Mazandaran. Iran | Canine parvovirus 2 |
| >OL330979.1 | Mazandaran. Iran | Canine parvovirus 2 |
| >OL330978.1 | Mazandaran. Iran | Canine parvovirus 2 |
| >OL330977.1 | Mazandaran. Iran | Canine parvovirus 2 |
| >OL330976.1 | Mazandaran. Iran | Canine parvovirus |
| >MT525961.1 | Yanji. Jilin. China | Canine parvovirus |
| >MT525960.1 | Yanji. Jilin. China | Canine parvovirus |
| >MT525959.1 | Yanji. Jilin. China | Canine parvovirus |
| >MT525958.1 | Yanji. Jilin. China | Canine parvovirus |
| >MT525957.1 | Yanji. Jilin. China | Canine parvovirus |
| >MT525956.1 | Yanji. Jilin. China | Canine parvovirus |
| >MT525954.1 | Yanji. Jilin. China | Canine parvovirus |
| >MT525953.1 | Yanji. Jilin. China | Canine parvovirus |
| >MT525952.1 | Yanji. Jilin. China | Canine parvovirus |
| >MT488468.1 | Guangzhou. Guangdong. China | Canine parvovirus |
| >MT488467.1 | Guangzhou. Guangdong. China | Canine parvovirus |
| >MT488466.1 | Guangzhou. Guangdong. China | Canine parvovirus |
| >MT488465.1 | Guangzhou. Guangdong. China | Canine parvovirus |
| >MT488464.1 | Guangzhou. Guangdong. China | Canine parvovirus |
| >MT488463.1 | Guangzhou. Guangdong. China | Canine parvovirus |
| >MT488462.1 | Guangzhou. Guangdong. China | Canine parvovirus |
| >MT488461.1 | Guangzhou. Guangdong. China | Canine parvovirus |
| >MT488460.1 | Guangzhou. Guangdong. China | Canine parvovirus |
| >MT488459.1 | Guangzhou. Guangdong. China | Canine parvovirus |
| >MT488458.1 | Guangzhou. Guangdong. China | Canine parvovirus |
| >MT488457.1 | Guangzhou. Guangdong. China | Canine parvovirus |
| >MT488456.1 | Guangzhou. Guangdong. China | Canine parvovirus |
| >MT488455.1 | Guangzhou. Guangdong. China | Canine parvovirus |
| >MT488454.1 | Guangzhou. Guangdong. China | Canine parvovirus |
| >MT488453.1 | Guangzhou. Guangdong. China | Canine parvovirus |
| >MK357737.1 | Pingtung. Taiwan | Canine parvovirus |
| >MK357736.1 | Pingtung. Taiwan | Canine parvovirus |
| >MK357735.1 | Pingtung. Taiwan | Canine parvovirus |
| >MK357734.1 | Pingtung. Taiwan | Canine parvovirus |
| >MK357733.1 | Pingtung. Taiwan | Canine parvovirus |
| >MK357732.1 | Pingtung. Taiwan | Canine parvovirus |
| >MK357731.1 | Pingtung. Taiwan | Canine parvovirus |
| >MK357730.1 | Pingtung. Taiwan | Canine parvovirus |
| >MK357729.1 | Pingtung. Taiwan | Canine parvovirus |
| >MK357728.1 | Pingtung. Taiwan | Canine parvovirus |
| >MK357727.1 | Pingtung. Taiwan | Canine parvovirus |
| >MK357726.1 | Pingtung. Taiwan | Canine parvovirus |
| >MK357725.1 | Pingtung. Taiwan | Canine parvovirus |
| >MK357724.1 | Pingtung. Taiwan | Canine parvovirus |
| >MK357723.1 | Pingtung. Taiwan | Canine parvovirus |
| >MK357722.1 | Pingtung. Taiwan | Canine parvovirus |
| >MK357721.1 | Pingtung. Taiwan | Canine parvovirus |
| >MN055687.1 | Changchun. Jilin. China | Canine parvovirus |
| >MK268683.1 | Qingdao. Shandong. China | Canine parvovirus 2b |
| >MK268682.1 | Qingdao. Shandong. China | Canine parvovirus 2c |
| >MK268681.1 | Qingdao. Shandong. China | Canine parvovirus 2a |
| >KC881278.1 | Wuhan. Hubei. China | Canine parvovirus |
| >JN403045.1 | Xi'an. Shanxi. China | Canine parvovirus 2a |
| >EF599098.2 | South Korea | Canine parvovirus |
| >EF599097.1 | South Korea | Canine parvovirus |
| >EF599096.1 | Korea | Canine parvovirus |
| >KP685412.1 | Changchun. Jilin. China | Protoparvovirus carnivoran1 |
| >KP685411.1 | Changchun. Jilin. China | Protoparvovirus carnivoran1 |
| >KP685410.1 | Changchun. Jilin. China | Protoparvovirus carnivoran1 |
| >FJ231389.1 | Changchun. Jilin. China | Feline panleukopenia virus monkey/BJ-22/2008/CHN |
| >MW239610.1 | Ha Noi. VietNam | Protoparvovirus carnivoran1 |
| >MW239609.1 | Ha Noi. VietNam | Protoparvovirus carnivoran1 |
| >MW239608.1 | Ha Noi. VietNam | Protoparvovirus carnivoran1 |
| >MW239607.1 | Ha Noi. VietNam | Protoparvovirus carnivoran1 |
| >MW239606.1 | Ha Noi. VietNam | Protoparvovirus carnivoran1 |
| >MW239605.1 | Ha Noi. VietNam | Protoparvovirus carnivoran1 |
| >MW239604.1 | Ha Noi. VietNam | Protoparvovirus carnivoran1 |
| >MW239603.1 | Ha Noi. VietNam | Protoparvovirus carnivoran1 |
| >MW239602.1 | Ha Noi. VietNam | Protoparvovirus carnivoran1 |
| >MW239601.1 | Ha Noi. VietNam | Protoparvovirus carnivoran1 |
| >MW239600.1 | Ha Noi. VietNam | Protoparvovirus carnivoran1 |
| >MW239599.1 | Ha Noi. VietNam | Protoparvovirus carnivoran1 |
| >MW239598.1 | Ha Noi. VietNam | Protoparvovirus carnivoran1 |
| >MW239597.1 | Ha Noi. VietNam | Protoparvovirus carnivoran1 |
| >MW239596.1 | Ha Noi. VietNam | Protoparvovirus carnivoran1 |
| >MW239595.1 | Ha Noi. VietNam | Protoparvovirus carnivoran1 |
| >MW239594.1 | Ha Noi. VietNam | Protoparvovirus carnivoran1 |
| >MW239593.1 | Ha Noi. VietNam | Protoparvovirus carnivoran1 |
| >MW239592.1 | Ha Noi. VietNam | Protoparvovirus carnivoran1 |
| >MW239591.1 | Ha Noi. VietNam | Protoparvovirus carnivoran1 |
| >MW239590.1 | Ha Noi. VietNam | Protoparvovirus carnivoran1 |
| >MW239589.1 | Ha Noi. VietNam | Protoparvovirus carnivoran1 |
| >MW239588.1 | Ha Noi. VietNam | Protoparvovirus carnivoran1 |
| >MW239587.1 | Ha Noi. VietNam | Protoparvovirus carnivoran1 |
| >MW239586.1 | Ha Noi. VietNam | Protoparvovirus carnivoran1 |
| >MW239585.1 | Ha Noi. VietNam | Protoparvovirus carnivoran1 |
| >MW239584.1 | Ha Noi. VietNam | Protoparvovirus carnivoran1 |
| >MW239583.1 | Ha Noi. VietNam | Protoparvovirus carnivoran1 |
| >MW239582.1 | Ha Noi. VietNam | Protoparvovirus carnivoran1 |
| >MW239581.1 | Ha Noi. VietNam | Protoparvovirus carnivoran1 |
| >MW239580.1 | Ha Noi. VietNam | Protoparvovirus carnivoran1 |
| >MW239579.1 | Ha Noi. VietNam | Protoparvovirus carnivoran1 |
| >MW239578.1 | Ha Noi. VietNam | Protoparvovirus carnivoran1 |
| >MW239577.1 | Ha Noi. VietNam | Protoparvovirus carnivoran1 |
| >MT250783.1 | Changchun. Jilin. China | Mink enteritis virus |
| >MK357743.1 | Pingtung. Taiwan | Feline panleukopenia virus |
| >MK357742.1 | Pingtung. Taiwan | Feline panleukopenia virus |
| >MK357741.1 | Pingtung. Taiwan | Feline panleukopenia virus |
| >MK357740.1 | Pingtung. Taiwan | Feline panleukopenia virus |
| >MK357739.1 | Pingtung. Taiwan | Feline panleukopenia virus |
| >MK357738.1 | Pingtung. Taiwan | Feline panleukopenia virus |
| >MK256738.1 | Kunming. Yunnan. China | Canine parvovirus |
| >KJ186148.1 | Changchun. Jilin. China | Mink enteritis virus |
| >KJ186147.1 | Changchun. Jilin. China | Mink enteritis virus |
| >KJ186146.1 | Changchun. Jilin. China | Mink enteritis virus |
| >MT892650.1 | Xianyang. Shanxi. China | Feline panleukopenia virus |
| >MK301396.1 | Qingdao. Shandong. China | Feline panleukopenia virus |
| >HQ184204.1 | Gyeonggi. Korea | Feline panleukopenia virus |
| >HQ184203.1 | Gyeonggi. Korea | Feline panleukopenia virus |
| >HQ184202.1 | Gyeonggi. Korea | Feline panleukopenia virus |
| >HQ184201.1 | Gyeonggi. Korea | Feline panleukopenia virus |
| >HQ184200.1 | Gyeonggi. Korea | Feline panleukopenia virus |
| >HQ184199.1 | Gyeonggi. Korea | Feline panleukopenia virus |
| >HQ184198.1 | Gyeonggi. Korea | Feline panleukopenia virus |
| >HQ184197.1 | Gyeonggi. Korea | Feline panleukopenia virus |
| >HQ184196.1 | Gyeonggi. Korea | Feline panleukopenia virus |
| >HQ184195.1 | Gyeonggi. Korea | Feline panleukopenia virus |
| >HQ184194.1 | Gyeonggi. Korea | Feline panleukopenia virus |
| >HQ184193.1 | Gyeonggi. Korea | Feline panleukopenia virus |
| >HQ184192.1 | Gyeonggi. Korea | Feline panleukopenia virus |
| >HQ184191.1 | Gyeonggi. Korea | Feline panleukopenia virus |
| >HQ184190.1 | Gyeonggi. Korea | Feline panleukopenia virus |
| >HQ184189.1 | Gyeonggi. Korea | Feline panleukopenia virus |
| >MZ209402.1 | Uttar Pradesh. India | Canine parvovirus |
| >MZ209401.1 | Uttar Pradesh. India | Canine parvovirus |
| >MH581185.1 | Changchun. Jilin. China | Canine parvovirus |
| >MH581184.1 | Changchun. Jilin. China | Canine parvovirus |
| >MH581183.1 | Changchun. Jilin. China | Canine parvovirus |
| >MH581182.1 | Changchun. Jilin. China | Canine parvovirus |
| >MH581181.1 | Changchun. Jilin. China | Canine parvovirus |
| >MH581180.1 | Changchun. Jilin. China | Canine parvovirus |
| >MH581179.1 | Changchun. Jilin. China | Canine parvovirus |
| >MH581178.1 | Changchun. Jilin. China | Canine parvovirus |
| >MH581177.1 | Changchun. Jilin. China | Canine parvovirus |
| >MH581176.1 | Changchun. Jilin. China | Canine parvovirus |
| >MH581175.1 | Changchun. Jilin. China | Canine parvovirus |
| >MH581174.1 | Changchun. Jilin .China | Canine parvovirus |
| >DQ903936.1 | Ya'an. Sichuan. China | Canine parvovirus |
| >GQ169553.1 | Jilin. China | Canine parvovirus |
| >GQ169552.1 | Jilin. China | Canine parvovirus |
| >GQ169551.1 | Jilin. China | Canine parvovirus |
| >GQ169550.1 | Jilin. China | Canine parvovirus |
| >GQ169549.1 | Jilin. China | Canine parvovirus |
| >GQ169548.1 | Jilin. China | Canine parvovirus |
| >GQ169547.1 | Jilin. China | Canine parvovirus |
| >GQ169546.1 | Jilin. China | Canine parvovirus |
| >GQ169545.1 | Jilin. China | Canine parvovirus |
| >GQ169544.1 | Jilin. China | Canine parvovirus |
| >GQ169543.1 | Jilin. China | Canine parvovirus |
| >GQ169542.1 | Jilin. China | Canine parvovirus |
| >GQ169541.1 | Jilin. China | Canine parvovirus |
| >GQ169540.1 | Jilin. China | Canine parvovirus |
| >GQ169539.1 | Jilin. China | Canine parvovirus |
| >GQ169538.1 | Jilin. China | Canine parvovirus |
| >GQ169537.1 | Jilin. China | Canine parvovirus |
| >EF592511.1 | Taichung. Taiwan | Canine parvovirus |
| >EU213085.1 | Hangzhou. Zhejiang. China | Canine parvovirus |
| >EU213084.1 | Hangzhou. Zhejiang. China | Canine parvovirus |
| >EU213083.1 | Hangzhou. Zhejiang. China | Canine parvovirus |
| >EU213082.1 | Hangzhou. Zhejiang. China | Canine parvovirus |
| >EU213081.1 | Hangzhou. Zhejiang. China | Canine parvovirus |
| >EU213080.1 | Hangzhou. Zhejiang. China | Canine parvovirus |
| >EU213079.1 | Hangzhou. Zhejiang. China | Canine parvovirus |
| >EU213078.1 | Hangzhou. Zhejiang. China | Canine parvovirus |
| >EU213077.1 | Hangzhou. Zhejiang. China | Canine parvovirus |
| >EU213076.1 | Hangzhou. Zhejiang. China | Canine parvovirus |
| >EU213075.1 | Hangzhou. Zhejiang. China | Canine parvovirus |
| >EU213074.1 | Hangzhou. Zhejiang. China | Canine parvovirus |
| >EU213073.1 | Hangzhou. Zhejiang. China | Canine parvovirus |
| >ON479062.1 | Palermo. Italy | Canine parvovirus 2 |
| >ON479061.1 | Palermo. Italy | Canine parvovirus 2 |
| >ON479060.1 | Palermo. Italy | Canine parvovirus 2 |
| >ON479059.1 | Palermo. Italy | Canine parvovirus 2 |
| >ON479058.1 | Palermo. Italy | Canine parvovirus 2 |
| >ON479057.1 | Palermo. Italy | Canine parvovirus 2 |
| >MK295775.1 | Changchun. Jilin. China | Feline parvovirus |
| >MF996334.1 | Tai'an. Shandong. China | Canine parvovirus 2 |
| >MF996333.1 | Tai'an. Shandong. China | Canine parvovirus 2 |
| >MF996332.1 | Tai'an. Shandong. China | Canine parvovirus 2 |
| >KT275256.1 | Porto. Portugal | Canine parvovirus 2c |
| >KT275255.1 | Porto. Portugal | Canine parvovirus 2c |
| >KT275254.1 | Porto. Portugal | Canine parvovirus 2c |
| >KT275253.1 | Porto. Portugal | Canine parvovirus 2c |
| >KT275252.1 | Porto. Portugal | Canine parvovirus 2c |
| >JX305965.1 | Bari. Italy | Canine parvovirus 2 |
| >JX305964.1 | Bari. Italy | Canine parvovirus 2 |
| >JX305963.1 | Bari. Italy | Canine parvovirus 2 |
| >JX305962.1 | Bari. Italy | Canine parvovirus 2 |
| >JX305961.1 | Bari. Italy | Canine parvovirus 2 |
| >JX305960.1 | Bari. Italy | Canine parvovirus 2 |
| >JX305959.1 | Bari. Italy | Canine parvovirus 2 |
| >JX305958.1 | Bari. Italy | Canine parvovirus 2 |
| >JX305957.1 | Bari. Italy | Canine parvovirus 2 |
| >JX305956.1 | Bari. Italy | Canine parvovirus 2 |
| >JX305955.1 | Bari. Italy | Canine parvovirus 2 |
| >JX305954.1 | Bari. Italy | Canine parvovirus 2 |
| >JX305953.1 | Bari. Italy | Canine parvovirus 2 |
| >JX305952.1 | Bari. Italy | Canine parvovirus 2 |
| >JX305951.1 | Bari. Italy | Canine parvovirus 2 |
| >JX305950.1 | Bari. Italy | Canine parvovirus 2 |
| >JX305949.1 | Bari. Italy | Canine parvovirus 2 |
| >JX305948.1 | Bari. Italy | Canine parvovirus 2 |
| >JX305947.1 | Bari. Italy | Canine parvovirus 2 |
| >JX305946.1 | Bari. Italy | Canine parvovirus 2 |
| >JX048608.1 | Chiayi City. Taiwan | Feline parvovirus |
| >JN867607.1 | NY.USA | Canine parvovirus |
| >JN867606.1 | NY.USA | Canine parvovirus |
| >JN867605.1 | NY.USA | Canine parvovirus |
| >JN867604.1 | NY.USA | Canine parvovirus |
| >JN867603.1 | NY.USA | Canine parvovirus |
| >JN867602.1 | NY.USA | Canine parvovirus |
| >JN867601.1 | NY.USA | Canine parvovirus |
| >JN867600.1 | NY.USA | Canine parvovirus |
| >JN867599.1 | NY.USA | Canine parvovirus |
| >JN867598.1 | NY.USA | Canine parvovirus |
| >JN867597.1 | NY.USA | Canine parvovirus |
| >DQ354068.1 | Beijing. China | Canine parvovirus |
| >OK649761.1 | Shanghai. China | Canine parvovirus |
| >OK649760.1 | Shanghai. China | Canine parvovirus |
| >OK649759.1 | Shanghai. China | Canine parvovirus |
| >OK649758.1 | Shanghai. China | Canine parvovirus |
| >OK649757.1 | Shanghai. China | Canine parvovirus |
| >OK649756.1 | Shanghai. China | Canine parvovirus |
| >LC622067.1 | Aomori. Japan | Canine parvovirus 2 |
| >LC622066.1 | Aomori. Japan | Canine parvovirus 2 |
| >LC622065.1 | Aomori. Japan | Canine parvovirus 2 |
| >LC622064.1 | Aomori. Japan | Canine parvovirus 2 |
| >LC622063.1 | Aomori. Japan | Canine parvovirus 2 |
| >LC622062.1 | Aomori. Japan | Canine parvovirus 2 |
| >LC622061.1 | Aomori. Japan | Canine parvovirus 2 |
| >LC622060.1 | Aomori. Japan | Canine parvovirus 2 |
| >LC622059.1 | Aomori. Japan | Canine parvovirus 2 |
| >LC622058.1 | Aomori. Japan | Canine parvovirus 2 |
| >LC622057.1 | Aomori. Japan | Canine parvovirus 2 |
| >LC622056.1 | Aomori. Japan | Canine parvovirus 2 |
| >LC622055.1 | Aomori. Japan | Canine parvovirus 2 |
| >LC622054.1 | Aomori. Japan | Canine parvovirus 2 |
| >LC622053.1 | Aomori. Japan | Canine parvovirus 2 |
| >LC622052.1 | Aomori. Japan | Canine parvovirus 2 |
| >LC622051.1 | Aomori. Japan | Canine parvovirus 2 |
| >LC622050.1 | Aomori. Japan | Canine parvovirus 2 |
| >LC622049.1 | Aomori. Japan | Canine parvovirus 2 |
| >LC622048.1 | Aomori. Japan | Canine parvovirus 2 |
| >LC622047.1 | Aomori. Japan | Canine parvovirus 2 |
| >LC622046.1 | Aomori. Japan | Canine parvovirus 2 |
| >LC622045.1 | Aomori. Japan | Canine parvovirus 2 |
| >LC622044.1 | Aomori. Japan | Canine parvovirus 2 |
| >LC622043.1 | Aomori. Japan | Canine parvovirus 2 |
| >LC622042.1 | Aomori. Japan | Canine parvovirus 2 |
| >LC622041.1 | Aomori. Japan | Canine parvovirus 2 |
| >LC622040.1 | Aomori. Japan | Canine parvovirus 2 |
| >LC622039.1 | Aomori. Japan | Canine parvovirus 2 |
| >LC622038.1 | Aomori. Japan | Canine parvovirus 2 |
| >LC622037.1 | Aomori. Japan | Canine parvovirus 2 |
| >LC622036.1 | Aomori. Japan | Canine parvovirus 2 |
| >LC622035.1 | Aomori. Japan | Canine parvovirus 2 |
| >LC622034.1 | Aomori. Japan | Canine parvovirus 2 |
| >LC622033.1 | Aomori. Japan | Canine parvovirus 2 |
| >LC622032.1 | Aomori. Japan | Canine parvovirus 2 |
| >LC622031.1 | Aomori. Japan | Canine parvovirus 2 |
| >LC622030.1 | Aomori. Japan | Canine parvovirus 2 |
| >LC622029.1 | Aomori. Japan | Canine parvovirus 2 |
| >LC622028.1 | Aomori. Japan | Canine parvovirus 2 |
| >LC622027.1 | Aomori. Japan | Canine parvovirus 2 |
| >LC622026.1 | Aomori. Japan | Canine parvovirus 2 |
| >LC622025.1 | Aomori. Japan | Canine parvovirus 2 |
| >LC622024.1 | Aomori. Japan | Canine parvovirus 2 |
| >LC622023.1 | Aomori. Japan | Canine parvovirus 2 |
| >LC622022.1 | Aomori. Japan | Canine parvovirus 2 |
| >LC622021.1 | Aomori. Japan | Canine parvovirus 2 |
| >LC622020.1 | Aomori. Japan | Canine parvovirus 2 |
| >LC622019.1 | Aomori. Japan | Canine parvovirus 2 |
| >LC622018.1 | Aomori. Japan | Canine parvovirus 2 |
| >LC622017.1 | Aomori. Japan | Canine parvovirus 2 |
| >LC622016.1 | Aomori. Japan | Canine parvovirus 2 |
| >LC622015.1 | Aomori. Japan | Canine parvovirus 2 |
| >LC622014.1 | Aomori. Japan | Canine parvovirus 2 |
| >LC622013.1 | Aomori. Japan | Canine parvovirus 2 |
| >LC622012.1 | Aomori. Japan | Canine parvovirus 2 |
| >LC622011.1 | Aomori. Japan | Canine parvovirus 2 |
| >LC622010.1 | Aomori. Japan | Canine parvovirus 2 |
| >LC622009.1 | Aomori. Japan | Canine parvovirus 2 |
| >LC622008.1 | Aomori. Japan | Canine parvovirus 2 |
| >LC622007.1 | Aomori. Japan | Canine parvovirus 2 |
| >LC622006.1 | Aomori. Japan | Canine parvovirus 2 |
| >LC622005.1 | Aomori. Japan | Canine parvovirus 2 |
| >LC622004.1 | Aomori. Japan | Canine parvovirus 2 |
| >LC622003.1 | Aomori. Japan | Canine parvovirus 2 |
| >LC622002.1 | Aomori. Japan | Canine parvovirus 2 |
| >LC622001.1 | Aomori. Japan | Canine parvovirus 2 |
| >LC622000.1 | Aomori. Japan | Canine parvovirus 2 |
| >LC621999.1 | Aomori. Japan | Canine parvovirus 2 |
| >LC621998.1 | Aomori. Japan | Canine parvovirus 2 |
| >LC621997.1 | Aomori. Japan | Canine parvovirus 2 |
| >LC621996.1 | Aomori. Japan | Canine parvovirus 2 |
| >LC621995.1 | Aomori. Japan | Canine parvovirus 2 |
| >LC621994.1 | Aomori. Japan | Canine parvovirus 2 |
| >LC621993.1 | Aomori. Japan | Canine parvovirus 2 |
| >LC621992.1 | Aomori. Japan | Canine parvovirus 2 |
| >LC621991.1 | Aomori. Japan | Canine parvovirus 2 |
| >LC621990.1 | Aomori. Japan | Canine parvovirus 2 |
| >LC621989.1 | Aomori. Japan | Canine parvovirus 2 |
| >LC621988.1 | Aomori. Japan | Canine parvovirus 2 |
| >LC621987.1 | Aomori. Japan | Canine parvovirus 2 |
| >LC621986.1 | Aomori. Japan | Canine parvovirus 2 |
| >LC621985.1 | Aomori. Japan | Canine parvovirus 2 |
| >LC621984.1 | Aomori. Japan | Canine parvovirus 2 |
| >LC621983.1 | Aomori. Japan | Canine parvovirus 2 |
| >LC621982.1 | Aomori. Japan | Canine parvovirus 2 |
| >LC621981.1 | Aomori. Japan | Canine parvovirus 2 |
| >LC621980.1 | Aomori. Japan | Canine parvovirus 2 |
| >LC621979.1 | Aomori. Japan | Canine parvovirus 2 |
| >LC621978.1 | Aomori. Japan | Canine parvovirus 2 |
| >LC621977.1 | Aomori. Japan | Canine parvovirus 2 |
| >LC621976.1 | Aomori. Japan | Canine parvovirus 2 |
| >LC621975.1 | Aomori. Japan | Canine parvovirus 2 |
| >LC621974.1 | Aomori. Japan | Canine parvovirus 2 |
| >LC621973.1 | Aomori. Japan | Canine parvovirus 2 |
| >LC621972.1 | Aomori. Japan | Canine parvovirus 2 |
| >LC621971.1 | Aomori. Japan | Canine parvovirus 2 |
| >LC621970.1 | Aomori. Japan | Canine parvovirus 2 |
| >LC621969.1 | Aomori. Japan | Canine parvovirus 2 |
| >LC621968.1 | Aomori. Japan | Canine parvovirus 2 |
| >LC621967.1 | Aomori. Japan | Canine parvovirus 2 |
| >LC621966.1 | Aomori. Japan | Canine parvovirus 2 |
| >LC621965.1 | Aomori. Japan | Canine parvovirus 2 |
| >LC621964.1 | Aomori. Japan | Canine parvovirus 2 |
| >LC621963.1 | Aomori. Japan | Canine parvovirus 2 |
| >LC621962.1 | Aomori. Japan | Canine parvovirus 2 |
| >LC621961.1 | Aomori. Japan | Canine parvovirus 2 |
| >LC621960.1 | Aomori. Japan | Canine parvovirus 2 |
| >LC621959.1 | Aomori. Japan | Canine parvovirus 2 |
| >LC621958.1 | Aomori. Japan | Canine parvovirus 2 |
| >LC621957.1 | Aomori. Japan | Canine parvovirus 2 |
| >LC621956.1 | Aomori. Japan | Canine parvovirus 2 |
| >LC621955.1 | Aomori. Japan | Canine parvovirus 2 |
| >LC621954.1 | Aomori. Japan | Canine parvovirus 2 |
| >LC621953.1 | Aomori. Japan | Canine parvovirus 2 |
| >LC621952.1 | Aomori. Japan | Canine parvovirus 2 |
| >LC621951.1 | Aomori. Japan | Canine parvovirus 2 |
| >LC621950.1 | Aomori. Japan | Canine parvovirus 2 |
| >LC621949.1 | Aomori. Japan | Canine parvovirus 2 |
| >LC621948.1 | Aomori. Japan | Canine parvovirus 2 |
| >LC621947.1 | Aomori. Japan | Canine parvovirus 2 |
| >LC621946.1 | Aomori. Japan | Canine parvovirus 2 |
| >LC621945.1 | Aomori. Japan | Canine parvovirus 2 |
| >LC621944.1 | Aomori. Japan | Canine parvovirus 2 |
| >LC621943.1 | Aomori. Japan | Canine parvovirus 2 |
| >LC621942.1 | Aomori. Japan | Canine parvovirus 2 |
| >LC621941.1 | Aomori. Japan | Canine parvovirus 2 |
| >LC621940.1 | Aomori. Japan | Canine parvovirus 2 |
| >LC621939.1 | Aomori. Japan | Canine parvovirus 2 |
| >LC621938.1 | Aomori. Japan | Canine parvovirus 2 |
| >LC621937.1 | Aomori. Japan | Canine parvovirus 2 |
| >LC621936.1 | Aomori. Japan | Canine parvovirus 2 |
| >LC621935.1 | Aomori. Japan | Canine parvovirus 2 |
| >LC621934.1 | Aomori. Japan | Canine parvovirus 2 |
| >LC621933.1 | Aomori. Japan | Canine parvovirus 2 |
| >LC621932.1 | Aomori. Japan | Canine parvovirus 2 |
| >LC621931.1 | Aomori. Japan | Canine parvovirus 2 |
| >LC621930.1 | Aomori. Japan | Canine parvovirus 2 |
| >LC621929.1 | Aomori. Japan | Canine parvovirus 2 |
| >LC621928.1 | Aomori. Japan | Canine parvovirus 2 |
| >LC621927.1 | Aomori. Japan | Canine parvovirus 2 |
| >LC621926.1 | Aomori. Japan | Canine parvovirus 2 |
| >LC621925.1 | Aomori. Japan | Canine parvovirus 2 |
| >LC621924.1 | Aomori. Japan | Canine parvovirus 2 |
| >LC621923.1 | Aomori. Japan | Canine parvovirus 2 |
| >LC621922.1 | Aomori. Japan | Canine parvovirus 2 |
| >LC621921.1 | Aomori. Japan | Canine parvovirus 2 |
| >LC621920.1 | Aomori. Japan | Canine parvovirus 2 |
| >LC621919.1 | Aomori. Japan | Canine parvovirus 2 |
| >LC621918.1 | Aomori. Japan | Canine parvovirus 2 |
| >LC621917.1 | Aomori. Japan | Canine parvovirus 2 |
| >LC621916.1 | Aomori. Japan | Canine parvovirus 2 |
| >LC621915.1 | Aomori. Japan | Canine parvovirus 2 |
| >LC621914.1 | Aomori. Japan | Canine parvovirus 2 |
| >LC621913.1 | Aomori. Japan | Canine parvovirus 2 |
| >LC621912.1 | Aomori. Japan | Canine parvovirus 2 |
| >LC621911.1 | Aomori. Japan | Canine parvovirus 2 |
| >LC621910.1 | Aomori. Japan | Canine parvovirus 2 |
| >MT078782.1 | Pondicherry. India | Canine parvovirus 2a |
| >MT078781.1 | Pondicherry. India | Canine parvovirus 2a |
| >MT078780.1 | Pondicherry. India | Canine parvovirus 2a |
| >MT078779.1 | Pondicherry. India | Canine parvovirus 2a |
| >MT078778.1 | Pondicherry. India | Canine parvovirus 2a |
| >MT078777.1 | Pondicherry. India | Canine parvovirus 2a |
| >MT078776.1 | Pondicherry. India | Canine parvovirus 2a |
| >MT078775.1 | Pondicherry. India | Canine parvovirus 2a |
| >MT078774.1 | Pondicherry. India | Canine parvovirus 2a |
| >MT078773.1 | Pondicherry. India | Canine parvovirus 2a |
| >MT078772.1 | Pondicherry. India | Canine parvovirus 2a |
| >MT078794.1 | Pondicherry. India | Canine parvovirus 2a |
| >MT078793.1 | Pondicherry. India | Canine parvovirus 2a |
| >MT078792.1 | Pondicherry. India | Canine parvovirus 2a |
| >MT078791.1 | Pondicherry. India | Canine parvovirus 2a |
| >MT078790.1 | Pondicherry. India | Canine parvovirus 2a |
| >MT078789.1 | Pondicherry. India | Canine parvovirus 2a |
| >MT078788.1 | Pondicherry. India | Canine parvovirus 2a |
| >MT078787.1 | Pondicherry. India | Canine parvovirus 2a |
| >MT078786.1 | Pondicherry. India | Canine parvovirus 2a |
| >MT078785.1 | Pondicherry. India | Canine parvovirus 2a |
| >MT078784.1 | Pondicherry. India | Canine parvovirus 2a |
| >MT078783.1 | Pondicherry. India | Canine parvovirus 2a |
| >MT179785.1 | Beijing. China | Canine parvovirus |
| >MT179784.1 | Beijing. China | Canine parvovirus |
| >MT179783.1 | Beijing. China | Canine parvovirus |
| >MT179782.1 | Beijing. China | Canine parvovirus |
| >MT179781.1 | Beijing. China | Canine parvovirus |
| >MT179780.1 | Beijing. China | Canine parvovirus |
| >MT179779.1 | Beijing. China | Canine parvovirus |
| >MT179778.1 | Beijing. China | Canine parvovirus |
| >MT179777.1 | Beijing. China | Canine parvovirus |
| >MT179776.1 | Beijing. China | Canine parvovirus |
| >MT179775.1 | Beijing. China | Canine parvovirus |
| >MT179774.1 | Beijing. China | Canine parvovirus |
| >MT179773.1 | Beijing. China | Canine parvovirus |
| >MT179772.1 | Beijing. China | Canine parvovirus |
| >MT179771.1 | Beijing. China | Canine parvovirus |
| >MT179770.1 | Beijing. China | Canine parvovirus |
| >MT179769.1 | Beijing. China | Canine parvovirus |
| >MT179768.1 | Beijing. China | Canine parvovirus |
| >MT179767.1 | Beijing. China | Canine parvovirus |
| >MT179766.1 | Beijing. China | Canine parvovirus |
| >EU483517.1 | Hangzhou. Zhejiang. China | Canine parvovirus |
| >EU483516.1 | Hangzhou. Zhejiang. China | Canine parvovirus |
| >EU483515.1 | Hangzhou. Zhejiang. China | Canine parvovirus |
| >EU483514.1 | Hangzhou. Zhejiang. China | Canine parvovirus |
| >EU483513.1 | Hangzhou. Zhejiang. China | Canine parvovirus |
| >EU483512.1 | Hangzhou. Zhejiang. China | Canine parvovirus |
| >EU483511.1 | Hangzhou. Zhejiang. China | Canine parvovirus |
| >EU483510.1 | Hangzhou. Zhejiang. China | Canine parvovirus |
| >EU483509.1 | China | Canine parvovirus |
| >MN114239.1 | Yichun City, Jiangxi | Canine parvovirus |
| >MK208973.1 | Changchun, Jilin | Canine parvovirus 2 |
| >MK208972.1 | Changchun, Jilin | Canine parvovirus 2 |
| >MK208971.1 | Changchun, Jilin | Canine parvovirus 2 |
| >KY968643.1 | NanJing, JiangSu | Canine parvovirus |
| >KY968642.1 | NanJing, JiangSu | Canine parvovirus |
| >KY937675.1 | NanJing, JiangSu | Canine parvovirus |
| >KY937674.1 | NanJing, JiangSu | Canine parvovirus |
| >KY937673.1 | NanJing, JiangSu | Canine parvovirus |
| >KY937672.1 | NanJing, JiangSu | Canine parvovirus |
| >KY937671.1 | NanJing, JiangSu | Canine parvovirus |
| >KY937670.1 | NanJing, JiangSu | Canine parvovirus |
| >KY937669.1 | NanJing, JiangSu | Canine parvovirus |
| >KY937668.1 | NanJing, JiangSu | Canine parvovirus |
| >KY937667.1 | NanJing, JiangSu | Canine parvovirus |
| >KY937666.1 | NanJing, JiangSu | Canine parvovirus |
| >KY937665.1 | NanJing, JiangSu | Canine parvovirus |
| >KY937664.1 | NanJing, JiangSu | Canine parvovirus |
| >KY937663.1 | NanJing, JiangSu | Canine parvovirus |
| >KY937662.1 | NanJing, JiangSu | Canine parvovirus |
| >KY937661.1 | NanJing, JiangSu | Canine parvovirus |
| >KY937660.1 | NanJing, JiangSu | Canine parvovirus |
| >KY937659.1 | NanJing, JiangSu | Canine parvovirus |
| >KY937658.1 | NanJing, JiangSu | Canine parvovirus |
| >KY937657.1 | NanJing, JiangSu | Canine parvovirus |
| >KY937656.1 | NanJing, JiangSu | Canine parvovirus |
| >KY937655.1 | NanJing, JiangSu | Canine parvovirus |
| >KY937654.1 | NanJing, JiangSu | Canine parvovirus |
| >KY937653.1 | NanJing, JiangSu | Canine parvovirus |
| >KY937652.1 | NanJing, JiangSu | Canine parvovirus |
| >KY937651.1 | NanJing, JiangSu | Canine parvovirus |
| >KY937650.1 | NanJing, JiangSu | Canine parvovirus |
| >KY937649.1 | NanJing, JiangSu | Canine parvovirus |
| >KY937648.1 | NanJing, JiangSu | Canine parvovirus |
| >KY937646.1 | NanJing, JiangSu | Canine parvovirus |
| >KY937645.1 | NanJing, JiangSu | Canine parvovirus |
| >KY937644.1 | NanJing, JiangSu | Canine parvovirus |
| >KY937643.1 | NanJing, JiangSu | Canine parvovirus |
| >KY937642.1 | NanJing, JiangSu | Canine parvovirus |
| >KY937641.1 | NanJing, JiangSu | Canine parvovirus |
| >KY937640.1 | NanJing, JiangSu | Canine parvovirus |
| >KY937639.1 | NanJing, JiangSu | Canine parvovirus |
| >KY937638.1 | NanJing, JiangSu | Canine parvovirus |
| >KY937637.1 | NanJing, JiangSu | Canine parvovirus |
| >KY922910.1 | NanJing, JiangSu | Canine parvovirus |
| >KY922909.1 | NanJing, JiangSu | Canine parvovirus |
| >KY922908.1 | NanJing, JiangSu | Canine parvovirus |
| >KY922907.1 | NanJing, JiangSu | Canine parvovirus |
| >KY922906.1 | NanJing, JiangSu | Canine parvovirus |
| >KY922905.1 | NanJing, JiangSu | Canine parvovirus |
| >KP260509.1 | Changchun, Jilin | Canine parvovirus 2c |
| >JX048607.1 | Chiayi City, Taiwan | Canine parvovirus |
| >JX048606.1 | Chiayi City, Taiwan | Canine parvovirus |
| >JX048605.1 | Chiayi City, Taiwan | Canine parvovirus |
| >MZ223471.1 | India | Canine parvovirus |
| >GQ502462.1 | Tai`an, Shandong | Canine parvovirus |
| >GU362935.1 | Italy | cats |
| >GU362934.1 | Italy | cats |
| >GU362933.1 | Italy | cats |
| >GU362932.1 | Italy | cats |
| >FJ869126.1 | Thailand | Canine parvovirus |
| >FJ869124.1 | Thailand | Canine parvovirus |
| >FJ869123.1 | Thailand | Canine parvovirus |
| >JX121627.1 | Changchun, Jilin | Canine parvovirus |
| >JX121626.1 | Changchun, Jilin | Canine parvovirus |
| >JX121625.1 | Changchun, Jilin | Canine parvovirus |
| >JX121624.1 | Changchun, Jilin | Canine parvovirus |
| >JX121623.1 | Changchun, Jilin | Canine parvovirus |
| >JX120178.1 | Zhaoqing, Guangdong | Canine parvovirus |
| >GU212792.1 | Thailand | Canine parvovirus 2b |
| >GU212791.1 | Thailand | Canine parvovirus 2 |
| >GU212790.1 | Thailand | Canine parvovirus 2 |
| >GQ379049.1 | Thailand | Canine parvovirus |
| >GQ379048.1 | Thailand | Canine parvovirus |
| >GQ379047.1 | Thailand | Canine parvovirus |
| >GQ379046.1 | Thailand | Canine parvovirus |
| >GQ379045.1 | Thailand | Canine parvovirus |
| >GQ379044.1 | Thailand | Canine parvovirus |
| >GQ379043.1 | Thailand | Canine parvovirus |
| >GQ379042.1 | Thailand | Canine parvovirus |
| >MT952859.1 | Turkey | Canine parvovirus |
| >MT952858.1 | Turkey | Canine parvovirus |
| >MT952857.1 | Turkey | Canine parvovirus |
| >MT952856.1 | Turkey | Canine parvovirus |
| >MT952855.1 | Turkey | Canine parvovirus |
| >MW883497.1 | United Kingdom | Canine parvovirus 2 |
| >MW883496.1 | United Kingdom | Canine parvovirus 2 |
| >MW883495.1 | United Kingdom | Canine parvovirus 2 |
| >MW883494.1 | United Kingdom | Canine parvovirus 2 |
| >MW883493.1 | United Kingdom | Canine parvovirus 2 |
| >MW883492.1 | United Kingdom | Canine parvovirus 2 |
| >MW883491.1 | United Kingdom | Canine parvovirus 2 |
| >MW883490.1 | United Kingdom | Canine parvovirus 2 |
| >MW883489.1 | United Kingdom | Canine parvovirus 2 |
| >MW883488.1 | United Kingdom | Canine parvovirus 2 |
| >MW883487.1 | United Kingdom | Canine parvovirus 2 |
| >MW883486.1 | United Kingdom | Canine parvovirus 2 |
| >MN119620.1 | Nanyang, Henan | Canine parvovirus |
| >MN119619.1 | Nanyang, Henan | Canine parvovirus |
| >MN119618.1 | Nanyang, Henan | Canine parvovirus |
| >MN119617.1 | Nanyang, Henan | Canine parvovirus |
| >MN119616.1 | Nanyang, Henan | Canine parvovirus |
| >MN119615.1 | Nanyang, Henan | Canine parvovirus |
| >MN119614.1 | Nanyang, Henan | Canine parvovirus |
| >MN119613.1 | Nanyang, Henan | Canine parvovirus |
| >MN119612.1 | Nanyang, Henan | Canine parvovirus |
| >MN119611.1 | Nanyang, Henan | Canine parvovirus |
| >MN119610.1 | Nanyang, Henan | Canine parvovirus |
| >MN119609.1 | Nanyang, Henan | Canine parvovirus |
| >MN119608.1 | Nanyang, Henan | Canine parvovirus |
| >MN119607.1 | Nanyang, Henan | Canine parvovirus |
| >MN119606.1 | Nanyang, Henan | Canine parvovirus |
| >MN119605.1 | Nanyang, Henan | Canine parvovirus |
| >MN119604.1 | Nanyang, Henan | Canine parvovirus |
| >MN119603.1 | Nanyang, Henan | Canine parvovirus |
| >MN119602.1 | Nanyang, Henan | Canine parvovirus |
| >MN119601.1 | Nanyang, Henan | Canine parvovirus |
| >MN119600.1 | Nanyang, Henan | Canine parvovirus |
| >MN119599.1 | Nanyang, Henan | Canine parvovirus |
| >MN119598.1 | Nanyang, Henan | Canine parvovirus |
| >MN119597.1 | Nanyang, Henan | Canine parvovirus |
| >MN119596.1 | Nanyang, Henan | Canine parvovirus |
| >MN119595.1 | Nanyang, Henan | Canine parvovirus |
| >MN119594.1 | Nanyang, Henan | Canine parvovirus |
| >MN119593.1 | Nanyang, Henan | Canine parvovirus |
| >MN119592.1 | Nanyang, Henan | Canine parvovirus |
| >MN119591.1 | Nanyang, Henan | Canine parvovirus |
| >MN119590.1 | Nanyang, Henan | Canine parvovirus |
| >MN119589.1 | Nanyang, Henan | Canine parvovirus |
| >MN119588.1 | Nanyang, Henan | Canine parvovirus |
| >MN119587.1 | Nanyang, Henan | Canine parvovirus |
| >MN119586.1 | Nanyang, Henan | Canine parvovirus |
| >MN119585.1 | Nanyang, Henan | Canine parvovirus |
| >MN119584.1 | Nanyang, Henan | Canine parvovirus |
| >MN119583.1 | Nanyang, Henan | Canine parvovirus |
| >MN119582.1 | Nanyang, Henan | Canine parvovirus |
| >MN119581.1 | Nanyang, Henan | Canine parvovirus |
| >MN119580.1 | Nanyang, Henan | Canine parvovirus |
| >MN119579.1 | Nanyang, Henan | Canine parvovirus |
| >MN119578.1 | Nanyang, Henan | Canine parvovirus |
| >MN119577.1 | Nanyang, Henan | Canine parvovirus |
| >MN119576.1 | Nanyang, Henan | Canine parvovirus |
| >MN119575.1 | Nanyang, Henan | Canine parvovirus |
| >MN119574.1 | Nanyang, Henan | Canine parvovirus |
| >MN119573.1 | Nanyang, Henan | Canine parvovirus |
| >MN119572.1 | Nanyang, Henan | Canine parvovirus |
| >MN119571.1 | Nanyang, Henan | Canine parvovirus |
| >MN119570.1 | Nanyang, Henan | Canine parvovirus |
| >MN119569.1 | Nanyang, Henan | Canine parvovirus |
| >MN119568.1 | Nanyang, Henan | Canine parvovirus |
| >MN119567.1 | Nanyang, Henan | Canine parvovirus |
| >MN119566.1 | Nanyang, Henan | Canine parvovirus |
| >MN119565.1 | Nanyang, Henan | Canine parvovirus |
| >MN119564.1 | Nanyang, Henan | Canine parvovirus |
| >MN119563.1 | Nanyang, Henan | Canine parvovirus |
| >MN119562.1 | Nanyang, Henan | Canine parvovirus |
| >MN119561.1 | Nanyang, Henan | Canine parvovirus |
| >MN119560.1 | Nanyang, Henan | Canine parvovirus |
| >MK518021.1 | Nanyang, Henan | Canine parvovirus |
| >MK518020.1 | Nanyang, Henan | Canine parvovirus |
| >MK518019.1 | Nanyang, Henan | Canine parvovirus |
| >MK518018.1 | Nanyang, Henan | Canine parvovirus |
| >MK518017.1 | Nanyang, Henan | Canine parvovirus |
| >MK518016.1 | Nanyang, Henan | Canine parvovirus |
| >MK518015.1 | Nanyang, Henan | Canine parvovirus |
| >MK518014.1 | Nanyang, Henan | Canine parvovirus |
| >MK518013.1 | Nanyang, Henan | Canine parvovirus |
| >MK518012.1 | Nanyang, Henan | Canine parvovirus |
| >MK518011.1 | Nanyang, Henan | Canine parvovirus |
| >MK518010.1 | Nanyang, Henan | Canine parvovirus |
| >MK518009.1 | Nanyang, Henan | Canine parvovirus |
| >MK518008.1 | Nanyang, Henan | Canine parvovirus |
| >MK518007.1 | Nanyang, Henan | Canine parvovirus |
| >MK518006.1 | Nanyang, Henan | Canine parvovirus |
| >MK518005.1 | Nanyang, Henan | Canine parvovirus |
| >MK518004.1 | Nanyang, Henan | Canine parvovirus |
| >MK518003.1 | Nanyang, Henan | Canine parvovirus |
| >MK518002.1 | Nanyang, Henan | Canine parvovirus |
| >MK518001.1 | Nanyang, Henan | Canine parvovirus |
| >MK518000.1 | Nanyang, Henan | Canine parvovirus |
| >MK517999.1 | Nanyang, Henan | Canine parvovirus |
| >MK517998.1 | Nanyang, Henan | Canine parvovirus |
| >MK517997.1 | Nanyang, Henan | Canine parvovirus |
| >MK517996.1 | Nanyang, Henan | Canine parvovirus |
| >MK517995.1 | Nanyang, Henan | Canine parvovirus |
| >MK517994.1 | Nanyang, Henan | Canine parvovirus |
| >MK517993.1 | Nanyang, Henan | Canine parvovirus |
| >MK517992.1 | Nanyang, Henan | Canine parvovirus |
| >MK517991.1 | Nanyang, Henan | Canine parvovirus |
| >MK517990.1 | Nanyang, Henan | Canine parvovirus |
| >MK517989.1 | Nanyang, Henan | Canine parvovirus |
| >MK517988.1 | Nanyang, Henan | Canine parvovirus |
| >MK517987.1 | Nanyang, Henan | Canine parvovirus |
| >MK517986.1 | Nanyang, Henan | Canine parvovirus |
| >MK517985.1 | Nanyang, Henan | Canine parvovirus |
| >MK517984.1 | Nanyang, Henan | Canine parvovirus |
| >MK517983.1 | Nanyang, Henan | Canine parvovirus |
| >MK517982.1 | Nanyang, Henan | Canine parvovirus |
| >MK517981.1 | Nanyang, Henan | Canine parvovirus |
| >MK517980.1 | Nanyang, Henan | Canine parvovirus |
| >MK517979.1 | Nanyang, Henan | Canine parvovirus |
| >MK517978.1 | Nanyang, Henan | Canine parvovirus |
| >MK517977.1 | Nanyang, Henan | Canine parvovirus |
| >MK517976.1 | Nanyang, Henan | Canine parvovirus |
| >MK517975.1 | Nanyang, Henan | Canine parvovirus |
| >MK517974.1 | Nanyang, Henan | Canine parvovirus |
| >MK517973.1 | Nanyang, Henan | Canine parvovirus |
| >MK517972.1 | Nanyang, Henan | Canine parvovirus |
| >MK517971.1 | Nanyang, Henan | Canine parvovirus |
| >MK517970.1 | Nanyang, Henan | Canine parvovirus |
| >MK517969.1 | Nanyang, Henan | Canine parvovirus |
| >MK517968.1 | Nanyang, Henan | Canine parvovirus |
| >MK517967.1 | Nanyang, Henan | Canine parvovirus |
| >MK517966.1 | Nanyang, Henan | Canine parvovirus |
| >AY742955.1 | USA | Canine parvovirus |
| >AY742953.1 | USA | Canine parvovirus |
| >AY742951.1 | USA | Canine parvovirus |
| >KU244254.1 | Taiwan | Canine parvovirus 2c |
| >U72698.1 | Taipei, Taiwan | Canine parvovirus |
| >U72697.1 | Taipei, Taiwan | Canine parvovirus |
| >U72696.1 | Taipei, Taiwan | Canine parvovirus |
| >U72695.1 | Taipei, Taiwan | Canine parvovirus |
| >KF803643.1 | Beijing | Canine parvovirus |
| >KF803642.1 | Beijing | Canine parvovirus |
| >KF803641.1 | Beijing | Canine parvovirus |
| >KF803640.1 | Beijing | Canine parvovirus |
| >KT156837.1 | Daqing, Heilongjiang | Canine parvovirus |
| >KT156836.1 | Daqing, Heilongjiang | Canine parvovirus |
| >KT156835.1 | Daqing, Heilongjiang | Canine parvovirus |
| >KT156834.1 | Daqing, Heilongjiang | Canine parvovirus |
| >KT156833.1 | Daqing, Heilongjiang | Canine parvovirus |
| >KT156832.1 | Daqing, Heilongjiang | Canine parvovirus |
| >KT156831.1 | Daqing, Heilongjiang | Canine parvovirus |
| >KT156830.1 | Daqing, Heilongjiang | Canine parvovirus |
| >KT156829.1 | Daqing, Heilongjiang | Canine parvovirus |
| >KT156828.1 | Daqing, Heilongjiang | Canine parvovirus |
| >KT156827.1 | Daqing, Heilongjiang | Canine parvovirus |
| >KT156826.1 | Daqing, Heilongjiang | Canine parvovirus |
| >KT156825.1 | Daqing, Heilongjiang | Canine parvovirus |
| >GU392244.1 | Changchun, Jilin | Canine parvovirus 2 |
| >GU392243.1 | Changchun, Jilin | Canine parvovirus 2 |
| >GU392242.1 | Changchun, Jilin | Canine parvovirus 2 |
| >GU392241.1 | Changchun, Jilin | Canine parvovirus 2 |
| >GU392240.1 | Changchun, Jilin | Canine parvovirus 2 |
| >GU392239.1 | Changchun, Jilin | Canine parvovirus 2 |
| >JX411926.1 | Portugal | Canine parvovirus |
| >JF422105.2 | Portugal | Feline parvovirus |
| >JF906788.1 | Uruguay | Canine parvovirus |
| >GQ865519.1 | Greece | Canine parvovirus 2c |
| >GQ865518.1 | Greece | Canine parvovirus 2c |
| >HQ025913.1 | Italy | Canine parvovirus 2c |
| >GU569948.1 | Changchun, Jilin | Canine parvovirus 2a |
| >GU569947.1 | Changchun, Jilin | Canine parvovirus 2a |
| >GU569946.1 | Changchun, Jilin | Canine parvovirus 2a |
| >GU569945.1 | Changchun, Jilin | Canine parvovirus 2a |
| >GU569944.1 | Changchun, Jilin | Canine parvovirus 2a |
| >GU569943.1 | Changchun, Jilin | Canine parvovirus 2a |
| >GU569942.1 | Changchun, Jilin | Canine parvovirus 2a |
| >GU569941.1 | Changchun, Jilin | Canine parvovirus 2a |
| >GU569940.1 | Changchun, Jilin | Canine parvovirus 2a |
| >GU569939.1 | Changchun, Jilin | Canine parvovirus 2a |
| >GU569938.1 | Changchun, Jilin | Canine parvovirus 2a |
| >GU569937.1 | Changchun, Jilin | Canine parvovirus 2a |
| >GU569936.1 | Changchun, Jilin | Canine parvovirus 2a |
| >FJ222821.1 | Italy | Canine parvovirus 2c |
| >FJ005259.1 | Italy | Canine parvovirus 2a |
| >FJ005258.1 | Italy | Canine parvovirus 2a |
| >FJ005257.1 | Italy | Canine parvovirus 2a |
| >FJ005256.1 | Italy | Canine parvovirus 2a |
| >FJ005255.1 | Italy | Canine parvovirus 2a |
| >FJ005254.1 | Italy | Canine parvovirus 2a |
| >FJ005253.1 | Italy | Canine parvovirus 2a |
| >FJ005252.1 | Italy | Canine parvovirus 2a |
| >FJ005251.1 | Italy | Canine parvovirus 2a |
| >FJ005250.1 | Italy | Canine parvovirus 2a |
| >FJ222824.1 | Italy | Canine parvovirus 2 |
| >FJ222823.1 | Italy | Canine parvovirus 2b |
| >FJ005265.1 | Italy | Canine parvovirus 2b |
| >FJ005249.1 | Italy | Canine parvovirus 2c |
| >FJ005248.1 | Italy | Canine parvovirus 2c |
| >FJ005247.1 | Italy | Canine parvovirus 2c |
| >FJ005246.1 | Italy | Canine parvovirus 2c |
| >FJ005245.1 | Italy | Canine parvovirus 2c |
| >FJ005244.1 | Italy | Canine parvovirus 2c |
| >FJ005243.1 | Italy | Canine parvovirus 2c |
| >FJ005242.1 | Italy | Canine parvovirus 2c |
| >FJ005241.1 | Italy | Canine parvovirus 2c |
| >FJ005240.1 | Italy | Canine parvovirus 2c |
| >FJ005239.1 | Italy | Canine parvovirus 2c |
| >FJ222822.1 | Italy | Canine parvovirus 2b |
| >FJ005238.1 | Italy | Canine parvovirus 2c |
| >FJ005237.1 | Italy | Canine parvovirus 2c |
| >FJ005264.1 | Italy | Canine parvovirus 2b |
| >FJ005263.1 | Italy | Canine parvovirus 2b |
| >FJ005236.1 | Italy | Canine parvovirus 2c |
| >FJ005235.1 | Italy | Canine parvovirus 2c |
| >FJ005234.1 | Italy | Canine parvovirus 2c |
| >FJ005233.1 | Italy | Canine parvovirus 2c |
| >FJ005262.1 | Italy | Canine parvovirus 2b |
| >FJ005232.1 | Italy | Canine parvovirus 2c |
| >FJ005231.1 | Italy | Canine parvovirus 2c |
| >FJ005261.1 | Italy | Canine parvovirus 2b |
| >FJ005260.1 | Italy | Canine parvovirus 3b |
| >FJ005230.1 | Italy | Canine parvovirus 2c |
| >FJ005229.1 | Italy | Canine parvovirus 2c |
| >FJ005228.1 | Italy | Canine parvovirus 2c |
| >FJ005227.1 | Italy | Canine parvovirus 2c |
| >FJ005226.1 | Italy | Canine parvovirus 2c |
| >FJ005225.1 | Italy | Canine parvovirus 2c |
| >FJ005224.1 | Italy | Canine parvovirus 2c |
| >FJ005223.1 | Italy | Canine parvovirus 2c |
| >FJ005222.1 | Italy | Canine parvovirus 2c |
| >FJ005221.1 | Italy | Canine parvovirus 2c |
| >FJ005220.1 | Italy | Canine parvovirus 2c |
| >FJ005219.1 | Italy | Canine parvovirus 2c |
| >FJ005218.1 | Italy | Canine parvovirus 2c |
| >FJ005217.1 | Italy | Canine parvovirus 2c |
| >FJ005216.1 | Italy | Canine parvovirus 2c |
| >FJ005215.1 | Italy | Canine parvovirus 2c |
| >FJ005214.1 | Italy | Canine parvovirus 2c |
| >FJ005213.1 | Italy | Canine parvovirus 2c |
| >FJ005212.1 | Italy | Canine parvovirus 2c |
| >FJ005211.1 | Italy | Canine parvovirus 2c |
| >FJ005210.1 | Italy | Canine parvovirus 2c |
| >FJ005209.1 | Italy | Canine parvovirus 2c |
| >FJ005208.1 | Italy | Canine parvovirus 2c |
| >FJ005207.1 | Italy | Canine parvovirus 2c |
| >FJ005206.1 | Italy | Canine parvovirus 2c |
| >FJ005205.1 | Italy | Canine parvovirus 2c |
| >FJ005204.1 | Italy | Canine parvovirus 2c |
| >FJ005203.1 | Italy | Canine parvovirus 2c |
| >FJ005202.1 | Italy | Canine parvovirus 2c |
| >FJ005201.1 | Italy | Canine parvovirus 2c |
| >FJ005200.1 | Italy | Canine parvovirus 2c |
| >FJ005199.1 | Italy | Canine parvovirus 2c |
| >FJ005198.1 | Italy | Canine parvovirus 2c |
| >FJ005197.1 | Italy | Canine parvovirus 2c |
| >FJ005196.1 | Italy | Canine parvovirus 2c |
| >FJ005195.1 | Italy | Canine parvovirus 2c |
| >DQ177497.1 | Guang Zhou, Guang Dong | Canine parvovirus |
| >OP779673.1 | Chengdu, Sichuan | Canine parvovirus 2c |
| >OP779672.1 | Chengdu, Sichuan | Canine parvovirus 2c |
| >OP779671.1 | Chengdu, Sichuan | Canine parvovirus 2c |
| >OP779670.1 | Chengdu, Sichuan | Canine parvovirus 2c |
| >OP779669.1 | Chengdu, Sichuan | Canine parvovirus 2c |
| >OP779668.1 | Chengdu, Sichuan | Canine parvovirus 2c |
| >OP779667.1 | Chengdu, Sichuan | Canine parvovirus 2c |
| >OP779666.1 | Chengdu, Sichuan | Canine parvovirus 2c |
| >OP779665.1 | Chengdu, Sichuan | Canine parvovirus 2c |
| >OP779664.1 | Chengdu, Sichuan | Canine parvovirus 2c |
| >OP779663.1 | Chengdu, Sichuan | Canine parvovirus 2c |
| >OP779662.1 | Chengdu, Sichuan | Canine parvovirus 2c |
| >OP779661.1 | Chengdu, Sichuan | Canine parvovirus 2c |
| >OP779660.1 | Chengdu, Sichuan | Canine parvovirus 2c |
| >OP779659.1 | Chengdu, Sichuan | Canine parvovirus 2c |
| >OP779658.1 | Chengdu, Sichuan | Canine parvovirus 2c |
| >OP779657.1 | Chengdu, Sichuan | Canine parvovirus 2c |
| >OP779656.1 | Chengdu, Sichuan | Canine parvovirus 2c |
| >OP779655.1 | Chengdu, Sichuan | Canine parvovirus 2c |
| >OP779654.1 | Chengdu, Sichuan | Canine parvovirus 2c |
| >OP779653.1 | Chengdu, Sichuan | Canine parvovirus 2c |
| >OP779652.1 | Chengdu, Sichuan | Canine parvovirus 2c |
| >OP779651.1 | Chengdu, Sichuan | Canine parvovirus 2c |
| >OP779650.1 | Chengdu, Sichuan | Canine parvovirus 2c |
| >OP779649.1 | Chengdu, Sichuan | Canine parvovirus 2c |
| >OP779648.1 | Chengdu, Sichuan | Canine parvovirus 2c |
| >OP779647.1 | Chengdu, Sichuan | Canine parvovirus 2c |
| >OP779646.1 | Chengdu, Sichuan | Canine parvovirus 2c |
| >OP779645.1 | Chengdu, Sichuan | Canine parvovirus 2c |
| >OP779644.1 | Chengdu, Sichuan | Canine parvovirus 2c |
| >OP779643.1 | Chengdu, Sichuan | Canine parvovirus 2c |
| >OP779642.1 | Chengdu, Sichuan | Canine parvovirus 2c |
| >OP779641.1 | Chengdu, Sichuan | Canine parvovirus 2c |
| >OP779640.1 | Chengdu, Sichuan | Canine parvovirus 2c |
| >OP779639.1 | Chengdu, Sichuan | Canine parvovirus 2c |
| >OP779638.1 | Chengdu, Sichuan | Canine parvovirus 2c |
| >OP779637.1 | Chengdu, Sichuan | Canine parvovirus 2c |
| >OP779636.1 | Chengdu, Sichuan | Canine parvovirus 2c |
| >OP779635.1 | Chengdu, Sichuan | Canine parvovirus 2c |
| >OP779634.1 | Chengdu, Sichuan | Canine parvovirus 2c |
| >OP779633.1 | Chengdu, Sichuan | Canine parvovirus 2c |
| >OP779632.1 | Chengdu, Sichuan | Canine parvovirus 2c |
| >MZ857186.1 | Chengdu, Sichuan | Canine parvovirus 2c |
| >MZ857185.1 | Chengdu, Sichuan | Canine parvovirus 2c |
| >MZ857184.1 | Chengdu, Sichuan | Canine parvovirus 2c |
| >MZ857183.1 | Chengdu, Sichuan | Canine parvovirus 2c |
| >MZ857182.1 | Chengdu, Sichuan | Canine parvovirus 2c |
| >MZ857181.1 | Chengdu, Sichuan | Canine parvovirus 2c |
| >MZ857180.1 | Chengdu, Sichuan | Canine parvovirus |
| >OP186113.1 | South Korea | Canine parvovirus |
| >OM212011.1 | Yanji, Jilin | Feline parvovirus |
| >MZ391101.1 | Turkey | Canine parvovirus |
| >MZ391100.1 | Turkey | Canine parvovirus |
| >MZ391099.1 | Turkey | Canine parvovirus |
| >MZ391098.1 | Turkey | Canine parvovirus |
| >OK571394.1 | Shanghai | Canine parvovirus |
| >OK571393.1 | Shanghai | Canine parvovirus |
| >OK571392.1 | Shanghai | Canine parvovirus |
| >MW728982.1 | Beijing | Canine parvovirus |
| >MW728981.1 | Beijing | Canine parvovirus |
| >MW728980.1 | Beijing | Canine parvovirus |
| >MW728979.1 | Beijing | Canine parvovirus |
| >MW728978.1 | Beijing | Canine parvovirus |
| >MW728977.1 | Beijing | Canine parvovirus |
| >MW728976.1 | Beijing | Canine parvovirus |
| >MW728975.1 | Beijing | Canine parvovirus |
| >MW728974.1 | Beijing | Canine parvovirus |
| >MW728973.1 | Beijing | Canine parvovirus |
| >MW728972.1 | Beijing | Canine parvovirus |
| >MW728971.1 | Beijing | Canine parvovirus |
| >MW728970.1 | Beijing | Canine parvovirus |
| >MW728969.1 | Beijing | Canine parvovirus |
| >MW728968.1 | Beijing | Canine parvovirus |
| >MW728967.1 | Beijing | Canine parvovirus |
| >MW728966.1 | Beijing | Canine parvovirus |
| >MW728965.1 | Beijing | Canine parvovirus |
| >MW728964.1 | Beijing | Canine parvovirus |
| >MW728963.1 | Beijing | Canine parvovirus |
| >MW728962.1 | Beijing | Canine parvovirus |
| >MW728961.1 | Beijing | Canine parvovirus |
| >MW728960.1 | Beijing | Canine parvovirus |
| >MW728959.1 | Beijing | Canine parvovirus |
| >MW728958.1 | Beijing | Canine parvovirus |
| >MW728957.1 | Beijing | Canine parvovirus |
| >MW728956.1 | Beijing | Canine parvovirus |
| >MW728955.1 | Beijing | Canine parvovirus |
| >MW728954.1 | Beijing | Canine parvovirus |
| >MW728953.1 | Beijing | Canine parvovirus |
| >MT996061.1 | Italy | Canine parvovirus 2 |
| >MT996058.1 | Italy | Canine parvovirus 2 |
| >MT996054.1 | Italy | Canine parvovirus 2 |
| >MT996053.1 | Italy | Canine parvovirus 2 |
| >MT996052.1 | Italy | Canine parvovirus 2 |
| >MT996051.1 | Italy | Canine parvovirus 2 |
| >MT996050.1 | Italy | Canine parvovirus 2 |
| >MT996048.1 | Italy | Canine parvovirus 2 |
| >MT996047.1 | Italy | Canine parvovirus 2 |
| >MT996046.1 | Italy | Canine parvovirus 2 |
| >MT996044.1 | Italy | Canine parvovirus 2 |
| >MT996042.1 | Italy | Canine parvovirus 2 |
| >MT996041.1 | Italy | Canine parvovirus 2 |
| >MT996039.1 | Italy | Canine parvovirus 2 |
| >MT996038.1 | Italy | Canine parvovirus 2 |
| >MT996037.1 | Italy | Canine parvovirus 2 |
| >MT996036.1 | Italy | Canine parvovirus 2 |
| >MT996034.1 | Italy | Canine parvovirus 2 |
| >MT996032.1 | Italy | Canine parvovirus 2 |
| >MT996031.1 | Italy | Canine parvovirus 2 |
| >MT996030.1 | Italy | Canine parvovirus 2 |
| >MT996028.1 | Italy | Canine parvovirus 2 |
| >MT996027.1 | Italy | Canine parvovirus 2 |
| >MT996026.1 | Italy | Canine parvovirus 2 |
| >MT996025.1 | Italy | Canine parvovirus 2 |
| >MT996023.1 | Italy | Canine parvovirus 2 |
| >MT996021.1 | Italy | Canine parvovirus 2 |
| >MT996020.1 | Italy | Canine parvovirus 2 |
| >MT996019.1 | Italy | Canine parvovirus 2 |
| >MT996018.1 | Italy | Canine parvovirus 2 |
| >MT996017.1 | Italy | Canine parvovirus 2 |
| >OK346434.1 | India | Canine parvovirus 2 |
| >OK346433.1 | India | Canine parvovirus 2 |
| >OK346432.1 | India | Canine parvovirus 2 |
| >OK346431.1 | India | Canine parvovirus 2 |
| >OK346430.1 | India | Canine parvovirus 2 |
| >MT860114.1 | Iran | Canine parvovirus |
| >MT860113.1 | Iran | Canine parvovirus |
| >MT860112.1 | Iran | Canine parvovirus |
| >MT860111.1 | Iran | Canine parvovirus |
| >MT860110.1 | Iran | Canine parvovirus |
| >MT860109.1 | Iran | Canine parvovirus |
| >MT860108.1 | Iran | Canine parvovirus |
| >MT860107.1 | Iran | Canine parvovirus |
| >MT860106.1 | Iran | Canine parvovirus |
| >MT860105.1 | Iran | Canine parvovirus |
| >MT860104.1 | Iran | Canine parvovirus |
| >MT860103.1 | Iran | Canine parvovirus |
| >MT860102.1 | Iran | Canine parvovirus |
| >MT860101.1 | Iran | Canine parvovirus |
| >MT860100.1 | Iran | Canine parvovirus |
| >MT860099.1 | Iran | Canine parvovirus |
| >MT860098.1 | Iran | Canine parvovirus |
| >MT860097.1 | Iran | Canine parvovirus |
| >MT860096.1 | Iran | Canine parvovirus |
| >MT860095.1 | Iran | Canine parvovirus |
| >MT860094.1 | Iran | Canine parvovirus |
| >MT860093.1 | Iran | Canine parvovirus |
| >MT860092.1 | Iran | Canine parvovirus |
| >MT860091.1 | Iran | Canine parvovirus |
| >MT860090.1 | Iran | Canine parvovirus |
| >MT860089.1 | Iran | Canine parvovirus |
| >MT860088.1 | Iran | Canine parvovirus |
| >MT860087.1 | Iran | Canine parvovirus |
| >MT860086.1 | Iran | Canine parvovirus |
| >MT860085.1 | Iran | Canine parvovirus |
| >MT860084.1 | Iran | Canine parvovirus |
| >MT860083.1 | Iran | Canine parvovirus |
| >MT860082.1 | Iran | Canine parvovirus |
| >MT860081.1 | Iran | Canine parvovirus |
| >MT860080.1 | Iran | Canine parvovirus |
| >MT860079.1 | Iran | Canine parvovirus |
| >MT860078.1 | Iran | Canine parvovirus |
| >MT860077.1 | Iran | Canine parvovirus |
| >MT860076.1 | Iran | Canine parvovirus |
| >MT860075.1 | Iran | Canine parvovirus |
| >MT860074.1 | Iran | Canine parvovirus |
| >MT860073.1 | Iran | Canine parvovirus |
| >MT860072.1 | Iran | Canine parvovirus |
| >MT860071.1 | Iran | Canine parvovirus |
| >MT860070.1 | Iran | Canine parvovirus |
| >MT860069.1 | Iran | Canine parvovirus |
| >MT860068.1 | Iran | Canine parvovirus |
| >MT860067.1 | Iran | Canine parvovirus |
| >MT860066.1 | Iran | Canine parvovirus |
| >KY418607.1 | Guangzhou | Canine parvovirus |
| >LC216910.1 | Japan | Canine parvovirus 2c |
| >LC216909.1 | Japan | Canine parvovirus 2c |
| >LC216908.1 | Japan | Canine parvovirus 2c |
| >LC216907.1 | Japan | Canine parvovirus 2c |
| >LC216906.1 | Japan | Canine parvovirus 2c |
| >LC216905.1 | Japan | Canine parvovirus 2c |
| >LC216904.1 | Japan | Canine parvovirus 2c |
| >MG462710.1 | Kunming, Yunnan | Canine parvovirus |
| >GU392238.1 | Changchun, Jinlin | Canine parvovirus 2 |
| >GU392237.1 | Changchun, Jinlin | Canine parvovirus 2 |
| >GU392236.1 | Changchun, Jinlin | Canine parvovirus 2 |
| >JQ996155.1 | Ya'an, Sichuan | Canine parvovirus 2a |
| >JQ996154.1 | Ya'an, Sichuan | Canine parvovirus 2a |
| >JQ996153.1 | Ya'an, Sichuan | Canine parvovirus 2a |
| >JQ996152.1 | Ya'an, Sichuan | Canine parvovirus 2a |
| >JQ996151.1 | Ya'an, Sichuan | Canine parvovirus 2a |
| >HQ651237.1 | Ya'an, Sichuan | Canine parvovirus 2a |
| >AB262659.1 | Japan | Feline parvovirus |
| >MW659478.1 | Italy | Protoparvovirus carnivoran1 |
| >MW659477.1 | Italy | Protoparvovirus carnivoran2 |
| >MW659476.1 | Italy | Protoparvovirus carnivoran3 |
| >MW659475.1 | Italy | Protoparvovirus carnivoran4 |
| >MW659474.1 | Italy | Protoparvovirus carnivoran5 |
| >MW659473.1 | Italy | Protoparvovirus carnivoran6 |
| >MW659472.1 | Italy | Protoparvovirus carnivoran7 |
| >MW659471.1 | Italy | Protoparvovirus carnivoran8 |
| >MW659470.1 | Italy | Protoparvovirus carnivoran9 |
| >MW659469.1 | Italy | Protoparvovirus carnivoran10 |
| >KU248464.1 | Portugal | Feline panleukopenia virus |
| >KU248463.1 | Portugal | Feline panleukopenia virus |
| >KU248462.1 | Portugal | Feline panleukopenia virus |
| >KU248461.1 | Portugal | Feline panleukopenia virus |
| >KU248460.1 | Portugal | Feline panleukopenia virus |
| >KU248459.1 | Portugal | Feline panleukopenia virus |
| >KU248458.1 | Portugal | Feline panleukopenia virus |
| >KU248457.1 | Portugal | Feline panleukopenia virus |
| >KU248456.1 | Portugal | Feline panleukopenia virus |
| >KT240136.1 | Portugal | Feline panleukopenia virus |
| >KT240135.1 | Portugal | Feline panleukopenia virus |
| >KT240134.1 | Portugal | Feline panleukopenia virus |
| >KT240133.1 | Portugal | Feline panleukopenia virus |
| >KT240132.1 | Portugal | Feline panleukopenia virus |
| >KT240131.1 | Portugal | Feline panleukopenia virus |
| >KT240130.1 | Portugal | Feline panleukopenia virus |
| >KT240129.1 | Portugal | Feline panleukopenia virus |
| >KT240128.1 | Portugal | Feline panleukopenia virus |
| >JN867596.1 | USA | Feline panleukopenia virus |
| >JN867595.1 | USA | Feline panleukopenia virus |
| >JN867594.1 | USA | Feline panleukopenia virus |
| >JN867593.1 | USA | Feline panleukopenia virus |
| >EU145593.1 | Hungary | Feline panleukopenia virus |
| >DQ099431.1 | Changchun, Jilin | Feline panleukopenia virus |
| >DQ099430.1 | Changchun, Jilin | Feline panleukopenia virus |
| >DQ474238.1 | Changchun, Jilin | Feline panleukopenia virus |
| >DQ474237.1 | Changchun, Jilin | Feline panleukopenia virus |
| >DQ474236.1 | Changchun, Jilin | Feline panleukopenia virus |
| >DQ474235.1 | Changchun, Jilin | Feline panleukopenia virus |
| >MT078771.1 | India | Feline panleukopenia virus |
| >MT078770.1 | India | Feline panleukopenia virus |
| >MT078769.1 | India | Feline panleukopenia virus |
| >MT078768.1 | India | Feline panleukopenia virus |
| >MT078767.1 | India | Feline panleukopenia virus |
| >EU252147.1 | Korea | Feline panleukopenia virus |
| >EU252146.1 | Korea | Feline panleukopenia virus |
| >EU252145.1 | Korea | Feline panleukopenia virus |
| >HM015824.1 | Changchun, Jilin | Mink enteritis virus |
| >MZ322607.1 | Changchun, Jilin | Feline panleukopenia virus |
| >MT857286.1 | Taiwan | Feline panleukopenia virus |
| >MT857285.1 | Taiwan | Feline panleukopenia virus |
| >MT857284.1 | Taiwan | Feline panleukopenia virus |
| >MT857283.1 | Taiwan | Feline panleukopenia virus |
| >MT857282.1 | Taiwan | Feline panleukopenia virus |
| >MT857281.1 | Taiwan | Feline panleukopenia virus |
| >MT857280.1 | Taiwan | Feline panleukopenia virus |
| >MT857279.1 | Taiwan | Feline panleukopenia virus |
| >MT857278.1 | Taiwan | Feline panleukopenia virus |
| >MT857277.1 | Taiwan | Feline panleukopenia virus |
| >MT857276.1 | Taiwan | Feline panleukopenia virus |
| >MT857275.1 | Taiwan | Feline panleukopenia virus |
| >MT857274.1 | Taiwan | Feline panleukopenia virus |
| >MT857273.1 | Taiwan | Feline panleukopenia virus |
| >MT857272.1 | Taiwan | Feline panleukopenia virus |
| >MT857271.1 | Taiwan | Feline panleukopenia virus |
| >MT857270.1 | Taiwan | Feline panleukopenia virus |
| >MT857269.1 | Taiwan | Feline panleukopenia virus |
| >MT857268.1 | Taiwan | Feline panleukopenia virus |
| >MK348106.1 | Italy | Protoparvovirus carnivoran1 |
| >MK348105.1 | Italy | Protoparvovirus carnivoran1 |
| >MK348104.1 | Italy | Protoparvovirus carnivoran1 |
| >MK348103.1 | Italy | Protoparvovirus carnivoran1 |
| >MK348102.1 | Italy | Protoparvovirus carnivoran1 |
| >MK348101.1 | Italy | Protoparvovirus carnivoran1 |
| >MK348100.1 | Italy | Protoparvovirus carnivoran1 |
| >MK348099.1 | Italy | Protoparvovirus carnivoran1 |
| >MK348098.1 | Italy | Protoparvovirus carnivoran1 |
| >MK348097.1 | Italy | Protoparvovirus carnivoran1 |
| >MK348096.1 | Italy | Protoparvovirus carnivoran1 |
| >MK348095.1 | Italy | Protoparvovirus carnivoran1 |
| >MK348094.1 | Italy | Protoparvovirus carnivoran1 |
| >MK348093.1 | Italy | Protoparvovirus carnivoran1 |
| >MK348092.1 | Italy | Protoparvovirus carnivoran1 |
| >MK348091.1 | Italy | Protoparvovirus carnivoran1 |
| >MK348090.1 | Italy | Protoparvovirus carnivoran1 |
| >AF015223.1 | Taiwan | Feline panleukopenia virus |
| >OP153932.1 | South Korea | Feline panleukopenia virus |
| >OP153931.1 | South Korea | Feline panleukopenia virus |
| >OP153930.1 | South Korea | Feline panleukopenia virus |
| >OP153929.1 | South Korea | Feline panleukopenia virus |
| >OP153928.1 | South Korea | Feline panleukopenia virus |
| >OP153927.1 | South Korea | Feline panleukopenia virus |
| >OP153926.1 | South Korea | Feline panleukopenia virus |
| >OP153925.1 | South Korea | Feline panleukopenia virus |
| >MZ391097.1 | Turkey | Feline panleukopenia virus |
| >MZ391096.1 | Turkey | Feline panleukopenia virus |
| >MW495848.1 | Yangzhou, Jiangsu | Feline panleukopenia virus |
| >MW495847.1 | Yangzhou, Jiangsu | Feline panleukopenia virus |
| >MW495846.1 | Yangzhou, Jiangsu | Feline panleukopenia virus |
| >MW495845.1 | Yangzhou, Jiangsu | Feline panleukopenia virus |
| >MW495844.1 | Yangzhou, Jiangsu | Feline panleukopenia virus |
| >MW495843.1 | Yangzhou, Jiangsu | Feline panleukopenia virus |
| >MW495842.1 | Yangzhou, Jiangsu | Feline panleukopenia virus |
| >MW495841.1 | Yangzhou, Jiangsu | Feline panleukopenia virus |
| >MW495840.1 | Yangzhou, Jiangsu | Feline panleukopenia virus |
| >MW495839.1 | Yangzhou, Jiangsu | Feline panleukopenia virus |
| >MW495838.1 | Yangzhou, Jiangsu | Feline panleukopenia virus |
| >MW495837.1 | Yangzhou, Jiangsu | Feline panleukopenia virus |
| >MW495836.1 | Yangzhou, Jiangsu | Feline panleukopenia virus |
| >MW495835.1 | Yangzhou, Jiangsu | Feline panleukopenia virus |
| >MW495834.1 | Yangzhou, Jiangsu | Feline panleukopenia virus |
| >MW495833.1 | Yangzhou, Jiangsu | Feline panleukopenia virus |
| >MW495832.1 | Yangzhou, Jiangsu | Feline panleukopenia virus |
| >MW495831.1 | Yangzhou, Jiangsu | Feline panleukopenia virus |
| >MW495830.1 | Yangzhou, Jiangsu | Feline panleukopenia virus |
| >MW495829.1 | Yangzhou, Jiangsu | Feline panleukopenia virus |
| >MH669800.1 | Thailand | Feline panleukopenia virus |
| >KY012246.1 | Harbin, Heilongjiang | Mink enteritis virus |
| >KY012245.1 | Harbin, Heilongjiang | Mink enteritis virus |
| >KY083104.1 | Singapore | Protoparvovirus sp. |
| >KY083101.1 | Singapore | Protoparvovirus sp. |
| >KY083099.1 | Singapore | Protoparvovirus sp. |
| >KY083098.1 | Singapore | Protoparvovirus sp. |
| >KY083097.1 | Singapore | Protoparvovirus sp. |
| >KY083096.1 | Singapore | Protoparvovirus sp. |
| >KY083093.1 | Singapore | Protoparvovirus sp. |
| >KY083092.1 | Singapore | Protoparvovirus sp. |
| >KY083091.1 | Singapore | Protoparvovirus sp. |
| >KY083090.1 | Singapore | Protoparvovirus sp. |
| >KY083089.1 | Singapore | Protoparvovirus sp. |
| >GU392259.1 | Changchun, Jilin | Mink enteritis virus |
| >GU392258.1 | Changchun, Jilin | Mink enteritis virus |
| >GU392257.1 | Changchun, Jilin | Mink enteritis virus |
| >GU392256.1 | Changchun, Jilin | Mink enteritis virus |
| >GU392255.1 | Changchun, Jilin | Mink enteritis virus |
| >GU392254.1 | Changchun, Jilin | Mink enteritis virus |
| >GU392253.1 | Changchun, Jilin | Mink enteritis virus |
| >GU392252.1 | Changchun, Jilin | Mink enteritis virus |
| >GU392251.1 | Changchun, Jilin | Mink enteritis virus |
| >GU392250.1 | Changchun, Jilin | Mink enteritis virus |
| >GU392249.1 | Changchun, Jilin | Mink enteritis virus |
| >GU392248.1 | Changchun, Jilin | Mink enteritis virus |
| >GU392247.1 | Changchun, Jilin | Mink enteritis virus |
| >GU392246.1 | Changchun, Jilin | Mink enteritis virus |
| >GU392245.1 | Changchun, Jilin | Mink enteritis virus |
| >D78585.1 | Japan | Canine parvovirus |
| >EU170352.1 | Shihezi, Xinjiang | Canine parvovirus |
| >MT270597.1 | Beijing | Canine parvovirus |
| >MT270596.1 | Beijing | Canine parvovirus |
| >MT270595.1 | Beijing | Canine parvovirus |
| >MT270594.1 | Beijing | Canine parvovirus |
| >MT270593.1 | Beijing | Canine parvovirus |
| >MT270592.1 | Beijing | Canine parvovirus |
| >MT270591.1 | Beijing | Canine parvovirus |
| >MT270590.1 | Beijing | Canine parvovirus |
| >MT270589.1 | Beijing | Canine parvovirus |
| >MT270588.1 | Beijing | Canine parvovirus |
| >MT270587.1 | Beijing | Canine parvovirus |
| >MT270586.1 | Beijing | Canine parvovirus |
| >MT023794.1 | Changchun, Jilin | Canine parvovirus |
| >MH891837.1 | India | Canine parvovirus 2a |
| >MH891836.1 | India | Canine parvovirus 2a |
| >MH891835.1 | India | Canine parvovirus 2a |
| >KP071952.1 | India | Canine parvovirus 2a |
| >GU380305.1 | Changchun, Jilin | Canine parvovirus 2c |
| >GU380304.1 | Changchun, Jilin | Canine parvovirus 2a |
| >GU380303.1 | Changchun, Jilin | Canine parvovirus 2a |
| >GU380302.1 | Changchun, Jilin | Canine parvovirus 2a |
| >GU380301.1 | Changchun, Jilin | Canine parvovirus 2a |
| >GU380300.1 | Changchun, Jilin | Canine parvovirus 2a |
| >GU380299.1 | Changchun, Jilin | Canine parvovirus 2a |
| >GU380298.1 | Changchun, Jilin | Canine parvovirus 2a |
| >GQ857614.1 | Tai`an, Shandong | Canine parvovirus |
| >GQ857613.1 | Tai`an, Shandong | Canine parvovirus |
| >GQ857612.1 | Tai`an, Shandong | Canine parvovirus |
| >GQ857611.1 | Tai`an, Shandong | Canine parvovirus |
| >GQ857610.1 | Tai`an, Shandong | Canine parvovirus |
| >GQ857609.1 | Tai`an, Shandong | Canine parvovirus |
| >GQ857608.1 | Tai`an, Shandong | Canine parvovirus |
| >GQ857607.1 | Tai`an, Shandong | Canine parvovirus |
| >GQ857606.1 | Tai`an, Shandong | Canine parvovirus |
| >GQ857605.1 | Tai`an, Shandong | Canine parvovirus |
| >GQ857604.1 | Tai`an, Shandong | Canine parvovirus |
| >GQ857603.1 | Tai`an, Shandong | Canine parvovirus |
| >GQ857602.1 | Tai`an, Shandong | Canine parvovirus |
| >GQ857601.1 | Tai`an, Shandong | Canine parvovirus |
| >GQ857600.1 | Tai`an, Shandong | Canine parvovirus |
| >GQ857599.1 | Tai`an, Shandong | Canine parvovirus |
| >GQ857598.1 | Tai`an, Shandong | Canine parvovirus |
| >GQ857597.1 | Tai`an, Shandong | Canine parvovirus |
| >GQ857596.1 | Tai`an, Shandong | Canine parvovirus |
| >GQ857595.1 | Tai`an, Shandong | Feline parvovirus |
| >EF028071.1 | Ya'an, Sichuan | Canine parvovirus |
| >EF666067.2 | Beijing | Canine parvovirus |
| >EF666065.2 | Beijing | Canine parvovirus |
| >EF666069.1 | Beijing | Canine parvovirus |
| >EF666068.1 | Beijing | Canine parvovirus |
| >EF666066.1 | Beijing | Canine parvovirus |
| >EF666064.1 | Beijing | Canine parvovirus |
| >EF666063.1 | Beijing | Canine parvovirus |
| >EF666062.1 | Beijing | Canine parvovirus |
| >EF666061.1 | Beijing | Canine parvovirus |
| >EF666060.1 | Beijing | Canine parvovirus |
| >EF666059.1 | Beijing | Canine parvovirus |
| >EU697385.1 | Changchun, Jilin | Canine parvovirus |
| >EU145961.1 | Beijing | Canine parvovirus |
| >EU145960.1 | Beijing | Canine parvovirus |
| >EU145959.1 | Beijing | Canine parvovirus |
| >EU145958.1 | Beijing | Canine parvovirus |
| >EU145957.1 | Beijing | Canine parvovirus |
| >EU145956.1 | Beijing | Canine parvovirus |
| >EU145955.1 | Beijing | Canine parvovirus |
| >EU145954.1 | Beijing | Canine parvovirus |
| >EU145953.1 | Beijing | Canine parvovirus |
| >OP210302.1 | Viet Nam | Canine parvovirus |
| >OP210301.1 | Viet Nam | Canine parvovirus |
| >OM100702.1 | Egypt | Canine parvovirus 2 |
| >OM100701.1 | Egypt | Canine parvovirus 2 |
| >OM100700.1 | Egypt | Canine parvovirus 2 |
| >OM100699.1 | Egypt | Canine parvovirus 2 |
| >OM100698.1 | Egypt | Canine parvovirus 2 |
| >OM100697.1 | Egypt | Canine parvovirus 2 |
| >OM100696.1 | Egypt | Canine parvovirus 2 |
| >ON646218.1 | Lanzhou, Gansu | Feline parvovirus |
| >ON646217.1 | Lanzhou, Gansu | Feline parvovirus |
| >ON646216.1 | Lanzhou, Gansu | Feline parvovirus |
| >ON646215.1 | Lanzhou, Gansu | Feline parvovirus |
| >ON646214.1 | Lanzhou, Gansu | Feline parvovirus |
| >ON646213.1 | Lanzhou, Gansu | Feline parvovirus |
| >ON646212.1 | Lanzhou, Gansu | Feline parvovirus |
| >ON646211.1 | Lanzhou, Gansu | Feline parvovirus |
| >ON646210.1 | Lanzhou, Gansu | Feline parvovirus |
| >ON646209.1 | Lanzhou, Gansu | Feline parvovirus |
| >ON646208.1 | Lanzhou, Gansu | Feline parvovirus |
| >ON646207.1 | Lanzhou, Gansu | Feline parvovirus |
| >ON646206.1 | Lanzhou, Gansu | Feline parvovirus |
| >ON646205.1 | Lanzhou, Gansu | Feline parvovirus |
| >ON646204.1 | Lanzhou, Gansu | Feline parvovirus |
| >ON646203.1 | Lanzhou, Gansu | Feline parvovirus |
| >ON646202.1 | Lanzhou, Gansu | Feline parvovirus |
| >ON646201.1 | Lanzhou, Gansu | Feline parvovirus |
| >MZ056892.1 | Egypt | Canine parvovirus 2 |
| >MZ056891.1 | Egypt | Canine parvovirus 2 |
| >MZ056890.1 | Egypt | Canine parvovirus 2 |
| >MZ056889.1 | Egypt | Canine parvovirus 2 |
| >MZ056888.1 | Egypt | Canine parvovirus 2 |
| >MZ056887.1 | Egypt | Canine parvovirus 2 |
| >MZ056886.1 | Egypt | Canine parvovirus 2 |
| >MZ056885.1 | Egypt | Canine parvovirus 2 |
| >MZ056884.1 | Egypt | Canine parvovirus 2 |
| >MZ056883.1 | Egypt | Canine parvovirus 2 |
| >MZ056882.1 | Egypt | Canine parvovirus 2 |
| >MZ056881.1 | Egypt | Canine parvovirus 2 |
| >MN270961.1 | Thailand | Canine parvovirus |
| >MN270960.1 | Thailand | Canine parvovirus |
| >MN270959.1 | Thailand | Canine parvovirus |
| >MN270958.1 | Thailand | Canine parvovirus |
| >MN270957.1 | Thailand | Canine parvovirus |
| >MN270956.1 | Thailand | Canine parvovirus |
| >MN270955.1 | Thailand | Canine parvovirus |
| >MN270954.1 | Thailand | Canine parvovirus |
| >MN270953.1 | Thailand | Canine parvovirus |
| >MN270952.1 | Thailand | Canine parvovirus |
| >MN270951.1 | Thailand | Canine parvovirus |
| >MN270950.1 | Thailand | Canine parvovirus |
| >MN270949.1 | Thailand | Canine parvovirus |
| >MN270948.1 | Thailand | Canine parvovirus |
| >MN270947.1 | Thailand | Canine parvovirus |
| >MN270946.1 | Thailand | Canine parvovirus |
| >MN270945.1 | Thailand | Canine parvovirus |
| >MN270944.1 | Thailand | Canine parvovirus |
| >MN270943.1 | Thailand | Canine parvovirus |
| >MN270942.1 | Thailand | Canine parvovirus |
| >MN270941.1 | Thailand | Canine parvovirus |
| >MN270940.1 | Thailand | Canine parvovirus |
| >MN270939.1 | Thailand | Canine parvovirus |
| >MN270938.1 | Thailand | Canine parvovirus |
| >MK867453.1 | UK | Canine parvovirus |
| >MK867452.1 | UK | Canine parvovirus |
| >MK867451.1 | UK | Canine parvovirus |
| >MK867450.1 | UK | Canine parvovirus |
| >MK867449.1 | UK | Canine parvovirus |
| >MK867448.1 | UK | Canine parvovirus |
| >MK867447.1 | UK | Canine parvovirus |
| >MK867446.1 | UK | Canine parvovirus |
| >MK867445.1 | UK | Canine parvovirus |
| >MK867444.1 | UK | Canine parvovirus |
| >MK867443.1 | UK | Canine parvovirus |
| >MK867442.1 | UK | Canine parvovirus |
| >MK867441.1 | UK | Canine parvovirus |
| >MK867440.1 | UK | Canine parvovirus |
| >MK473853.1 | India | Canine parvovirus |
| >MH643886.1 | South Korea | Canine parvovirus 2 |
| >MF541142.1 | Changchun, Jilin | Canine parvovirus |
| >MF541141.1 | Changchun, Jilin | Canine parvovirus |
| >MF467242.1 | Luoyang, Henan | Canine parvovirus |
| >MF467241.1 | Luoyang, Henan | Canine parvovirus |
| >MF467240.1 | Luoyang, Henan | Canine parvovirus |
| >MF467239.1 | Luoyang, Henan | Canine parvovirus |
| >MF467238.1 | Luoyang, Henan | Canine parvovirus |
| >MF467237.1 | Luoyang, Henan | Canine parvovirus |
| >MF467236.1 | Luoyang, Henan | Canine parvovirus |
| >MF467235.1 | Luoyang, Henan | Canine parvovirus |
| >MF467234.1 | Luoyang, Henan | Canine parvovirus |
| >MF467233.1 | Luoyang, Henan | Canine parvovirus |
| >MF467232.1 | Luoyang, Henan | Canine parvovirus |
| >MF467231.1 | Luoyang, Henan | Canine parvovirus |
| >MF467230.1 | Luoyang, Henan | Canine parvovirus |
| >MF467229.1 | Luoyang, Henan | Canine parvovirus |
| >MF467228.1 | Luoyang, Henan | Canine parvovirus |
| >MF467227.1 | Luoyang, Henan | Canine parvovirus |
| >MF467226.1 | Luoyang, Henan | Canine parvovirus |
| >MF467225.1 | Luoyang, Henan | Canine parvovirus |
| >MF467224.1 | Luoyang, Henan | Canine parvovirus |
| >KU662351.1 | Portugal | Canine parvovirus |
| >KU662350.1 | Portugal | Canine parvovirus |
| >KU662349.1 | Portugal | Canine parvovirus |
| >KP859578.1 | Croatia | Canine parvovirus 2c |
| >KP859577.1 | Croatia | Canine parvovirus 2c |
| >KP859576.1 | Croatia | Canine parvovirus 2c |
| >KP859575.1 | Croatia | Canine parvovirus 2c |
| >KP859574.1 | Croatia | Canine parvovirus 2c |
| >KR559896.1 | Portugal | Canine parvovirus 2a |
| >KR559895.1 | Portugal | Canine parvovirus 2b |
| >KR559894.1 | Portugal | Canine parvovirus 2c |
| >KR559893.1 | Portugal | Canine parvovirus 2c |
| >KR559892.1 | Portugal | Canine parvovirus 2b |
| >KR559891.1 | Portugal | Canine parvovirus 2a |
| >KR058183.1 | Changchun, Jilin | Canine parvovirus |
| >KR869678.1 | Beijing | Canine parvovirus 2a |
| >KR869677.1 | Beijing | Canine parvovirus 2a |
| >KR869676.1 | Beijing | Canine parvovirus 2a |
| >KR869675.1 | Beijing | Canine parvovirus 2a |
| >KR869674.1 | Beijing | Canine parvovirus 2a |
| >KR869673.1 | Beijing | Canine parvovirus 2a |
| >KR869672.1 | Beijing | Canine parvovirus 2a |
| >KR869671.1 | Beijing | Canine parvovirus 2a |
| >KR869670.1 | Beijing | Canine parvovirus 2a |
| >KR869669.1 | Beijing | Canine parvovirus 2a |
| >KR869668.1 | Beijing | Canine parvovirus 2a |
| >KR869667.1 | Beijing | Canine parvovirus 2a |
| >KR869666.1 | Beijing | Canine parvovirus 2a |
| >KR869665.1 | Beijing | Canine parvovirus 2a |
| >KR869664.1 | Beijing | Canine parvovirus 2a |
| >KR869663.1 | Beijing | Canine parvovirus 2a |
| >KR869662.1 | Beijing | Canine parvovirus 2a |
| >KR869661.1 | Beijing | Canine parvovirus 2a |
| >KR869660.1 | Beijing | Canine parvovirus 2a |
| >KR869659.1 | Beijing | Canine parvovirus 2a |
| >KR869658.1 | Beijing | Canine parvovirus 2a |
| >KR869657.1 | Beijing | Canine parvovirus 2a |
| >KR869656.1 | Beijing | Canine parvovirus 2a |
| >KR869655.1 | Beijing | Canine parvovirus 2a |
| >KR869654.1 | Beijing | Canine parvovirus 2a |
| >KR869653.1 | Beijing | Canine parvovirus 2a |
| >KR869652.1 | Beijing | Canine parvovirus 2a |
| >KR611522.1 | Changchun, Jilin | Canine parvovirus |
| >KR611521.1 | Changchun, Jilin | Canine parvovirus |
| >KR611520.1 | Changchun, Jilin | Canine parvovirus |
| >KR611519.1 | Changchun, Jilin | Canine parvovirus |
| >KR611518.1 | Changchun, Jilin | Canine parvovirus |
| >KR611517.1 | Changchun, Jilin | Canine parvovirus |
| >KR611516.1 | Changchun, Jilin | Canine parvovirus |
| >KR611515.1 | Changchun, Jilin | Canine parvovirus |
| >KR611514.1 | Changchun, Jilin | Canine parvovirus |
| >KR611513.1 | Changchun, Jilin | Canine parvovirus |
| >KR611512.1 | Changchun, Jilin | Canine parvovirus |
| >KR611511.1 | Changchun, Jilin | Canine parvovirus |
| >KR611510.1 | Changchun, Jilin | Canine parvovirus |
| >KR611509.1 | Changchun, Jilin | Canine parvovirus |
| >KR611508.1 | Changchun, Jilin | Canine parvovirus |
| >KR611507.1 | Changchun, Jilin | Canine parvovirus |
| >KR611506.1 | Changchun, Jilin | Canine parvovirus |
| >KR611505.1 | Changchun, Jilin | Canine parvovirus |
| >KR611504.1 | Changchun, Jilin | Canine parvovirus |
| >KR611503.1 | Changchun, Jilin | Canine parvovirus |
| >KR611502.1 | Changchun, Jilin | Canine parvovirus |
| >KR611501.1 | Changchun, Jilin | Canine parvovirus |
| >KR611500.1 | Changchun, Jilin | Canine parvovirus |
| >KR611499.1 | Changchun, Jilin | Canine parvovirus |
| >KR611498.1 | Changchun, Jilin | Canine parvovirus |
| >KR611497.1 | Changchun, Jilin | Canine parvovirus |
| >KR611496.1 | Changchun, Jilin | Canine parvovirus |
| >KR611495.1 | Changchun, Jilin | Canine parvovirus |
| >KR611494.1 | Changchun, Jilin | Canine parvovirus |
| >KR611493.1 | Changchun, Jilin | Canine parvovirus |
| >KR611492.1 | Changchun, Jilin | Canine parvovirus |
| >KR611491.1 | Changchun, Jilin | Canine parvovirus |
| >KR611490.1 | Changchun, Jilin | Canine parvovirus |
| >KR611489.1 | Changchun, Jilin | Canine parvovirus |
| >KR611488.1 | Changchun, Jilin | Canine parvovirus |
| >KR611487.1 | Changchun, Jilin | Canine parvovirus |
| >KR611486.1 | Changchun, Jilin | Canine parvovirus |
| >KR611485.1 | Changchun, Jilin | Canine parvovirus |
| >KR611484.1 | Changchun, Jilin | Canine parvovirus |
| >KR611483.1 | Changchun, Jilin | Canine parvovirus |
| >KR611482.1 | Changchun, Jilin | Canine parvovirus |
| >KR611481.1 | Changchun, Jilin | Canine parvovirus |
| >KR611480.1 | Changchun, Jilin | Canine parvovirus |
| >KR611479.1 | Changchun, Jilin | Canine parvovirus |
| >KR611478.1 | Changchun, Jilin | Canine parvovirus |
| >KR611477.1 | Changchun, Jilin | Canine parvovirus |
| >KR611476.1 | Changchun, Jilin | Canine parvovirus |
| >KR611475.1 | Changchun, Jilin | Canine parvovirus |
| >KR611474.1 | Changchun, Jilin | Canine parvovirus |
| >KR611473.1 | Changchun, Jilin | Canine parvovirus |
| >KR611472.1 | Changchun, Jilin | Canine parvovirus |
| >KR611471.1 | Changchun, Jilin | Canine parvovirus |
| >KR611470.1 | Changchun, Jilin | Canine parvovirus |
| >KR611469.1 | Changchun, Jilin | Canine parvovirus |
| >KR611468.1 | Changchun, Jilin | Canine parvovirus |
| >KR611467.1 | Changchun, Jilin | Canine parvovirus |
| >KR611466.1 | Changchun, Jilin | Canine parvovirus |
| >KR611465.1 | Changchun, Jilin | Canine parvovirus |
| >KR611464.1 | Changchun, Jilin | Canine parvovirus |
| >KR611463.1 | Changchun, Jilin | Canine parvovirus |
| >KR611462.1 | Changchun, Jilin | Canine parvovirus |
| >KR611461.1 | Changchun, Jilin | Canine parvovirus |
| >KR611460.1 | Changchun, Jilin | Canine parvovirus |
| >KR611459.1 | Changchun, Jilin | Canine parvovirus |
| >KP715717.1 | Thailand | Canine parvovirus |
| >KP715716.1 | Thailand | Canine parvovirus |
| >KP715715.1 | Thailand | Canine parvovirus |
| >KP715714.1 | Thailand | Canine parvovirus |
| >KP715713.1 | Thailand | Canine parvovirus |
| >KP715712.1 | Thailand | Canine parvovirus |
| >KP715711.1 | Thailand | Canine parvovirus |
| >KP715710.1 | Thailand | Canine parvovirus |
| >KP715709.1 | Thailand | Canine parvovirus |
| >KP715708.1 | Thailand | Canine parvovirus |
| >KP715707.1 | Thailand | Canine parvovirus |
| >KP715706.1 | Thailand | Canine parvovirus |
| >KP715705.1 | Thailand | Canine parvovirus |
| >KP715704.1 | Thailand | Canine parvovirus |
| >KP715703.1 | Thailand | Canine parvovirus |
| >KP715702.1 | Thailand | Canine parvovirus |
| >KP715701.1 | Thailand | Canine parvovirus |
| >KP715700.1 | Thailand | Canine parvovirus |
| >KP715699.1 | Thailand | Canine parvovirus |
| >KP715698.1 | Thailand | Canine parvovirus |
| >KP715697.1 | Thailand | Canine parvovirus |
| >KP715696.1 | Thailand | Canine parvovirus |
| >KP715695.1 | Thailand | Canine parvovirus |
| >KP715694.1 | Thailand | Canine parvovirus |
| >KP715693.1 | Thailand | Canine parvovirus |
| >KP715692.1 | Thailand | Canine parvovirus |
| >KP715691.1 | Thailand | Canine parvovirus |
| >KP715690.1 | Thailand | Canine parvovirus |
| >KP715689.1 | Thailand | Canine parvovirus |
| >KP715688.1 | Thailand | Canine parvovirus |
| >KP715687.1 | Thailand | Canine parvovirus |
| >KP715686.1 | Thailand | Canine parvovirus |
| >KP715685.1 | Thailand | Canine parvovirus |
| >KP715684.1 | Thailand | Canine parvovirus |
| >KP715683.1 | Thailand | Canine parvovirus |
| >KP715682.1 | Thailand | Canine parvovirus |
| >KP715681.1 | Thailand | Canine parvovirus |
| >KP715680.1 | Thailand | Canine parvovirus |
| >KP715679.1 | Thailand | Canine parvovirus |
| >KP715678.1 | Thailand | Canine parvovirus |
| >KP715677.1 | Thailand | Canine parvovirus |
| >KP715676.1 | Thailand | Canine parvovirus |
| >KP715675.1 | Thailand | Canine parvovirus |
| >KP715674.1 | Thailand | Canine parvovirus |
| >KP715673.1 | Thailand | Canine parvovirus |
| >KP715672.1 | Thailand | Canine parvovirus |
| >KP715671.1 | Thailand | Canine parvovirus |
| >KP715670.1 | Thailand | Canine parvovirus |
| >KP715669.1 | Thailand | Canine parvovirus |
| >KP715668.1 | Thailand | Canine parvovirus |
| >KP715667.1 | Thailand | Canine parvovirus |
| >KP715666.1 | Thailand | Canine parvovirus |
| >KP715665.1 | Thailand | Canine parvovirus |
| >KP715664.1 | Thailand | Canine parvovirus |
| >KP715663.1 | Thailand | Canine parvovirus |
| >KP715662.1 | Thailand | Canine parvovirus |
| >KP715661.1 | Thailand | Canine parvovirus |
| >KP715660.1 | Thailand | Canine parvovirus |
| >KP715659.1 | Thailand | Canine parvovirus |
| >KP715658.1 | Thailand | Canine parvovirus |
| >KM236569.1 | Argentina | Canine parvovirus |
| >KM236568.1 | Argentina | Canine parvovirus |
| >KF539805.1 | Hungary | Canine parvovirus |
| >KF539804.1 | Hungary | Canine parvovirus |
| >KF539803.1 | Hungary | Canine parvovirus |
| >KF539802.1 | Hungary | Canine parvovirus |
| >KF539801.1 | Hungary | Canine parvovirus |
| >KF539800.1 | Hungary | Canine parvovirus |
| >KF539799.1 | Hungary | Canine parvovirus |
| >KF539798.1 | Hungary | Canine parvovirus |
| >KF539797.1 | Hungary | Canine parvovirus |
| >KF539796.1 | Hungary | Canine parvovirus |
| >KF539795.1 | Hungary | Canine parvovirus |
| >KF539794.1 | Hungary | Canine parvovirus |
| >KF539793.1 | Hungary | Canine parvovirus |
| >KF539792.1 | Hungary | Canine parvovirus |
| >KF539791.1 | Hungary | Canine parvovirus |
| >KF539790.1 | Hungary | Canine parvovirus |
| >KF539789.1 | Hungary | Canine parvovirus |
| >KJ194463.1 | Jilin, China | Canine parvovirus |
| >KJ194462.1 | Jilin, China | Canine parvovirus |
| >KF785798.1 | Guangdong, China | Canine parvovirus |
| >KF785797.1 | Guangdong, China | Canine parvovirus |
| >KF785796.1 | Guangdong, China | Canine parvovirus |
| >KF785795.1 | Guangdong, China | Canine parvovirus |
| >KF785794.1 | Guangdong, China | Canine parvovirus |
| >KF785793.1 | Guangdong, China | Canine parvovirus |
| >KF785792.1 | Guangdong, China | Canine parvovirus |
| >KF785791.1 | Guangdong, China | Canine parvovirus |
| >KF785790.1 | Guangdong, China | Canine parvovirus |
| >KF785789.1 | Guangdong, China | Canine parvovirus |
| >KF785788.1 | Guangdong, China | Canine parvovirus |
| >KC196114.1 | Uruguay | Canine parvovirus |
| >KC196113.1 | Uruguay | Canine parvovirus |
| >KC196112.1 | Uruguay | Canine parvovirus |
| >KC196111.1 | Uruguay | Canine parvovirus |
| >KC196110.1 | Uruguay | Canine parvovirus |
| >KC196109.1 | Uruguay | Canine parvovirus |
| >KC196108.1 | Uruguay | Canine parvovirus |
| >KC196107.1 | Uruguay | Canine parvovirus |
| >KC196106.1 | Uruguay | Canine parvovirus |
| >KC196105.1 | Uruguay | Canine parvovirus |
| >KC196104.1 | Uruguay | Canine parvovirus |
| >KC196103.1 | Uruguay | Canine parvovirus |
| >KC196102.1 | Uruguay | Canine parvovirus |
| >KC196101.1 | Uruguay | Canine parvovirus |
| >KC196100.1 | Uruguay | Canine parvovirus |
| >KC196099.1 | Uruguay | Canine parvovirus |
| >KC196098.1 | Uruguay | Canine parvovirus |
| >KC196097.1 | Uruguay | Canine parvovirus |
| >KC196096.1 | Uruguay | Canine parvovirus |
| >KC196095.1 | Uruguay | Canine parvovirus |
| >KC196094.1 | Uruguay | Canine parvovirus |
| >KC196093.1 | Uruguay | Canine parvovirus |
| >KC196092.1 | Uruguay | Canine parvovirus |
| >KC196091.1 | Uruguay | Canine parvovirus |
| >KC196090.1 | Uruguay | Canine parvovirus |
| >KC196089.1 | Uruguay | Canine parvovirus |
| >KC196088.1 | Uruguay | Canine parvovirus |
| >KC196087.1 | Uruguay | Canine parvovirus |
| >KC196086.1 | Uruguay | Canine parvovirus |
| >KC196085.1 | Uruguay | Canine parvovirus |
| >KC196084.1 | Uruguay | Canine parvovirus |
| >KC196083.1 | Uruguay | Canine parvovirus |
| >KC196082.1 | Uruguay | Canine parvovirus |
| >KC196081.1 | Uruguay | Canine parvovirus |
| >KC196080.1 | Uruguay | Canine parvovirus |
| >KC196079.1 | Uruguay | Canine parvovirus |
| >JF767492.1 | Guangdong, China | Canine parvovirus |
| >HQ883273.1 | Jilin, China | Canine parvovirus |
| >HQ883271.1 | Jilin, China | Canine parvovirus |
| >HQ883269.1 | Jilin, China | Canine parvovirus |
| >HQ883267.1 | Jilin, China | Canine parvovirus |
| >FJ435348.1 | Jilin, China | Canine parvovirus |
| >FJ435347.1 | Jilin, China | Canine parvovirus |
| >FJ435346.1 | Jilin, China | Canine parvovirus |
| >FJ435345.1 | Jilin, China | Canine parvovirus |
| >FJ435344.1 | Jilin, China | Canine parvovirus |
| >FJ435343.1 | Jilin, China | Canine parvovirus |
| >FJ435342.1 | Jilin, China | Canine parvovirus |
| >ON063564.1 | Nigerian | Canine parvovirus |
| >ON063563.1 | Nigerian | Canine parvovirus |
| >ON063562.1 | Nigerian | Canine parvovirus |
| >ON063561.1 | Nigerian | Canine parvovirus |
| >ON063560.1 | Nigerian | Canine parvovirus |
| >ON063559.1 | Nigerian | Canine parvovirus |
| >ON063558.1 | Nigerian | Canine parvovirus |
| >ON063557.1 | Nigerian | Canine parvovirus |
| >ON063556.1 | Nigerian | Canine parvovirus |
| >ON063555.1 | Nigerian | Canine parvovirus |
| >ON063554.1 | Nigerian | Canine parvovirus |
| >ON063553.1 | Nigerian | Canine parvovirus |
| >ON063552.1 | Nigerian | Canine parvovirus |
| >ON063551.1 | Nigerian | Canine parvovirus |
| >ON063550.1 | Nigerian | Canine parvovirus |
| >ON063549.1 | Nigerian | Canine parvovirus |
| >ON063550.1 | Nigerian | Canine parvovirus |
| >ON063547.1 | Nigerian | Canine parvovirus |
| >ON063546.1 | Nigerian | Canine parvovirus |
| >ON063545.1 | Nigerian | Canine parvovirus |
| >ON063544.1 | Nigerian | Canine parvovirus |
| >ON063543.1 | Nigerian | Canine parvovirus |
| >MZ576582.1 | Beijing, China | Canine parvovirus |
| >MZ576581.1 | Beijing, China | Canine parvovirus |
| >MZ576580.1 | Beijing, China | Canine parvovirus |
| >MZ576579.1 | Beijing, China | Canine parvovirus |
| >MZ576578.1 | Beijing, China | Canine parvovirus |
| >MZ576577.1 | Beijing, China | Canine parvovirus |
| >MZ576576.1 | Beijing, China | Canine parvovirus |
| >MZ576575.1 | Beijing, China | Canine parvovirus |
| >OM937915.1 | Egypt | Canine parvovirus |
| >OM937914.1 | Egypt | Canine parvovirus |
| >OM937913.1 | Egypt | Canine parvovirus |
| >OM937912.1 | Egypt | Canine parvovirus |
| >OM937911.1 | Egypt | Canine parvovirus |
| >OM937910.1 | Egypt | Canine parvovirus |
| >OM937909.1 | Egypt | Canine parvovirus |
| >OM937908.1 | Egypt | Canine parvovirus |
| >OM937907.1 | Egypt | Canine parvovirus |
| >OM057679.1 | Jilin, China | Canine parvovirus |
| >OM057681.1 | Jilin, China | Canine parvovirus |
| >OM057680.1 | Jilin, China | Canine parvovirus |
| >OM057678.1 | Jilin, China | Canine parvovirus |
| >OM057677.1 | Jilin, China | Canine parvovirus |
| >OM057676.1 | Jilin, China | Canine parvovirus |
| >OM057675.1 | Jilin, China | Canine parvovirus |
| >OM057674.1 | Jilin, China | Canine parvovirus |
| >OM057673.1 | Jilin, China | Canine parvovirus |
| >MW791425.1 | Jiangsu , China | Canine parvovirus |
| >MW048581.1 | Jiangsu , China | Canine parvovirus |
| >MW048580.1 | Jiangsu , China | Canine parvovirus |
| >MW048579.1 | Jiangsu , China | Canine parvovirus |
| >MW048578.1 | Jiangsu , China | Canine parvovirus |
| >MW048577.1 | Jiangsu , China | Canine parvovirus |
| >MW048576.1 | Jiangsu , China | Canine parvovirus |
| >MW048575.1 | Jiangsu , China | Canine parvovirus |
| >MW048574.1 | Jiangsu , China | Canine parvovirus |
| >MW048573.1 | Jiangsu , China | Canine parvovirus |
| >MW048572.1 | Jiangsu , China | Canine parvovirus |
| >MW048571.1 | Jiangsu , China | Canine parvovirus |
| >MW048570.1 | Jiangsu , China | Canine parvovirus |
| >MW048569.1 | Jiangsu , China | Canine parvovirus |
| >MW048568.1 | Jiangsu , China | Canine parvovirus |
| >MW048567.1 | Jiangsu , China | Canine parvovirus |
| >MW048566.1 | Jiangsu , China | Canine parvovirus |
| >MW048565.1 | Jiangsu , China | Canine parvovirus |
| >MW048564.1 | Jiangsu , China | Canine parvovirus |
| >MW048563.1 | Jiangsu , China | Canine parvovirus |
| >MW048562.1 | Jiangsu , China | Canine parvovirus |
| >MW048561.1 | Jiangsu , China | Canine parvovirus |
| >MW048560.1 | Jiangsu , China | Canine parvovirus |
| >MW017624.1 | Jiangsu , China | Canine parvovirus |
| >MW017623.1 | Jiangsu , China | Canine parvovirus |
| >MW017622.1 | Jiangsu , China | Canine parvovirus |
| >MW017621.1 | Jiangsu , China | Canine parvovirus |
| >MW017620.1 | Jiangsu , China | Canine parvovirus |
| >MW017619.1 | Jiangsu , China | Canine parvovirus |
| >MW017617.1 | Jiangsu , China | Canine parvovirus |
| >MW017615.1 | Jiangsu , China | Canine parvovirus |
| >MW017614.1 | Jiangsu , China | Canine parvovirus |
| >MW017613.1 | Jiangsu , China | Canine parvovirus |
| >MW017612.1 | Jiangsu , China | Canine parvovirus |
| >MW017611.1 | Jiangsu , China | Canine parvovirus |
| >MW017610.1 | Jiangsu , China | Canine parvovirus |
| >MW017609.1 | Jiangsu , China | Canine parvovirus |
| >MW017608.1 | Jiangsu , China | Canine parvovirus |
| >MW017607.1 | Jiangsu , China | Canine parvovirus |
| >MW017606.1 | Jiangsu , China | Canine parvovirus |
| >MW017605.1 | Jiangsu , China | Canine parvovirus |
| >MW017604.1 | Jiangsu , China | Canine parvovirus |
| >MW017603.1 | Jiangsu , China | Canine parvovirus |
| >MW017602.1 | Jiangsu , China | Canine parvovirus |
| >MW017601.1 | Jiangsu , China | Canine parvovirus |
| >MW017600.1 | Jiangsu , China | Canine parvovirus |
| >MW017599.1 | Jiangsu , China | Canine parvovirus |
| >MW017598.1 | Jiangsu , China | Canine parvovirus |
| >MW017597.1 | Jiangsu , China | Canine parvovirus |
| >MW017595.1 | Jiangsu , China | Canine parvovirus |
| >MW017594.1 | Jiangsu , China | Canine parvovirus |
| >MW017593.1 | Jiangsu , China | Canine parvovirus |
| >MW017592.1 | Jiangsu , China | Canine parvovirus |
| >MW017591.1 | Jiangsu , China | Canine parvovirus |
| >MW017590.1 | Jiangsu , China | Canine parvovirus |
| >MW017589.1 | Jiangsu , China | Canine parvovirus |
| >MW017588.1 | Jiangsu , China | Canine parvovirus |
| >MW017587.1 | Jiangsu , China | Canine parvovirus |
| >MW017586.1 | Jiangsu , China | Canine parvovirus |
| >MW017585.1 | Jiangsu , China | Canine parvovirus |
| >MW017584.1 | Jiangsu , China | Canine parvovirus |
| >MW017583.1 | Jiangsu , China | Canine parvovirus |
| >MW017582.1 | Jiangsu , China | Canine parvovirus |
| >MW017581.1 | Jiangsu , China | Canine parvovirus |
| >MW017580.1 | Jiangsu , China | Canine parvovirus |
| >MW017579.1 | Jiangsu , China | Canine parvovirus |
| >MW017578.1 | Jiangsu , China | Canine parvovirus |
| >MW017577.1 | Jiangsu , China | Canine parvovirus |
| >MW017576.1 | Jiangsu , China | Canine parvovirus |
| >MW017575.1 | Jiangsu , China | Canine parvovirus |
| >MW017574.1 | Jiangsu , China | Canine parvovirus |
| >MW017573.1 | Jiangsu , China | Canine parvovirus |
| >MT353764.1 | Italy | Canine parvovirus |
| >MT353763.1 | Italy | Canine parvovirus |
| >MT353762.1 | Italy | Canine parvovirus |
| >MT353761.1 | Italy | Canine parvovirus |
| >MT353760.1 | Italy | Canine parvovirus |
| >MK266800.1 | Jilin, China | Canine parvovirus |
| >MK266799.1 | Jilin, China | Canine parvovirus |
| >MK266798.1 | Jilin, China | Canine parvovirus |
| >MK266797.1 | Jilin, China | Canine parvovirus |
| >MK266795.1 | Jilin, China | Canine parvovirus |
| >MK266794.1 | Jilin, China | Canine parvovirus |
| >MK266793.1 | Jilin, China | Canine parvovirus |
| >MK266792.1 | Jilin, China | Canine parvovirus |
| >MK266791.1 | Jilin, China | Canine parvovirus |
| >MK266790.1 | Jilin, China | Canine parvovirus |
| >MK266789.1 | Jilin, China | Canine parvovirus |
| >MK266788.1 | Jilin, China | Canine parvovirus |
| >MK266787.1 | Jilin, China | Canine parvovirus |
| >MK266786.1 | Jilin, China | Canine parvovirus |
| >MK266785.1 | Jilin, China | Canine parvovirus |
| >MK266784.1 | Jilin, China | Canine parvovirus |
| >MK266783.1 | Jilin, China | Canine parvovirus |
| >MK266782.1 | Jilin, China | Canine parvovirus |
| >MK675667.1 | Jilin, China | Canine parvovirus |
| >MK675666.1 | Jilin, China | Canine parvovirus |
| >MK675665.1 | Jilin, China | Canine parvovirus |
| >MK675664.1 | Jilin, China | Canine parvovirus |
| >MK675663.1 | Jilin, China | Canine parvovirus |
| >MK675662.1 | Jilin, China | Canine parvovirus |
| >MK675661.1 | Jilin, China | Canine parvovirus |
| >MK675660.1 | Jilin, China | Canine parvovirus |
| >MK675659.1 | Jilin, China | Canine parvovirus |
| >MK675658.1 | Jilin, China | Canine parvovirus |
| >MK675657.1 | Jilin, China | Canine parvovirus |
| >MH329288.1 | Henan, China | Canine parvovirus |
| >MH329287.1 | Henan, China | Canine parvovirus |
| >MH329286.1 | Henan, China | Canine parvovirus |
| >MH329285.1 | Henan, China | Canine parvovirus |
| >MH329284.1 | Henan, China | Canine parvovirus |
| >MH329283.1 | Henan, China | Canine parvovirus |
| >MH213141.1 | Korea | Canine parvovirus |
| >MH213140.1 | Korea | Canine parvovirus |
| >MH213139.1 | Korea | Canine parvovirus |
| >MH213138.1 | Korea | Canine parvovirus |
| >MH213137.1 | Korea | Canine parvovirus |
| >MH213136.1 | Korea | Canine parvovirus |
| >MH213135.1 | Korea | Canine parvovirus |
| >MH155193.1 | Gansu, China | Canine parvovirus |
| >MH155192.1 | Gansu, China | Canine parvovirus |
| >KP881675.1 | New Zealand | Canine parvovirus |
| >KP686093.1 | New Zealand | Canine parvovirus |
| >JX475288.1 | Ithaca, USA | Canine parvovirus |
| >JX475287.1 | Ithaca, USA | Canine parvovirus |
| >JX475286.1 | Ithaca, USA | Canine parvovirus |
| >JX475285.1 | Ithaca, USA | Canine parvovirus |
| >JX475284.1 | Ithaca, USA | Canine parvovirus |
| >JX475283.1 | Ithaca, USA | Canine parvovirus |
| >JX475282.1 | Ithaca, USA | Canine parvovirus |
| >JX475281.1 | Ithaca, USA | Canine parvovirus |
| >JX475280.1 | Ithaca, USA | Canine parvovirus |
| >JX475279.1 | Ithaca, USA | Canine parvovirus |
| >JX475278.1 | Ithaca, USA | Canine parvovirus |
| >JX475277.1 | Ithaca, USA | Canine parvovirus |
| >JX475276.1 | Ithaca, USA | Canine parvovirus |
| >JX475275.1 | Ithaca, USA | Canine parvovirus |
| >JX475274.1 | Ithaca, USA | Canine parvovirus |
| >JX475273.1 | Ithaca, USA | Canine parvovirus |
| >JX475272.1 | Ithaca, USA | Canine parvovirus |
| >JX475271.1 | Ithaca, USA | Canine parvovirus |
| >JX475269.1 | Ithaca, USA | Canine parvovirus |
| >JX475268.1 | Ithaca, USA | Canine parvovirus |
| >JX475267.1 | Ithaca, USA | Canine parvovirus |
| >JX475266.1 | Ithaca, USA | Canine parvovirus |
| >JX475265.1 | Ithaca, USA | Canine parvovirus |
| >JX475264.1 | Ithaca, USA | Canine parvovirus |
| >JX475263.1 | Ithaca, USA | Canine parvovirus |
| >JX475262.1 | Ithaca, USA | Canine parvovirus |
| >JX475261.1 | Ithaca, USA | Canine parvovirus |
| >JX475260.1 | Ithaca, USA | Canine parvovirus |
| >JX475258.1 | Ithaca, USA | Canine parvovirus |
| >JX475257.1 | Ithaca, USA | Canine parvovirus |
| >JX475252.1 | Ithaca, USA | Canine parvovirus |
| >JX475251.1 | Ithaca, USA | Canine parvovirus |
| >JX475250.1 | Ithaca, USA | Canine parvovirus |
| >JX475249.1 | Ithaca, USA | Canine parvovirus |
| >JX475248.1 | Ithaca, USA | Canine parvovirus |
| >JX475247.1 | Ithaca, USA | Canine parvovirus |
| >JX475246.1 | Ithaca, USA | Canine parvovirus |
| >JX475244.1 | Ithaca, USA | Canine parvovirus |
| >JX475243.1 | Ithaca, USA | Canine parvovirus |
| >JX475242.1 | Ithaca, USA | Canine parvovirus |
| >JX475241.1 | Ithaca, USA | Canine parvovirus |
| >JX475240.1 | Ithaca, USA | Canine parvovirus |
| >JX475239.1 | Ithaca, USA | Canine parvovirus |
| >JX475238.1 | Ithaca, USA | Canine parvovirus |
| >JX475237.1 | Ithaca, USA | Canine parvovirus |
| >JX475236.1 | Ithaca, USA | Canine parvovirus |
| >JX475235.1 | Ithaca, USA | Canine parvovirus |
| >JX475234.1 | Ithaca, USA | Canine parvovirus |
| >JX475233.1 | Ithaca, USA | Canine parvovirus |
| >JX475232.1 | Ithaca, USA | Canine parvovirus |
| >JX475231.1 | Ithaca, USA | Canine parvovirus |
| >JF767494.1 | Guangdong, China | Canine parvovirus |
| >JF767493.1 | Guangdong, China | Canine parvovirus |
| >EU914139.1 | Taiwan, China | Canine parvovirus |
| >EF189717.1 | Kwanjin-gu, South Korea | Canine parvovirus |
| >MK266802.1 | Jilin, China | Feline panleukopenia virus |
| >MK266796.1 | Jilin, China | Feline panleukopenia virus |
| >MT270585.1 | Beijing, China | Feline panleukopenia virus |
| >MT270584.1 | Beijing, China | Feline panleukopenia virus |
| >MT270583.1 | Beijing, China | Feline panleukopenia virus |
| >MT270582.1 | Beijing, China | Feline panleukopenia virus |
| >MT270581.1 | Beijing, China | Feline panleukopenia virus |
| >MT270580.1 | Beijing, China | Feline panleukopenia virus |
| >MT270579.1 | Beijing, China | Feline panleukopenia virus |
| >MT270578.1 | Beijing, China | Feline panleukopenia virus |
| >MT270577.1 | Beijing, China | Feline panleukopenia virus |
| >MT270576.1 | Beijing, China | Feline panleukopenia virus |
| >MT270575.1 | Beijing, China | Feline panleukopenia virus |
| >MT270574.1 | Beijing, China | Feline panleukopenia virus |
| >MT270573.1 | Beijing, China | Feline panleukopenia virus |
| >MT270572.1 | Beijing, China | Feline panleukopenia virus |
| >MT270571.1 | Beijing, China | Feline panleukopenia virus |
| >MT270570.1 | Beijing, China | Feline panleukopenia virus |
| >MT270569.1 | Beijing, China | Feline panleukopenia virus |
| >MT270568.1 | Beijing, China | Feline panleukopenia virus |
| >MT270567.1 | Beijing, China | Feline panleukopenia virus |
| >MT270566.1 | Beijing, China | Feline panleukopenia virus |
| >MT270565.1 | Beijing, China | Feline panleukopenia virus |
| >MT270564.1 | Beijing, China | Feline panleukopenia virus |
| >MT270563.1 | Beijing, China | Feline panleukopenia virus |
| >MT270562.1 | Beijing, China | Feline panleukopenia virus |
| >MT270561.1 | Beijing, China | Feline panleukopenia virus |
| >MT270560.1 | Beijing, China | Feline panleukopenia virus |
| >MT270559.1 | Beijing, China | Feline panleukopenia virus |
| >MT270558.1 | Beijing, China | Feline panleukopenia virus |
| >MT270557.1 | Beijing, China | Feline panleukopenia virus |
| >MT270556.1 | Beijing, China | Feline panleukopenia virus |
| >MT270555.1 | Beijing, China | Feline panleukopenia virus |
| >MT270554.1 | Beijing, China | Feline panleukopenia virus |
| >MT270553.1 | Beijing, China | Feline panleukopenia virus |
| >MT270552.1 | Beijing, China | Feline panleukopenia virus |
| >MT270551.1 | Beijing, China | Feline panleukopenia virus |
| >MT270550.1 | Beijing, China | Feline panleukopenia virus |
| >MT270549.1 | Beijing, China | Feline panleukopenia virus |
| >MT270548.1 | Beijing, China | Feline panleukopenia virus |
| >MT270547.1 | Beijing, China | Feline panleukopenia virus |
| >MT270546.1 | Beijing, China | Feline panleukopenia virus |
| >MT270545.1 | Beijing, China | Feline panleukopenia virus |
| >MT270544.1 | Beijing, China | Feline panleukopenia virus |
| >MT270543.1 | Beijing, China | Feline panleukopenia virus |
| >MT270542.1 | Beijing, China | Feline panleukopenia virus |
| >MT270541.1 | Beijing, China | Feline panleukopenia virus |
| >MT270540.1 | Beijing, China | Feline panleukopenia virus |
| >MT270539.1 | Beijing, China | Feline panleukopenia virus |
| >MT270538.1 | Beijing, China | Feline panleukopenia virus |
| >MT270537.1 | Beijing, China | Feline panleukopenia virus |
| >MT270536.1 | Beijing, China | Feline panleukopenia virus |
| >MT270535.1 | Beijing, China | Feline panleukopenia virus |
| >MT270534.1 | Beijing, China | Feline panleukopenia virus |
| >MT270533.1 | Beijing, China | Feline panleukopenia virus |
| >MT270532.1 | Beijing, China | Feline panleukopenia virus |
| >MT270531.1 | Beijing, China | Feline panleukopenia virus |
| >MT029328.1 | Hebei, China | Canine parvovirus |
| >MT029327.1 | Hebei, China | Canine parvovirus |
| >MT029326.1 | Hebei, China | Canine parvovirus |
| >MT029325.1 | Hebei, China | Canine parvovirus |
| >MT029324.1 | Hebei, China | Canine parvovirus |
| >MT029323.1 | Hebei, China | Canine parvovirus |
| >MT029322.1 | Hebei, China | Canine parvovirus |
| >MT029321.1 | Hebei, China | Canine parvovirus |
| >MT029320.1 | Hebei, China | Canine parvovirus |
| >MT029319.1 | Hebei, China | Canine parvovirus |
| >KT162046.1 | Jilin, China | Canine parvovirus |
| >KT162045.1 | Jilin, China | Canine parvovirus |
| >KT162044.1 | Jilin, China | Canine parvovirus |
| >KT162043.1 | Jilin, China | Canine parvovirus |
| >KT162042.1 | Jilin, China | Canine parvovirus |
| >KT162041.1 | Jilin, China | Canine parvovirus |
| >KT162040.1 | Jilin, China | Canine parvovirus |
| >KT162039.1 | Jilin, China | Canine parvovirus |
| >KT162038.1 | Jilin, China | Canine parvovirus |
| >KT162037.1 | Jilin, China | Canine parvovirus |
| >KT162036.1 | Jilin, China | Canine parvovirus |
| >KT162035.1 | Jilin, China | Canine parvovirus |
| >KT162034.1 | Jilin, China | Canine parvovirus |
| >KT162033.1 | Jilin, China | Canine parvovirus |
| >KT162032.1 | Jilin, China | Canine parvovirus |
| >KT162031.1 | Jilin, China | Canine parvovirus |
| >KT162030.1 | Jilin, China | Canine parvovirus |
| >KT162029.1 | Jilin, China | Canine parvovirus |
| >KT162028.1 | Jilin, China | Canine parvovirus |
| >KT162027.1 | Jilin, China | Canine parvovirus |
| >KT162026.1 | Jilin, China | Canine parvovirus |
| >KT162025.1 | Jilin, China | Canine parvovirus |
| >KT162024.1 | Jilin, China | Canine parvovirus |
| >KT162023.1 | Jilin, China | Canine parvovirus |
| >KT162022.1 | Jilin, China | Canine parvovirus |
| >KT162021.1 | Jilin, China | Canine parvovirus |
| >KT162020.1 | Jilin, China | Canine parvovirus |
| >KT162019.1 | Jilin, China | Canine parvovirus |
| >KT162018.1 | Jilin, China | Canine parvovirus |
| >KT162017.1 | Jilin, China | Canine parvovirus |
| >KT162016.1 | Jilin, China | Canine parvovirus |
| >KT162015.1 | Jilin, China | Canine parvovirus |
| >KT162014.1 | Jilin, China | Canine parvovirus |
| >KT162013.1 | Jilin, China | Canine parvovirus |
| >KT162012.1 | Jilin, China | Canine parvovirus |
| >KT162011.1 | Jilin, China | Canine parvovirus |
| >KT162010.1 | Jilin, China | Canine parvovirus |
| >KT162009.1 | Jilin, China | Canine parvovirus |
| >KT162008.1 | Jilin, China | Canine parvovirus |
| >KT162007.1 | Jilin, China | Canine parvovirus |
| >KT162006.1 | Jilin, China | Canine parvovirus |
| >KT162005.1 | Jilin, China | Canine parvovirus |
| >FJ011098.1 | Taiwan, China | Canine parvovirus |
| >FJ011097.1 | Taiwan, China | Canine parvovirus |
| >OP208806.1 | southern China(title) | Canine parvovirus |
| >OP208805.1 | southern China(title) | Canine parvovirus |
| >MW570769.1 | Jilin, China | Canine parvovirus |
| >MZ836372.1 | northern China（title） | Canine parvovirus |
| >MZ836349.1 | northern China | Canine parvovirus |
| >MZ836348.1 | northern China | Canine parvovirus |
| >MZ836346.1 | northern China | Canine parvovirus |
| >MZ836345.1 | northern China | Canine parvovirus |
| >MZ836344.1 | northern China | Canine parvovirus |
| >MZ836343.1 | northern China | Canine parvovirus |
| >MZ836342.1 | northern China | Canine parvovirus |
| >MZ836341.1 | northern China | Canine parvovirus |
| >MZ836340.1 | northern China | Canine parvovirus |
| >MZ836339.1 | northern China | Canine parvovirus |
| >MZ836338.1 | northern China | Canine parvovirus |
| >MZ836337.1 | northern China | Canine parvovirus |
| >MZ836336.1 | northern China | Canine parvovirus |
| >MZ836335.1 | northern China | Canine parvovirus |
| >MZ836334.1 | northern China | Canine parvovirus |
| >MZ836333.1 | northern China | Canine parvovirus |
| >MZ836332.1 | northern China | Canine parvovirus |
| >MZ836331.1 | northern China | Canine parvovirus |
| >MZ836330.1 | northern China | Canine parvovirus |
| >MZ836329.1 | northern China | Canine parvovirus |
| >MZ836328.1 | northern China | Canine parvovirus |
| >MZ836327.1 | northern China | Canine parvovirus |
| >MZ836326.1 | northern China | Canine parvovirus |
| >MZ836325.1 | northern China | Canine parvovirus |
| >MZ836324.1 | northern China | Canine parvovirus |
| >MZ836323.1 | northern China | Canine parvovirus |
| >MZ836322.1 | northern China | Canine parvovirus |
| >MZ836321.1 | northern China | Canine parvovirus |
| >MZ836320.1 | northern China | Canine parvovirus |
| >MZ836319.1 | northern China | Canine parvovirus |
| >MZ836318.1 | northern China | Canine parvovirus |
| >MZ836317.1 | northern China | Canine parvovirus |
| >MZ836316.1 | northern China | Canine parvovirus |
| >MZ836315.1 | northern China | Canine parvovirus |
| >MZ836314.1 | northern China | Canine parvovirus |
| >MZ836313.1 | northern China | Canine parvovirus |
| >MZ836312.1 | northern China | Canine parvovirus |
| >MZ836311.1 | northern China | Canine parvovirus |
| >MZ836310.1 | northern China | Canine parvovirus |
| >MZ836309.1 | northern China | Canine parvovirus |
| >MZ836308.1 | northern China | Canine parvovirus |
| >MZ836307.1 | northern China | Canine parvovirus |
| >MZ836306.1 | northern China | Canine parvovirus |
| >MZ836305.1 | northern China | Canine parvovirus |
| >MZ836304.1 | northern China | Canine parvovirus |
| >MZ836303.1 | northern China | Canine parvovirus |
| >MZ836302.1 | northern China | Canine parvovirus |
| >MZ836301.1 | northern China | Canine parvovirus |
| >MZ836300.1 | northern China | Canine parvovirus |
| >MN270937.1 | Thailand | Feline panleukopenia virus |
| >MK982094.1 | Jilin, China | Feline parvovirus |
| >MF541140.1 | Jilin, China | Feline panleukopenia virus |
| >MF541139.1 | Jilin, China | Feline panleukopenia virus |
| >MF541138.1 | Jilin, China | Feline panleukopenia virus |
| >MF541137.1 | Jilin, China | Feline panleukopenia virus |
| >MF541136.1 | Jilin, China | Feline panleukopenia virus |
| >MF541135.1 | Jilin, China | Feline panleukopenia virus |
| >MF541134.1 | Jilin, China | Feline panleukopenia virus |
| >MF541133.1 | Jilin, China | Feline panleukopenia virus |
| >MF541132.1 | Jilin, China | Feline panleukopenia virus |
| >MF541131.1 | Jilin, China | Feline panleukopenia virus |
| >MF541130.1 | Jilin, China | Feline panleukopenia virus |
| >MF541129.1 | Jilin, China | Feline panleukopenia virus |
| >MF541128.1 | Jilin, China | Feline panleukopenia virus |
| >MF541127.1 | Jilin, China | Feline panleukopenia virus |
| >MF541126.1 | Jilin, China | Feline panleukopenia virus |
| >MF541125.1 | Jilin, China | Feline panleukopenia virus |
| >MF541124.1 | Jilin, China | Feline panleukopenia virus |
| >MF541123.1 | Jilin, China | Feline panleukopenia virus |
| >MF541122.1 | Jilin, China | Feline panleukopenia virus |
| >MF541121.1 | Jilin, China | Feline panleukopenia virus |
| >MF541120.1 | Jilin, China | Feline panleukopenia virus |
| >MF541119.1 | Jilin, China | Feline panleukopenia virus |
| >KY386859.1 | Guizhou, China | Canine parvovirus |
| >KY386858.1 | Guizhou, China | Canine parvovirus |
| >KY386857.1 | Guizhou, China | Canine parvovirus |
| >KY386856.1 | Guizhou, China | Canine parvovirus |
| >KY386855.1 | Guizhou, China | Canine parvovirus |
| >KY386854.1 | Guizhou, China | Canine parvovirus |
| >KY386853.1 | Guizhou, China | Canine parvovirus |
| >KY386852.1 | Guizhou, China | Canine parvovirus |
| >KY386851.1 | Guizhou, China | Canine parvovirus |
| >KY386850.1 | Guizhou, China | Canine parvovirus |
| >HQ883275.1 | Jilin, China | Mink enteritis virus |
| >FJ265784.1 | Taiwan, China | Canine parvovirus |
| >FJ265783.1 | Taiwan, China | Canine parvovirus |
| >FJ265782.1 | Taiwan, China | Canine parvovirus |
| >FJ265781.1 | Taiwan, China | Canine parvovirus |
| >FJ265780.1 | Taiwan, China | Canine parvovirus |
| >FJ265779.1 | Taiwan, China | Canine parvovirus |
| >FJ265778.1 | Taiwan, China | Canine parvovirus |
| >FJ265777.1 | Taiwan, China | Canine parvovirus |
| >FJ265776.1 | Taiwan, China | Canine parvovirus |
| >FJ265775.1 | Taiwan, China | Canine parvovirus |
| >FJ936171.1 | Jilin, China | Feline panleukopenia virus |
| >EU360959.1 | Asian(title) | Feline panleukopenia virus |
| >EU360958.1 | Asian(title) | Feline panleukopenia virus |
| >EU697387.1 | Asian(title) | Feline panleukopenia virus |
| >EU697386.1 | Asian(title) | Feline panleukopenia virus |
| >EU697384.1 | Asian(title) | Feline panleukopenia virus |
| >EU697383.1 | Asian(title) | Feline panleukopenia virus |
| >OM937916.1 | Egypt | Feline panleukopenia virus |
| >MT525955.1 | Jilin, China | Canine parvovirus |
| >MW791427.1 | Jiangsu, China | Feline panleukopenia virus |
| >MW791426.1 | Jiangsu, China | Feline panleukopenia virus |
| >MW017631.1 | Jiangsu, China | Feline panleukopenia virus |
| >MW017630.1 | Jiangsu, China | Feline panleukopenia virus |
| >MW017629.1 | Jiangsu, China | Feline panleukopenia virus |
| >MW017628.1 | Jiangsu, China | Feline panleukopenia virus |
| >MW017627.1 | Jiangsu, China | Feline panleukopenia virus |
| >MW017626.1 | Jiangsu, China | Feline panleukopenia virus |
| >MW017625.1 | Jiangsu, China | Feline panleukopenia virus |
| >MW017618.1 | Jiangsu, China | Feline panleukopenia virus |
| >MW017616.1 | Jiangsu, China | Feline panleukopenia virus |
| >MW017596.1 | Jiangsu, China | Feline panleukopenia virus |
| >MT274378.1 | Italy | Feline panleukopenia virus |
| >MT274377.1 | Italy | Feline panleukopenia virus |
| >MK671188.1 | Jilin, China | Feline panleukopenia virus |
| >MK671187.1 | Jilin, China | Feline panleukopenia virus |
| >MK671186.1 | Jilin, China | Feline panleukopenia virus |
| >MK671185.1 | Jilin, China | Feline panleukopenia virus |
| >MK671184.1 | Jilin, China | Feline panleukopenia virus |
| >MK671183.1 | Jilin, China | Feline panleukopenia virus |
| >MK671182.1 | Jilin, China | Feline panleukopenia virus |
| >MK671181.1 | Jilin, China | Feline panleukopenia virus |
| >MK671180.1 | Jilin, China | Feline panleukopenia virus |
| >MK671179.1 | Jilin, China | Feline panleukopenia virus |
| >MK671178.1 | Jilin, China | Feline panleukopenia virus |
| >MK671177.1 | Jilin, China | Feline panleukopenia virus |
| >MK671176.1 | Jilin, China | Feline panleukopenia virus |
| >MK671175.1 | Jilin, China | Feline panleukopenia virus |
| >MK671174.1 | Jilin, China | Feline panleukopenia virus |
| >MK671173.1 | Jilin, China | Feline panleukopenia virus |
| >MK671172.1 | Jilin, China | Feline panleukopenia virus |
| >MK671171.1 | Jilin, China | Feline panleukopenia virus |
| >MK671170.1 | Jilin, China | Feline panleukopenia virus |
| >MK671169.1 | Jilin, China | Feline panleukopenia virus |
| >MK671168.1 | Jilin, China | Feline panleukopenia virus |
| >MK671167.1 | Jilin, China | Feline panleukopenia virus |
| >MK671166.1 | Jilin, China | Feline panleukopenia virus |
| >MK671165.1 | Jilin, China | Feline panleukopenia virus |
| >MK671164.1 | Jilin, China | Feline panleukopenia virus |
| >MK671163.1 | Jilin, China | Feline panleukopenia virus |
| >MK671162.1 | Jilin, China | Feline panleukopenia virus |
| >MK671161.1 | Jilin, China | Feline panleukopenia virus |
| >MK671160.1 | Jilin, China | Feline panleukopenia virus |
| >MK671159.1 | Jilin, China | Feline panleukopenia virus |
| >MK671158.1 | Jilin, China | Feline panleukopenia virus |
| >MK671157.1 | Jilin, China | Feline panleukopenia virus |

Table S2 Recombination analysis sequence

| NO. | Strains | Accession no. | Genetic type | Submitted year | Origin |
| --- | --- | --- | --- | --- | --- |
| 1 | Shangdong3 | GU392247 | MEV | 2010 | China |
| 2 | MEV/LN-10 | HQ694567 | MEV | 2011 | China |
| 3 | SD12/01 | KC713592 | MEV | 2013 | China |
| 4 | Abashiri | D00765 | MEV | 2007 | Japan |
| 5 | MEVB | FJ592174 | MEV | 2009 | China |
| 6 | SMPV-11 | KP008112 | MEV | 2015 | China |
| 7 | MEV-LHV | KT899745 | MEV | 2015 | China |
| 8 | MEV-L | KT899746 | MEV | 2015 | China |
| 9 | MEV-SDNH | JX535284 | MEV | 2013 | China |
| 10 | FPV-VT2020 | MN270937 | FPV | 2019 | Thailand |
| 11 | CU-4 | M38246 | FPV | 1996 | USA |
| 12 | 193/70 | X55115 | FPV | 2005 | USA |
| 13 | XJ-1 | EF988660 | FPV | 2007 | China |
| 14 | FPV-8a.us.89 | EU659113 | FPV | 2008 | USA |
| 15 | FPV-4.us.64 | EU659112 | FPV | 2008 | USA |
| 16 | FPV-3.us.67 | EU659111 | FPV | 2008 | USA |
| 17 | FPV-kai.us.06 | EU659115 | FPV | 2008 | USA |
| 18 | FPV-8b.us.89 | EU659114 | FPV | 2008 | USA |
| 19 | 933/07 | EU360958 | FPV | 2007 | Hungary |
| 20 | NIG/2021/265.21-76 | ON063555 | CPV-2a | 2022 | Italy |
| 21 | CPV-N | M19296 | CPV-2 | 1995 | USA |
| 22 | CPV-6.us.80 | EU659117 | CPV-2 | 2009 | USA |
| 23 | CPV-b | M38245 | CPV-2 | 1996 | USA |
| 24 | CPV-VT0561 | MN270938 | CPV-2a | 2019 | Thailand |
| 25 | CPV-13.us.81 | EU659118 | CPV-2a | 2008 | USA |
| 26 | Y1 | D26079 | CPV-2a | 2002 | Japan |
| 27 | cpv/nj01/06 | EU310373 | CPV-2a | 2008 | China |
| 28 | CPV_0208_HU | ON185542 | CPV-2b | 2022 | Hungary |
| 29 | Vaccine | KY083091 | CPV-2b | 2016 | Singapore |
| 30 | CPV-2b/850/2017 | MK348104 | CPV-2b/ | 2018 | Italy |
| 31 | 12B | PP049248 | CPV-2c | 2023 | Iran |
| 32 | CPV-VT3761 | MN270961 | CPV-2c | 2019 | Thailand |
| 33 | HN4AA | MK357735 | CPV-2c | 2019 | China |
| 34 | HRB-A6 | KT156832 | CPV-2c | 2015 | China |
| 35 | CDD04 | MZ857181 | CPV-2c | 2021 | China |
| 36 | TS-F7 | MZ576576 | CPV-2c | 2021 | China |
| 37 | CPV2a | AJ564427 | new CPV-2a | 2004 | India |
| 38 | CPV-193 | AY742932 | new CPV-2b | 2005 | USA |
| 39 | CPV-339 | AY742933 | new CPV-2a | 2005 | New Zealand |
| 40 | CPV-447 | AY742934 | new CPV-2b | 2005 | USA |
| 41 | CPV-U6 | AY742935 | new CPV-2a | 2005 | Germany |
| 42 | CPV-395 | AY742936 | new CPV-2b | 2005 | USA |
| 43 | B-2004 | EF011664 | new CPV-2a | 2006 | China |
| 44 | CPV-410.us.00 | EU659119 | new CPV-2b | 2008 | USA |
| 45 | CPV-411a.us.98 | EU659120 | new CPV-2b | 2008 | USA |
| 46 | CPV-411b.us.98 | EU659121 | new CPV-2b | 2008 | USA |

**Figure legend (supplementary material)**

Figure S1. Recombinant analysis of CPV-JL-06/China/2020

A: Results of RDP recombination analysis of CPV-JL-06/China/2020 isolate; B: Phylogenetic analysis of CPV-2 VP2 isolates based on the nt1-920; C: Phylogenetic analysis of CPV-2 VP2 isolates based on the nt920-1755.

Figure S2. Results of CPV-JL-7 recombination analysis and p-value results.
